# The Evaluation of Laboratory Parameters as Predictors of Disease Severity and Mortality in COVID-19 Patients: A Retrospective Study From a Tertiary Care Hospital in India

**DOI:** 10.7759/cureus.40273

**Published:** 2023-06-11

**Authors:** Tanima Dwivedi, Apurva Raj, Nupur Das, Ritu Gupta, Nishkarsh Gupta, Pawan Tiwari, Biswajeet Sahoo, Hari Krishna Raju Sagiraju, Prashant Sirohiya, Brajesh Ratre, Arunmozhimaran Elavarasi, Anant Mohan, Sushma Bhatnagar

**Affiliations:** 1 Department of Laboratory Oncology, All India Institute of Medical Sciences, New Delhi, IND; 2 Department of Onco-Anesthesiology and Palliative Medicine, All India Institute of Medical Sciences, New Delhi, IND; 3 Department of Pulmonary, Critical Care, and Sleep Medicine, All India Institute of Medical Sciences, New Delhi, IND; 4 Department of Preventive Oncology, All India Institute of Medical Sciences, New Delhi, IND; 5 Department of Neurology, All India Institute of Medical Sciences, New Delhi, IND

**Keywords:** mortality, severity, biomarkers, laboratory parameters, covid-19, sars-cov-2

## Abstract

Background

Severe acute respiratory syndrome coronavirus 2 (SARS-CoV-2) infection affects and alters various laboratory parameters that are predictors of disease severity and mortality, and hence, their prompt identification can aid in patient triaging and resource allocation.

Objectives

A retrospective study was conducted on 7416 admitted coronavirus disease 2019 (COVID-19) patients from 20 March 2020 to 9 August 2021 to identify crucial laboratory biomarkers as predictors of disease severity and outcome; also, their optimal cutoffs were also calculated. A comparison of laboratory markers between both COVID-19 waves was also performed.

Results

The majority of patients had mild disease (4295/7416, 57.92%), whereas 1262/7416 (17.02%) had severe disease. The overall fatal outcome was reported in 461 (6.22%) patients. Predictors for mortality were age (>52 years), albumin/globulin (A/G) ratio (≤1.47), chloride (≤101 mmol/L), ferritin (>483.89 ng/mL), lactate dehydrogenase (LDH) (>393 U/L), procalcitonin (>0.10 ng/mL), interleukin-6 (IL-6) (>8.8 pg/mL), fibrinogen (>403 mg/dL), international normalized ratio (INR) (>1.18), and D-dimer (>268 ng/mL). Disease severity predictors were neutrophils (>81%), lymphocyte (≤25.4%), absolute lymphocyte count (ALC) (≤1.38×10^3^/µL), absolute eosinophil count (AEC) (≤0.03×10^3^/µL), total bilirubin (TBIL) (≥0.51 mg/dL), A/G ratio (≤1.49), albumin (≤4.2 g/dL), ferritin (≥445.4 mg/dL), LDH (≥479 U/L), IL-6 (≥28.6 pg/mL), C-reactive protein/albumin (CRP/ALB) ratio (≥1.78), D-dimer (≥237 ng/mL), and fibrinogen (≥425 mg/dL). The majority of patients admitted in the second wave were older and had severe disease, increased fatality, and significantly deranged laboratory parameters than first wave patients.

Conclusion

Our findings suggested that several biomarkers are crucial for both severe disease and mortality in COVID-19 patients. Ferritin, LDH, IL-6, A/G ratio, fibrinogen, and D-dimer are important biomarkers for both severity and mortality, and when combined, they provide valuable information for patient monitoring and triaging. In addition to these, older age, INR, chloride, and procalcitonin are also significant risk factors for mortality. For severe COVID-19, TBIL, CRP/ALB, albumin, neutrophil percentage, lymphocyte percentage, ALC, and AEC are also important biomarkers. According to the study, the majority of the baseline laboratory parameters associated with COVID-19 mortality and severe disease were significantly higher during the second wave, which could be one of the possible causes for the high mortality rate in India during the second wave. So, the combination of all these parameters can be a powerful tool in emergency settings to improve the efficacy of treatment and prevent mortality, and the planning of subsequent waves should be done accordingly.

## Introduction

Severe acute respiratory syndrome coronavirus 2 (SARS-CoV-2) was first reported on 31 December 2019 in Wuhan, China. Thereafter, it rapidly spread worldwide and was declared a pandemic on 11 March 2020 by the World Health Organization (WHO) [1]. India reported its first case of coronavirus disease 2019 (COVID-19) on 27 January 2020, and as of 12 September 2021, more than three million people in India have been infected, and more than 0.4 million patients have died [2]. In the span of one year, India has witnessed two surges or waves of COVID-19 cases. The first wave was from July 2020 to January 2021, with a peak in mid-September 2020, whereas the second wave started rising in April 2021, peaked in May 2021, and subsided by June 2021 [2]. The clinical presentation of COVID-19 varies from patient to patient and ranges from asymptomatic to severe form of the disease [3].

Various hypotheses such as direct viral toxicity, dysregulation of renin-angiotensin-aldosterone system (RAAS), endothelial cell damage and thrombo-inflammation, and dysregulation of the immune response caused by the release of cytokines or secondary to sepsis have been reported [4]. Laboratory parameters can provide prognostic information that can have a significant impact on patient care and mortality. Increased levels of certain inflammatory cytokines and markers have been associated with severe disease and unfavorable outcomes. However, most of the data have emerged from relatively smaller studies, and also, the results can be inconsistent due to geographical disparity and mutation of viruses [5-7]. So, there is an urgent need for biomarkers for prompt diagnosis, risk stratification, and optimizing hospitalization admission and monitoring especially in developing countries such as India where there are disparities between the supply and demand of medical facilities [7].

The aim of this study was to identify crucial routine laboratory biomarkers, as well as to define their optimal cutoffs that can predict severe disease and unfavorable outcomes in COVID-19 patients. Additionally, laboratory biomarkers between the two waves were also compared to identify determinants for severe disease and mortality.

## Materials and methods

**Figure 1:**
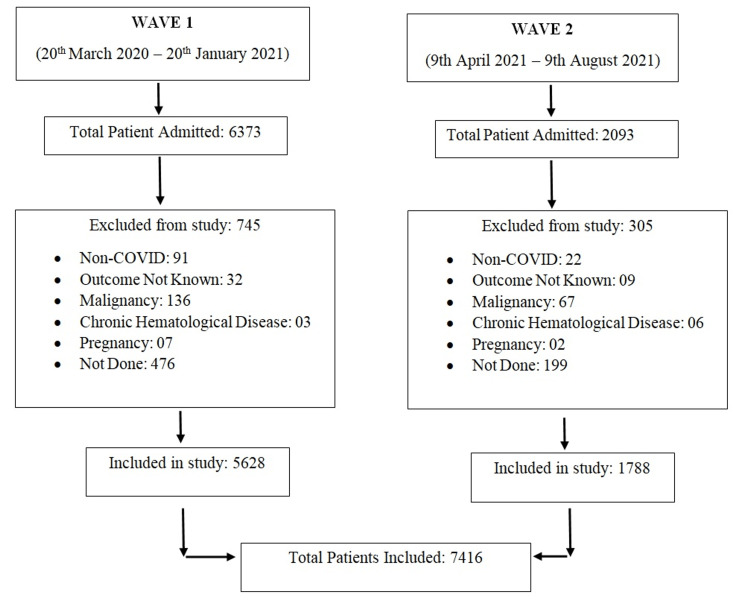
Flowchart depicting the enrolment of COVID-19 patients COVID-19: coronavirus disease 2019

This retrospective study was conducted from 20 March 2020 to 9 August 2021 at the National Cancer Institute (NCI), All India Institute of Medical Sciences (AIIMS), which was converted into a designated COVID-19 treatment facility during both COVID-19 waves. All hospitalized laboratory-confirmed COVID-19 patients by real-time reverse transcription-polymerase chain reaction (RT-PCR) or cartridge-based nucleic acid amplification test (CBNAAT) or rapid antigen test (RAT) were included in the study. Pregnant females, children (age: <18 years), those with a condition affecting laboratory parameters such as those with malignancy or chronic hematological diseases (thalassemia, sickle cell disease, etc.), and those whose outcome was not known (transferred to another medical facility, discharge on request {DOR}, or left against medical advice {LAMA}) were excluded from the study (Figure 1).

The patient’s characteristics at hospital admission were retrieved from the hospital information system. The severity of COVID-19 was classified as asymptomatic, mild, moderate (respiratory rate {RR}, ≥24 breaths/minute; oxygen saturation {SpO_2_}, 90% to ≤93% on room air {RA}), and severe (RR, >30 breaths/minute; severe respiratory distress; and SpO_2_, <90% on RA), as per the criteria of the Ministry of Health and Family Welfare at the time of admission [8].

Laboratory biomarkers

All these parameters were done as a baseline investigation at the time of admission. Fifty-five laboratory parameters were included in the study. Details of laboratory biomarkers are shown below.

The hematological biomarkers are as follows (equipment: ADVIA 2120i hematology analyzer, Siemens AG, Munich, Germany): hemoglobin (Hb), hematocrit (HCT), red blood cell count (RBC), total leucocyte count (TLC), platelet (PLT) count, mean corpuscular volume (MCV), mean corpuscular hemoglobin (MCH), mean corpuscular hemoglobin concentration (MCHC), red cell distribution width (RDW), neutrophils (percentage and absolute neutrophil counts {ANC}), lymphocytes (percentage and absolute lymphocyte counts {ALC}), monocytes (percentage and absolute monocyte counts {AMC}), eosinophils (percentage and absolute eosinophil counts {AEC}), basophils (percentage and absolute basophil counts {ABC}).

Calculated parameters are neutrophil-lymphocyte ratio (NLR; calculated by ANC×ALC), platelet-to-lymphocyte ratio (PLR; calculated by PLT×ALC), lymphocyte-to-monocyte ratio (LMR; calculated by ALC×AMC), neutrophil-to-monocyte ratio (NMR; calculated by ANC×AMC), systemic immune-inflammation index (SII; calculated by PLT×ANC/ALC).

The coagulation biomarkers are as follows (equipment: ACL TOP 750 LAS Hemostasis Testing System, Instrumentation Laboratory, Bedford, MA): prothrombin time (PT), international normalized ratio (INR), activated partial thromboplastin time (aPTT), D-dimer, and fibrinogen.

The biochemical biomarkers are as follows (equipment: ADVIA 1800 biochemistry analyzer, Siemens AG, Munich, Germany): total bilirubin (TBIL), direct bilirubin (DBIL), indirect bilirubin (IBIL), alanine aminotransferase (ALT/serum glutamic pyruvic transaminase {SGPT}), aspartate aminotransferase (AST/serum glutamic oxaloacetic transaminase {SGOT}), alkaline phosphatase (ALP), total protein (TP), albumin (ALB), globulin, albumin/globulin ratio (A/G), urea, creatinine, calcium, phosphorus, sodium, potassium, chloride, and uric acid.

The inflammatory biomarkers are as follows (equipment: ADVIA Centaur XPT Immunoassay System and ADVIA 1800 biochemistry analyzer, Siemens AG, Munich, Germany): ferritin, procalcitonin, interleukin-6 (IL-6), lactate dehydrogenase (LDH), C-reactive protein (CRP). The calculated parameter is C-reactive protein (CRP)/albumin ratio (CRP/ALB).

The glycemic biomarkers are as follows (equipment: ADVIA 1800 biochemistry analyzer, Siemens AG, Munich, Germany): serum random glucose (Glu-R) and glycated hemoglobin (HbA1c).

Statistical analysis

The normality of the data distribution was determined by the Kolmogorov-Smirnov test. Categorical variables were represented as frequency and percentage and compared using chi-square tests. The continuous variables in this study were represented with median and interquartile range. Laboratory biomarker variance was comprehended according to laboratory reference ranges as in normal, elevated, or reduced.

To identify laboratory biomarkers as a predictor of COVID-19 mortality and severity, a three-step approach was used. First, pairwise comparison for continuous variables was performed by Mann-Whitney test (survivors and non-survivors). Kruskal-Wallis H test was used to compare severity groups (asymptomatic, mild, moderate, and severe). Then, optimal predictive cutoff points for the variables were defined by receiver operating characteristic (ROC) curve, using the area under curve (AUC). After categorizing the biomarkers according to their optimal cutoff points, univariate analysis was performed to screen out independent variables to be used in the binary/multinomial logistic regression, for odds ratios (OR) and 95% confidence intervals. Imputation was not done for missing data. Nagelkerke’s R-square tests were used to assess the quality and adequacy of both models (0.2 is acceptable, 0.4 is good, and 0.5 is a very good model). A statistically significant difference was detected for variables with a two-sided p value of <0.05.

Inter- and intra-COVID-19 wave comparison (first versus second) was done for laboratory biomarker-related variables and patient characteristics with respect to disease severity (asymptomatic, mild, moderate, and severe) and (recovered versus mortality) by Mann-Whitney test for continuous variable and categorical variables by chi-square tests.

Statistical analyses were performed with Statistical Package for Social Sciences (SPSS) version 16.0 (SPSS Statistics, Chicago, IL) and MedCalc software (MedCalc Software Ltd, Ostend, Belgium).

## Results

A total of 7416 patients were included in the study (Figure 1). The median age of the patients was 40 years (range: 01-97 years). The majority of patients were males (5485, 73.96%) followed by females (1927, 25.98%) and others (four, 0.05%). At the time of admission, 4295 (57.92%) had mild disease, whereas 1262 (17.02%) had severe disease, and 954 (12.86%) were asymptomatic followed by patients with moderate disease (905, 12.20%). Regarding disease outcome, 6955 (93.78%) were survivors, and 461 (6.22%) were non-survivors. The average duration of the hospital stay was 10 days (range: 3-22 days).

Variation of baseline laboratory biomarkers among COVID-19 patients

Most of the laboratory biomarkers in patients were in the normal range. LDH (3012, 63.2%), CRP (3577, 58.8%), IL-6 (1945, 58%), CRP/ALB (3699, 60.9%), fibrinogen (2763, 50.2%), and HbA1c (2194, 56.6%) were raised in the majority of patients (Table 1).

**Table 1:** Variation of laboratory biomarkers in COVID-19 patients N, number of patients; IQR, interquartile range; Hb, hemoglobin; HCT, hematocrit; RBC, red blood cell; TLC, total leucocyte count; PLT, platelet; MCV, mean corpuscular volume; MCH, mean corpuscular hemoglobin; MCHC, mean corpuscular hemoglobin concentration; RDW: red cell distribution width; ANC, absolute neutrophil counts; ALC, absolute lymphocyte counts; AMC, absolute monocyte counts; AEC, absolute eosinophil counts; ABC, absolute basophil counts; NLR, neutrophil-lymphocyte ratio; PLR, platelet-to-lymphocyte ratio; LMR, lymphocyte-to-monocyte ratio; NMR, neutrophil-to-monocyte ratio; SII, systemic immune-inflammation index; PT, prothrombin time; INR, international normalized ratio; aPTT, activated partial thromboplastin time; TBIL, total bilirubin; DBIL, direct bilirubin; IBIL, indirect bilirubin; ALT/SGPT, alanine aminotransferase/serum glutamic pyruvic transaminase; AST/SGOT, aspartate aminotransferase/serum glutamic oxaloacetic transaminase; ALP, alkaline phosphatase; TP, total protein; ALB, albumin; A/G, albumin/globulin; CRP/ALB, C-reactive protein/albumin; Glu-R, serum random glucose; HbA1c, glycated hemoglobin; COVID-19, coronavirus disease 2019; LDH, lactate dehydrogenase

Variables (Units)	Reference Range	N (%)	Median (IQR)	Within the Range (%)	Elevated (%)	Reduced (%)
Hb (g/dL)	12-17	7361 (99.3)	13.3 (12-14.6)	5330 (72.4)	87 (1.2)	1944 (26.4)
HCT (%)	40-50	7361 (99.3)	42.1 (38.3-45.6)	4426 (60.1)	336 (4.6)	2599 (35.3)
RBC (10^6^/µL)	4.5-5.5	7361 (99.3)	4.7 (4.2-5.1)	3843 (52.2)	631 (8.6)	2887 (39.2)
MCV (fL)	83-101	7361 (99.3)	90.5 (86.2-94.8)	5679 (77.1)	589 (8.0)	1093 (14.8)
MCH (pg)	27-32	7361 (99.3)	28.8 (27.1-30.2)	4953 (67.3)	651 (8.8)	1757 (23.9)
MCHC (g/dL)	31.5-34.5	7361 (99.3)	31.64 (30.8-32.4)	3976 (54.0)	61 (0.8)	3324 (45.2)
RDW (%)	11.6-15	7361 (99.3)	14.1 (13.4-15.1)	5227 (71.0)	2122 (28.8)	12 (0.2)
PLT (10^3^/µL)	150-400	7361 (99.3)	209 (157-277)	5289 (71.9)	482 (6.5)	1590 (21.6)
TLC (10^3^/µL)	4-10	7361 (99.3)	5.97 (4.6-8)	5317 (72.2)	1018 (13.8)	1026 (13.9)
Neutrophils (%)	40-80	7213 (97.3)	60.7 (50.8-74)	5444 (75.5)	1310 (18.2)	459 (6.4)
ANC (10^3^/µL)	2-7	7213 (97.3)	3.49 (2.4-5.4)	4975 (69.0)	1188 (16.5)	1050 (14.6)
Lymphocytes (%)	20-40	7213 (97.3)	26.6 (15.7-34.8)	3889 (53.9)	925 (12.8)	2399 (33.3)
ALC (10^3^/µL)	1-3	7213 (97.3)	1.42 (1-1.9)	5013 (69.5)	265 (3.7)	1935 (26.8)
Eosinophils (%)	0-7	7213 (97.3)	1 (0.2-2.4)	5304 (73.5)	323 (4.5)	1586 (22.0)
AEC (10^3^/µL)	0.02-0.5	7213 (97.3)	0.06 (0-0.10)	4706 (65.2)	241 (3.3)	2266 (31.4)
Monocytes (%)	3-11	7213 (97.3)	6.3 (4.8-7.8)	6467 (89.7)	365 (5.1)	381 (5.3)
AMC (10^3^/µL)	0.2-1	7213 (97.3)	0.38 (0.3-0.5)	6541 (90.7)	129 (1.8)	543 (7.5)
Basophils (%)	0-2	7213 (97.3)	0.6 (0.3-1.1)	6413 (88.9)	776 (10.8)	24 (0.3)
ABC (10^3^/µL)	0-0.1	7213 (97.3)	0.04 (0-0.1)	6060 (84.0)	1124 (15.6)	29 (0.4)
NLR (Ratio)	0.86-2.44	7213 (97.3)	2.3 (1.5-4.7)	3477 (48.2)	3367 (46.7)	369 (5.1)
PLR (Ratio)	88.7-176.1	7213 (97.3)	143.9 (98.9-227.4)	3172 (44.0)	2682 (37.2)	1359 (18.8)
LMR (Ratio)	3.6-6.9	7213 (97.3)	3.9 (2.5-5.5)	3191 (44.2)	792 (11.0)	3230 (44.8)
SII (Ratio)	171.3-998	7213 (97.3)	474.2 (272.3-1092.3)	4526 (62.7)	1933 (26.8)	754 (10.5)
NMR (Ratio)	3.1-17.1	7213 (97.3)	9.38 (6.8-14)	5902 (81.8)	1228 (17.0)	83 (1.2)
TBIL (mg/dL)	0.3-1.2	7387 (99.6)	0.55 (0.4-0.8)	6026 (81.6)	534 (7.2)	827 (11.2)
DBIL (mg/dL)	<0.3	7391 (99.7)	0.2 (0.19-0.30)	5702 (77.1)	1688 (22.8)	1 (0.0)
IBIL (mg/dL)	<0.9	7387 (99.6)	0.34 (0.20-0.50)	7013 (94.9)	354 (4.8)	20 (0.3)
SGPT/ALT (U/L)	10-49	7390 (99.6)	39 (24-66)	4455 (60.3)	2837 (38.3)	98 (1.3)
SGOT/AST (U/L)	<34	7391 (99.7)	36 (27-55)	3240 (43.8)	4150 (56.1)	1 (0.0)
TP (g/dL)	5.7-8.2	7391 (99.7)	6.73 (6.30-7.10)	6884 (93.1)	46 (0.6)	461 (6.2)
ALP (IU)	46-116	7390 (99.6)	83 (67-105)	5785 (78.3)	1347 (18.2)	258 (3.5)
Albumin (g/dL)	3.2-4.8	7391 (99.7)	4.2 (3.8-4.4)	6545 (88.6)	405 (5.5)	441 (6.0)
Globulin (g/dL)	2.5-3.4	7391 (99.7)	2.56 (2.3-2.8)	3840 (52.0)	266 (3.6)	3285 (44.4)
A/G Ratio (Ratio)	1.2-2.2	7391 (99.7)	1.62 (1.4-1.8)	6552 (88.6)	217 (2.9)	622 (8.4)
Urea (mg/dL)	10-50	7368 (99.3)	23.4 (17.1-32.1)	5866 (79.6)	851 (11.5)	651 (8.8)
Creatinine (mg/dL)	0.5-1.1	7375 (99.4)	0.76 (0.6-0.9)	6189 (83.9)	597 (8.1)	589 (8.0)
Calcium (mg/dL)	8.7-10.4	7375 (99.4)	8.83 (8.4-9.2)	4238 (57.5)	21 (0.3)	3116 (42.3)
Phosphorus (mg/dL)	2.4-5.1	7373 (99.4)	2.95 (2.5-3.5)	5564 (75.5)	214 (2.9)	1595 (21.6)
Sodium (mmol/L)	132-146	7375 (99.4)	139 (137-141)	6849 (92.9)	140 (1.9)	386 (5.2)
Potassium (mmol/L)	3.5-5.5	7374 (99.4)	4.3 (4-4.7)	6603 (89.5)	318 (4.3)	453 (6.1)
Chloride (mmol/L)	99-109	7375 (99.4)	104 (101-106)	5896 (79.9)	459 (6.2)	1020 (13.8)
Uric Acid (mg/dL)	3.1-7.8	7373(99.4)	4.9 (3.9-5.9)	6244 (84.7)	431 (5.8)	698 (9.5)
Ferritin (ng/mL)	10-291	4572 (61.7)	198.1 (81.4-538.3)	2587 (56.6)	1661 (36.3)	324 (7.1)
LDH (U/L)	120-246	4764 (64.2)	283 (222-416)	1746 (36.6)	3012 (63.2)	6 (0.1)
CRP (mg/dL)	0-0.5	6081 (82)	0.95 (0.1-4.6)	2250 (37.0)	3577 (58.8)	254 (4.2)
Procalcitonin (ng/mL)	<0.1	2589 (34.9)	0.03 (0-0.1)	978 (37.8)	579 (22.4)	1032 (39.9)
IL-6 (pg/mL)	0-4.4	3354 (45.2)	6.2 (1.7-23.7)	1031 (30.7)	1945 (58.0)	378 (11.3)
CRP/ALB (Ratio)	0-0.1	6073 (81.9)	0.23 (0-1.2)	1563 (25.7)	3699 (60.9)	811 (13.4)
PT (Seconds)	10.2-13.2	5620 (75.8)	12.1 (11.4-13)	4355 (77.5)	1148 (20.4)	117 (2.1)
INR (Ratio)	<1.1	5613 (75.5)	1.04 (1-1.1)	3867 (68.9)	1746 (31.1)	0 (0.0)
aPTT (Seconds)	25.4-38.4	5538 (74.7)	32.3 (30-35)	4884 (88.2)	535 (9.7)	119 (2.1)
D-Dimer (ng/mL)	<500	5531 (74.6)	145 (78-291)	4712 (85.2)	806 (14.6)	13 (0.2)
Fibrinogen (mg/dL)	180-350	5499 (74.2)	350 (288-436)	2656 (48.3)	2763 (50.2)	80 (1.5)
Glu-R (mg/dL)	74-140	6642 (89.6)	97 (81-134)	4376 (65.9)	1559 (23.5)	707 (10.6)
HbA1c (%)	<5.7	3876 (52.3)	5.8 (5.4-7)	1678 (43.3)	2194 (56.6)	4 (0.1)

Predictors of COVID-19 outcome (survivors versus non-survivors)

Out of 7416 patients, 461 patients were non-survivors (6.22%). Patients with unfavorable outcomes of death were significantly older and had shorter hospital stay. The majority of non-survivors were males (316 {68.5%}) and suffering from severe COVID-19 disease (451 {97.8%}) (Table 2).

**Table 2:** Comparison of clinical and laboratory parameters among COVID-19 patient survivors and non-survivors N, number of patients; IQR, interquartile range; Hb, hemoglobin; HCT, hematocrit; RBC, red blood cell, TLC, total leucocyte count; PLT, platelet; MCV, mean corpuscular volume; MCH, mean corpuscular hemoglobin; MCHC, mean corpuscular hemoglobin concentration; RDW, red cell distribution width; ANC, absolute neutrophil counts; ALC, absolute lymphocyte counts; AMC, absolute monocyte counts; AEC, absolute eosinophil counts; ABC, absolute basophil counts; NLR, neutrophil-lymphocyte ratio; PLR, platelet-to-lymphocyte ratio; LMR, lymphocyte-to-monocyte ratio; NMR, neutrophil-to-monocyte ratio; SII, systemic immune-inflammation index; PT, prothrombin time; INR, international normalized ratio; aPTT, activated partial thromboplastin time; TBIL, total bilirubin; DBIL, direct bilirubin; IBIL, indirect bilirubin; ALT/SGPT, alanine aminotransferase/serum glutamic pyruvic transaminase; AST/SGOT, aspartate aminotransferase/serum glutamic oxaloacetic transaminase; ALP, alkaline phosphatase; TP, total protein; ALB, albumin; A/G, albumin/globulin; CRP/ALB, C-reactive protein/albumin; Glu-R, serum random glucose; HbA1c, glycated hemoglobin; COVID-19, coronavirus disease 2019; LDH, lactate dehydrogenase

		Survivors (7416)	Survivors	Non-survivor (461)	Non-survivor	Mann-Whitney Test
Variables (Unit)	Total N (%)	N (%)	Median (IQR)	N (%)	Median (IQR)	P Value
Gender						
Male	5485(74)	5169 (69.8)	-	316 (68.5)	-	0.02
Female	1927 (26)	1782 (24.1)	-	145 (31.5)	-	
Others	4 (0.1)	4 (0.1)	-	0 (0)	-	
Severity						
Asymptomatic	954 (12.9)	954 (13.7)	-	0 (0)	-	<0.001
Mild	4295 (57.9)	4294 (61.7)	-	1 (0.2)	-	
Moderate	905 (12.2)	896 (12.9)	-	9 (2)	-	
Severe	1262 (17)	811 (11.7)	-	451 (97.8)	-	
Age (Years)	7416 (100)	6955 (93.8)	39 (30-52)	461 (6.2)	60 (50-70)	<0.001
Duration (Days)	7416 (100)	6955 (93.8)	10 (8-12)	461 (6.2)	9 (5-15)	0.001
Hb (g/dL)	7361 (99.3)	6904 (93.8)	13.4 (12-15)	457 (6.2)	12.7 (11-14.1)	<0.001
HCT (%)	7361 (99.3)	6904 (93.8)	42.2 (38.4-46)	457 (6.2)	40.7 (35.8-44.6)	<0.001
RBC (10^6^/µL)	7361 (99.3)	6904 (93.8)	4.7 (4.3-5)	457 (6.2)	4 (3.9-4.9)	<0.001
MCV (fL)	7361 (99.3)	6904 (93.8)	90.4 (86.2-95)	457 (6.2)	91.4 (86.2-96.8)	0.002
MCH (pg)	7361 (99.3)	6904 (93.8)	28.8 (27.1-30)	457 (6.2)	28.6 (26.8-30.3)	0.31
MCHC (g/dL)	7361 (99.3)	6904 (93.8)	31.7 (30.8-32)	457 (6.2)	31.1 (30.2-32)	<0.001
RDW (%)	7361 (99.3)	6904 (93.8)	14.1 (13.4-15)	457 (6.2)	14.7 (13.8-16)	<0.001
PLT (10^3^/µL)	7361 (99.3)	6904 (93.8)	209 (157-275)	457 (6.2)	218 (160-306)	0.02
TLC (10^3^/µL)	7361 (99.3)	6904 (93.8)	5.83 (4.6-8)	457 (6.2)	11 (7.8-15.7)	<0.001
Neutrophils (%)	7213 (97.3)	6763 (93.8)	59.5 (50.2-71)	450 (6.2)	88.1 (83.4-91)	<0.001
Lymphocytes (%)	7213 (97.3)	6763 (93.8)	27.6 (18.2-35)	450 (6.2)	5.6 (3.4-9.2)	<0.001
Eosinophils (%)	7213 (97.3)	6763 (93.8)	1.1 (0.3-3)	450 (6.2)	0.1 (0-0.1)	<0.001
Monocytes (%)	7213 (97.3)	6763 (93.8)	6.4 (5-8)	450 (6.2)	4 (3.1-5.1)	<0.001
Basophils (%)	7213 (97.3)	6763 (93.8)	0.6 (0.4-1)	450 (6.2)	0.3 (0.2-0.5)	<0.001
ANC (10^3^/µL)	7213 (97.3)	6763 (93.8)	3.3 (2.4-5)	450 (6.2)	9.9 (6.5-14.3)	<0.001
ALC (103/µL)	7213 (97.3)	6763 (93.8)	1.5 (1-2)	450 (6.2)	0.62 (0.4-0.9)	<0.001
AEC (10^3^/µL)	7213 (97.3)	6763 (93.8)	0.1 (0-0.18)	450 (6.2)	0.01 (0-0.01)	<0.001
AMC (10^3^/µL)	7213 (97.3)	6763 (93.8)	0.38 (0.3-1)	450 (6.2)	0.46 (0.3-0.7)	<0.001
ABC (10^3^/µL)	7213 (97.3)	6763 (93.8)	0.037 (0-0.04)	450 (6.2)	0.033 (0-0.1)	<0.001
NLR (Ratio)	7213 (97.3)	6763 (93.8)	2.14 (1.4-4)	450 (6.2)	15.5 (9.1-27.1)	<0.001
PLR (Ratio)	7213 (97.3)	6763 (93.8)	138.7 (97.1-209.9)	450 (6.2)	338.2 (214.7-559.1)	<0.001
LMR (Ratio)	7213 (97.3)	6763 (93.8)	4.04 (2.7-5.6)	450 (6.2)	1.43 (0.8-2.3)	<0.001
SII (Ratio)	7213 (97.3)	6763 (93.8)	444.8 (262.8-894.4)	450 (6.2)	3268.3 (1601.6-7011.9)	0.61
NMR (Ratio)	7213 (97.3)	6763 (93.8)	9.09 (6.6-13)	450 (6.2)	21.6 (16.3-28.7)	<0.001
TBIL (mg/dL)	7387 (99.6)	6927 (93.8)	0.55 (0.4-0.8)	460 (6.2)	0.6 (0.4-0.8)	0.95
DBIL (mg/dL)	7391 (99.7)	6931 (93.8)	0.2 (0.2-0.3)	460 (6.2)	0.3 (0.2-0.4)	<0.001
IBIL (mg/dL)	7387 (99.6)	6927 (93.8)	0.35 (0.3-0.5)	460 (6.2)	0.3 (0.2-0.4)	<0.001
SGPT/ALT (U/L)	7390 (99.6)	6930 (93.8)	38.3 (24-64.2)	460 (6.2)	47.5 (28-84)	<0.001
SGOT/AST (U/L)	7391 (99.7)	6931 (93.8)	35.5 (27-52)	460 (6.2)	60.5 (38-99.3)	<0.001
ALP (IU)	7390 (99.6)	6930 (93.8)	82 (67-103)	460 (6.2)	95 (72-136)	<0.001
TP (g/dL)	7391 (99.7)	6931 (93.8)	6.8 (6.4-7.1)	460 (6.2)	6.1 (5.6-6.6)	<0.001
Albumin (g/dL)	7391 (99.7)	6931 (93.8)	4.2 (3.9-4.5)	460 (6.2)	3.5 (3.1-3.7)	<0.001
Globulin (g/dL)	7391 (99.7)	6931 (93.8)	2.56 (2.32-2.82)	460 (6.2)	2.60 (2.30-2.96)	0.02
A/G Ratio (Ratio)	7391 (99.7)	6931 (93.8)	1.6 (1.4-1.8)	460 (6.2)	1.3 (1.1-1.5)	<0.001
Urea (mg/dL)	7368 (99.3)	6910 (93.8)	21.4 (17-30)	458 (6.2)	62.1 (44.9-92)	<0.001
Creatinine (mg/dL)	7375 (99.4)	6916 (93.8)	0.75 (0.6-0.9)	459 (6.2)	0.93 (0.7-1.4)	<0.001
Uric Acid (mg/dL)	7373 (99.4)	6914 (93.8)	4.9 (3.9-5.9)	459 (6.2)	5.4 (4.1-7.6)	<0.001
Calcium (mg/dL)	7375 (99.4)	6916 (93.8)	8.9 (8.5-9.2)	459 (6.2)	8.2 (7.8-8.6)	<0.001
Phosphorus (mg/dL)	7373 (99.4)	6914 (93.8)	2.9 (2.5-3.5)	459 (6.2)	3.1 (2.5-3.8)	<0.001
Sodium (mmol/L)	7375 (99.4)	6916 (93.8)	139.5 (138-141)	459 (6.2)	139 (135-143)	0.08
Potassium (mmol/L)	7374 (99.4)	6915 (93.8)	4.3 (4-4.7)	459 (6.2)	4.7 (4.3-5.3)	<0.001
Chloride (mmol/L)	7375 (99.4)	6916 (93.8)	104 (102-106)	459 (6.2)	103 (99-107)	0.002
Ferritin (ng/mL)	4572 (61.7)	4254 (93.1)	180 (76.1-448.3)	318 (6.9)	948.9 (514.2-1650)	<0.001
LDH (U/L)	4764 (64.2)	4342 (91.1)	270 (218-378)	422 (8.9)	639 (476.3-838.5)	<0.001
CRP (mg/dL)	6081 (82)	5635 (92.7)	0.7 (0.1-3.4)	446 (7.3)	9.98 (5-16.3)	<0.001
Procalcitonin (ng/mL)	2589 (34.9)	2332 (90.1)	0.02 (0-0.1)	257 (9.9)	0.19 (0.1-0.8)	<0.001
IL-6 (pg/mL)	3354 (45.2)	2981 (88.9)	5 (1.4-17.4)	373 (11.1)	41.9 (15.7-106.5)	<0.001
CRP/ALB (Ratio)	6073 (81.9)	5627 (92.7)	0.2 (0-0.9)	446 (7.3)	2.9 (1.4-4.9)	<0.001
aPTT (Seconds)	5538 (74.7)	5112 (92.3)	32.3 (30-34.9)	426 (7.7)	32.9 (29.4-36.7)	0.05
D-Dimer (ng/mL)	5531 (74.6)	5118 (92.6)	133.5 (74-248)	412 (7.5)	756 (369-2615.8)	<0.001
Fibrinogen (mg/dL)	5499 (74.2)	5074 (92.3)	343 (285-427)	425 (7.7)	433 (350-512)	<0.001
PT (Seconds)	5620 (75.8)	5186 (92.3)	12.1 (11.4-12.9)	434 (7.7)	12.5 (11.4-14.3)	<0.001
INR (Ratio)	5613 (75.5)	5179 (92.3)	1.04 (1-1.1)	434 (6.2)	1.07 (1-1.2)	<0.001
Glu-R (mg/dL)	6642 (89.6)	6275 (94.5)	95 (81-129)	367 (5.5)	165 (116-252.5)	<0.001
HbA1c (%)	3876 (52.3)	3544 (91.4)	5.7 (5.3-6.4)	332 (8.6)	6.4 (5.7-7.7)	<0.001

The non-survivors had lower hemoglobin, hematocrit, RBC count, MCH, and MCHC values compared to recovered patients but higher MCV and RDW values. They additionally had thrombocytosis, leucocytosis, neutrophilia, lymphopenia, eosinopenia, monocytopenia, and basopenia. NLR, PLR, and NMR were all higher in non-survivors, whereas LMR was lower (Table 2).

The liver function test (LFT) in non-survivors revealed raised DBIL and TBIL, reduced IBIL, markedly deranged liver enzymes (SGPT/ALT, SGOT/AST, and ALP), and reduced total protein, albumin, A/G ratio. Similarly, these patients had uremia, raised serum creatinine and uric acid, raised phosphorus, raised potassium, and reduced serum calcium and chloride levels. Inflammatory biomarkers such as ferritin, LDH, CRP, procalcitonin, IL-6, and CRP/ALB were markedly deranged in non-survivors (Table 2).

The coagulation biomarkers (D-dimer, fibrinogen, aPTT, PT, and INR) and glycemic biomarkers (serum random glucose and HbA1c) were also higher in non-survivors (Table 2).

Univariate analysis showed that all biomarkers were significantly associated with the outcome (Table 3). However, the binary logistic regression analysis, after adjusting other variables, revealed that the odds of unfavorable outcome were higher for age of >52 years, ferritin of >483.89 ng/mL, LDH of >393 U/L, procalcitonin of >0.10 ng/mL, IL-6 of >8.8 pg/mL, and D-dimer of >268 ng/mL, whereas A/G ratio of ≤1.47, chloride of ≤101 mmol/L, fibrinogen of >403 mg/dL, and INR of >1.18 ratio had lower odds for unfavorable outcome. Nagelkerke’s R-square tests for this model was 0.62, which can be considered as a very good model (Table 3).

**Table 3:** Predictors of COVID-19 mortality. Reference: age, ≤52 years; duration, ≤6 days; Hb, >11.8 g/dL; HCT, >36.41%; RBC, >4.16×106/µL; TLC, ≤8.61×103/µL; PLT, ≤295×103/µL; MCV, ≤95.2 fL; MCH, >27.95 pg; MCHC, >31.16 g/dL; RDW, ≤14.8%; neutrophils, ≤76.7%; lymphocytes, >12%; eosinophils, >0.2%; monocytes, >5%; basophils, >0.4%; ANC, ≤5.45×103/µL; ALC, >0.96×103/µL; AEC, >0.03×103/µL; AMC, ≤0.49×103/µL; ABC, >0.02×103/µL; NLR, ≤4.87 ratio; PLR: ≤208.38 ratio; LMR, >2.29 ratio; SII, ≤987.42 ratio; NMR, ≤14.56 ratio; TBIL, ≤0.99 mg/dL; IBIL, >0.3 mg/dL; SGPT/ALT, ≤46.9 U/L; SGOT/AST, ≤44.9 U/L; TP, >6.4 g/dL; ALP, ≤110 IU; globulin, ≤2.99 g/dL; A/G ratio, >1.47 ratio; albumin, >3.8 g/dL; urea, ≤36 mg/dL; creatinine, ≤0.97 mg/dL; calcium, >8.51 mg/dL; phosphorus, ≤3.69 mg/dL; sodium, >136 mmol/L; potassium, ≤4.5 mmol/L; chloride, >101 mmol/L; uric acid, ≤7.48 mg/dL; ferritin, ≤483.89 ng/mL; LDH, ≤393 U/L; CRP, ≤2.38 mg/dL; procalcitonin, ≤0.10 ng/mL; IL-6, ≤8.8 pg/mL; CRP/ALB, ≤0.62 ratio; aPTT, ≤35.5 seconds; D-dimer, ≤268 ng/mL; fibrinogen, ≤403 mg/dL; PT, ≤13.9 seconds; INR, ≤1.18 ratio; Glu-R, ≤121 mg/dL; HbA1c, ≤6.29 N, number of patients; IQR, interquartile range; Hb, hemoglobin; HCT, hematocrit; RBC, red blood cell; TLC, total leucocyte count; PLT, platelet; MCV, mean corpuscular volume; MCH, mean corpuscular hemoglobin; MCHC, mean corpuscular hemoglobin concentration; RDW: red cell distribution width; ANC, absolute neutrophil counts; ALC, absolute lymphocyte counts; AMC, absolute monocyte counts; AEC, absolute eosinophil counts; ABC, absolute basophil counts; NLR, neutrophil-lymphocyte ratio; PLR, platelet-to-lymphocyte ratio; LMR, lymphocyte-to-monocyte ratio; NMR, neutrophil-to-monocyte ratio; SII, systemic immune-inflammation index; PT, prothrombin time; INR, international normalized ratio; aPTT, activated partial thromboplastin time; TBIL, total bilirubin; DBIL, direct bilirubin; IBIL, indirect bilirubin; ALT/SGPT, alanine aminotransferase/serum glutamic pyruvic transaminase; AST/SGOT, aspartate aminotransferase/serum glutamic oxaloacetic transaminase; ALP, alkaline phosphatase; TP, total protein; ALB, albumin; A/G, albumin/globulin; CRP/ALB, C-reactive protein/albumin; Glu-R, serum random glucose; HbA1c, glycated hemoglobin; COVID-19, coronavirus disease 2019; LDH, lactate dehydrogenase; AUC, area under curve; AOR, adjusted odds ratio; CI, confidence interval

Variables (Unit)	AUC (95% CI)	Cutoff	P value	COR (95% CI)	P value	AOR (95% CI)	P Value
Age (Years)	0.8 (0.79-0.81)	>52	<0.001	7.8 (6.3-9.5)	<0.001	2.8 (1.6-4.9)	<0.001
Duration (Days)	0.55 (0.54-0.56)	≤6	0.0076	2.9 (2.4-3.6)	<0.001	0.9 (0.3-2.3)	0.8
Hb (g/dL)	0.6 (0.58-0.61)	≤11.8	<0.001	2.0 (1.6-2.4)	<0.001	1.7 (0.6-5.2)	0.36
HCT (%)	0.57 (0.56-0.59)	≤36.4	<0.001	1.9 (1.5-2.3)	<0.001	0.5 (0.2-1.4)	0.18
RBC (10^6^/µL)	0.59 (0.58-0.6)	≤4.16	<0.001	1.9 (1.6-2.4)	<0.001	0.9 (0.4-2.0)	0.85
MCV (fL)	0.54 (0.53-0.55)	>95.2	0.004	1.7 (1.4-2.1)	<0.001	0.9 (0.5-1.9)	0.83
MCH (pg)	0.51 (0.5-0.53)	≤27.95	0.35	1.2 (1.0-1.5)	0.04	1.1 (0.6-2.3)	0.73
MCHC (g/dL)	0.61 (0.6-0.62)	≤31.2	<0.001	2.1 (1.7-2.5)	<0.001	1.5 (0.8-2.8)	0.25
RDW (%)	0.61 (0.6-0.63)	>14.8	<0.001	2.1 (1.7-2.5)	<0.001	1.8 (0.9-3.3)	0.08
PLT (10^3^/µL)	0.53 (0.52-0.54)	>295	0.03	1.5 (1.2-1.9)	<0.001	1.0 (0.5-1.9)	0.9
TLC (10^3^/µL)	0.82 (0.81-0.83)	>8.61	<0.001	12.0 (9.7-14.9)	<0.001	1.1 (0.5-2.4)	0.86
Neutrophils (%)	0.92 (0.91-0.93)	>76.7	<0.001	42.9 (31.4-58.8)	<0.001	1.2 (0.2-6.2)	0.83
Lymphocytes (%)	0.92 (0.91-0.92)	≤12	<0.001	37.7 (28.5-49.9)	<0.001	1.4 (0.4-4.6)	0.56
Eosinophils (%)	0.87 (0.86-0.88)	≤0.2	<0.001	22.3 (16.7-29.6)	<0.001	1.5 (0.6-4.1)	0.4
Monocytes (%)	0.79 (0.78-0.8)	≤5	<0.001	8.1 (6.5-10.0)	<0.001	1.0 (0.4-2.4)	0.97
Basophils (%)	0.75 (0.74-0.76)	≤0.4	<0.001	4.8 (3.9-5.9)	<0.001	0.8 (0.4-1.5)	0.43
ANC (10^3^/µL)	0.88 (0.87-0.89)	>5.5	<0.001	19.4 (15.0-25.1)	<0.001	0.8 (0.3-1.9)	0.57
ALC (10^3^/µL)	0.85 (0.85-0.86)	≤0.9	<0.001	15.8 (12.4-20.1)	<0.001	1.5 (0.7-3.5)	0.34
AEC (10^3^/µL)	0.83 (0.82-0.84)	≤0.03	<0.001	14.7 (10.8-20.0)	<0.001	2.2 (0.8-6.1)	0.15
AMC (10^3^/µL)	0.6 (0.59-0.61)	>0.5	<0.001	2.5 (2.1-3.1)	<0.001	2.0 (1.0-4.2)	0.06
ABC (10^3^/µL)	0.56 (0.55-0.57)	≤0.02	<0.001	1.4 (1.1-1.7)	0.002	1.2 (0.6-2.3)	0.66
NLR (Ratio)	0.92 (0.91-0.93)	>4.9	<0.001	45.5 (32.3-64.0)	<0.001	1.6 (0.3-8.5)	0.59
PLR (Ratio)	0.8 (0.79-0.81)	>208.4	<0.001	9.9 (7.9-12.5)	<0.001	0.8 (0.4-2.0)	0.66
LMR (Ratio)	0.85 (0.84-0.85)	≤2.3	<0.001	12.9 (10.3-16.1)	<0.001	1.0 (0.4-2.0)	0.9
SII (Ratio)	0.88 (0.87-0.89)	>987.4	<0.001	25.3 (18.9-33.9)	<0.001	1.5 (0.5-5.0)	0.49
NMR (Ratio)	0.87 (0.86-0.876)	>14.6	<0.001	18.2 (14.3-23.3)	<0.001	2.1 (0.8-6.0)	0.16
TBIL (mg/dL)	0.5 (0.49-0.51)	>0.99	0.95	1.7 (1.3-2.1)	<0.001	1.6 (0.7-3.7)	0.29
DBIL (mg/dL)	0.63 (0.62-0.64)	>0.2	<0.001	2.7 (2.2-3.3)	<0.001	1.0 (0.5-1.9)	0.89
IBIL (mg/dL)	0.6 (0.59-0.61)	≤0.3	<0.001	2.0 (1.6-2.4)	<0.001	1.2 (0.6-2.3)	0.66
SGPT/ALT (U/L)	0.58 (0.57-0.59)	>46.9	<0.001	1.6 (1.3-1.9)	<0.001	0.6 (0.3-1.1)	0.08
SGOT/AST (U/L)	0.71 (0.7-0.72)	>44.9	<0.001	4.1 (3.4-5.1)	<0.001	1.5 (0.8-3.0)	0.22
ALP (IU)	0.6 (0.59-0.61)	>110	<0.001	2.5 (2.1-3.1)	<0.001	1.1 (0.6-2.0)	0.71
TP (g/dL)	0.76 (0.75-0.77)	≤6.4	<0.001	6.2 (5.1-7.6)	<0.001	1.7 (0.8-3.6)	0.17
Albumin (g/dL)	0.87 (0.86-0.87)	≤3.8	<0.001	16.6 (12.9-21.2)	<0.001	1.2 (0.5-2.7)	0.69
Globulin (g/dL)	0.53 (0.52-0.54)	>3.0	0.03	1.9 (1.5-2.4)	<0.001	1.4 (0.7-3.1)	0.36
A/G Ratio (Ratio)	0.77 (0.76-0.78)	≤1.5	<0.001	5.6 (4.5-6.8)	<0.001	0.5 (0.3-0.9)	0.03
Urea (mg/dL)	0.91 (0.9-0.96)	>36	<0.001	31.1 (23.6-40.9)	<0.001	2.0 (0.9-4.2)	0.07
Creatinine (mg/dL)	0.78 (0.77-0.79)	>0.97	<0.001	6.1 (5.0-7.4)	<0.001	1.0 (0.5-2.1)	0.97
Uric Acid (mg/dL)	0.59 (0.58-0.6)	>7.5	<0.001	5.7 (4.6-7.2)	<0.001	0.6 (0.3-1.4)	0.23
Calcium (mg/dL)	0.78 (0.77-0.79)	≤8.5	<0.001	6.9 (5.6-8.5)	<0.001	1.8 (1.0-3.4)	0.06
Phosphorus (mg/dL)	0.56 (0.55-0.57)	>3.7	<0.001	2.1 (1.7-2.6)	<0.001	0.8 (0.4-1.7)	0.57
Sodium (mmol/L)	0.53 (0.51-0.54)	≤136	0.18	2.7 (2.2-3.4)	<0.001	1.2 (0.6-2.2)	0.63
Potassium (mmol/L)	0.67 (0.66-0.69)	>4.5	<0.001	3.2 (2.7-3.9)	<0.001	1.1 (0.6-2.0)	0.68
Chloride (mmol/L)	0.54 (0.53-0.55)	≤101	0.016	2.2 (1.8-2.7)	<0.001	0.6 (0.3-1.0)	0.05
Ferritin (ng/mL)	0.83 (0.82-0.84)	>483.9	<0.001	12.0 (9.1-15.9)	<0.001	4.1 (2.1-8.0)	<0.001
LDH (U/L)	0.89 (0.88-0.9)	>393	<0.001	21.7 (16.3-28.9)	<0.001	2.4 (1.2-4.5)	0.01
CRP (mg/dL)	0.86 (0.86-0.87)	>2.4	<0.001	22.1 (15.9-30.6)	<0.001	2.7 (0.3-25.9)	0.39
Procalcitonin (ng/mL)	0.82 (0.8-0.83)	>0.10	<0.001	10.8 (8.1-14.3)	<0.001	2.0 (1.1-3.7)	0.03
IL-6 (pg/mL)	0.83 (0.82-0.84)	>8.8	<0.001	11.9 (8.6-16.3)	<0.001	2.9 (1.5-5.4)	<0.001
CRP/ALB (Ratio)	0.88 (0.87-0.89)	>0.6	<0.001	28.1 (19.7-40.3)	<0.001	0.8 (0.1-8.5)	0.86
aPTT (Seconds)	0.53 (0.52-0.54)	>35.5	0.1	1.9 (1.5-2.4)	<0.001	1.4 (0.7-2.7)	0.31
D-Dimer (ng/mL)	0.89 (0.88-0.9)	>268	<0.001	21.6 (16.2-28.9)	<0.001	3.2 (1.7-6.1)	<0.001
Fibrinogen (mg/dL)	0.67 (0.66-0.69)	>403	<0.001	3.6 (2.9-4.4)	<0.001	0.4 (0.2-0.7)	<0.001
PT (Seconds)	0.57 (0.56-0.59)	>13.9	<0.001	3.7 (2.9-4.6)	<0.001	1.9 (0.7-5.3)	0.21
INR (Ratio)	0.57 (0.55-0.58)	>1.2	<0.001	2.4 (2.0-3.0)	<0.001	0.4 (0.2-0.9)	0.04
Glu-R (mg/dL)	0.77 (0.76-0.78)	>121	<0.001	6.6 (5.2-8.4)	<0.001	0.9 (0.5-1.6)	0.71
HbA1c (%)	0.67 (0.66-0.69)	>6.3	<0.001	3.1 (2.5-3.9)	<0.001	1.5 (0.9-2.7)	0.15

Predictors of COVID-19 severity (asymptomatic versus mild versus moderate versus severe)

Patients with severe COVID-19 tended to be more older and male and had a longer duration of hospital stay. With increasing COVID-19 severity, most hematological parameters reduce, with the exception of RDW, TLC, neutrophils, and platelet count (Table 4).

**Table 4:** Characteristics of laboratory biomarkers between the severity groups N, number of patients; IQR, interquartile range; Hb, hemoglobin; HCT, hematocrit; RBC, red blood cell; TLC, total leucocyte count; PLT, platelet; MCV, mean corpuscular volume; MCH, mean corpuscular hemoglobin; MCHC, mean corpuscular hemoglobin concentration; RDW: red cell distribution width; ANC, absolute neutrophil counts; ALC, absolute lymphocyte counts; AMC, absolute monocyte counts; AEC, absolute eosinophil counts; ABC, absolute basophil counts; NLR, neutrophil-lymphocyte ratio; PLR, platelet-to-lymphocyte ratio; LMR, lymphocyte-to-monocyte ratio; NMR, neutrophil-to-monocyte ratio; SII, systemic immune-inflammation index; PT, prothrombin time; INR, international normalized ratio; aPTT, activated partial thromboplastin time; TBIL, total bilirubin; DBIL, direct bilirubin; IBIL, indirect bilirubin; ALT/SGPT, alanine aminotransferase/serum glutamic pyruvic transaminase; AST/SGOT, aspartate aminotransferase/serum glutamic oxaloacetic transaminase; ALP, alkaline phosphatase; TP, total protein; ALB, albumin; A/G, albumin/globulin; CRP/ALB, C-reactive protein/albumin; Glu-R, serum random glucose; HbA1c, glycated hemoglobin; LDH, lactate dehydrogenase

	Asymptomatic (954)		Mild (4295)		Moderate (905)		Severe (1262)		
	N	Median (IQR)	N	Median (IQR)	N	Median (IQR)	N	Median (IQR)	P Value
Gender									
Male	744 (77.8%)		3180 (74.1%)		639 (70.6%)		923 (73.1%)		<0.001
Female	209 (21.1%)		1108 (25.8%)		268 (29.4%)		342 (26.9%)		
Others	1(0.1%)		3 (0.1%)				0		
Outcome									
Recovered	954		4290		898		814		<0.001
Expired	0		1		9		451		
Age (Years)	954	34 (46.8-27)	4291	36 (49-29)	907	48 (59-38)	1264	54 (64-42.8)	<0.001
Duration (Days)	954	10 (12-8)	4291	10 (12-8)	907	9 (12-6)	1264	11 (15-7)	<0.001
Hb (g/dL)	945	13.8 (14.8-12.5)	4261	13.5 (14.7-12.2)	897	12.9 (14.1-11.6)	1258	12.7 (14.1-11.2)	<0.001
HCT (%)	945	43.6 (46.4-39.9)	4261	42.6 (46-38.8)	897	40.74 (44-37)	1258	40.35455 (44.4-36.1)	<0.001
RBC (10^6^/µL)	945	4.77 (5.2-4.4)	4261	4.72 (5.1-4.3)	897	4.54 (4.9-4.1)	1258	4.5 (4.9-4)	<0.001
MCV (fL)	945	90.9 (95.2-86.3)	4261	90.4 (94.6-86.3)	897	90.2 (94.3-86.2)	1258	90.7 (95.5-85.8)	0.11
MCH (pg)	945	28.84 (30.4-27.2)	4261	28.83 (30.3-27.2)	897	28.68 (30-27)	1258	28.69 (30.3-26.9)	0.42
MCHC (g/dL)	945	31.6 (32.3-30.8)	4261	31.7 (32.4-30.9)	897	31.6 (32.3-30.7)	1258	31.5 (32.2-30.6)	<0.001
RDW (%)	945	14.2 (15-13.4)	4261	14.1 (15.1-13.4)	897	14.1 (15.2-13.4)	1258	14.4 (15.5-13.6)	<0.001
PLT (10^3^/µL)	945	217 (283-163)	4261	201 (259-153)	897	230 (311-168)	1258	236 (315-165)	<0.001
TLC (10^3^/µL)	945	5.93 (7.3-4.8)	4261	5.43 (6.9-4.4)	897	7.09 (10-5)	1258	8.83 (12.5-6.1)	<0.001
Neutrophils (%)	911	54.2 (60.9-47.1)	4168	56.1 (64.5-48)	888	75.65 (84.1-65.3)	1246	83.4 (89-74.2)	<0.001
ANC (10^3^/µL)	911	3.15 (4.2-2.4)	4168	2.99 (4.1-2.2)	888	5.21 (8.2-3.3)	1246	7.21 (10.7-4.5)	<0.001
Lymphocytes (%)	911	31.8 (37.3-25.9)	4168	30.4 (37.3-23.3)	888	14.6 (23.4-8.5)	1246	9 (15.8-5)	<0.001
ALC (10^3^/µL)	911	1.82 (2.3-1.5)	4168	1.59 (2.1-1.2)	888	1.02 (1.4-0.7)	1246	0.78 (1.1-0.5)	<0.001
Eosinophils (%)	911	2.1 (3.9-1.2)	4168	1.4 (2.8-0.5)	888	0.2 (0.7-0.1)	1246	0.1 (0.3-0)	<0.001
AEC (10^3^/µL)	911	0.13 (0.2-0.1)	4168	0.08 (0.2-0)	888	0.014 (0.1-0)	1246	0.009 (0-0)	<0.001
Monocytes (%)	911	6.4 (7.8-5.2)	4168	6.7 (8.2-5.4)	888	5.6 (7.3-4.1)	1246	4.6 (6.2-3.4)	<0.001
AMC (10^3^/µL)	911	0.38 (0.5-0.3)	4168	0.37 (0.5-0.3)	888	0.397 (0.6-0.3)	1246	0.41 (0.6-0.3)	<0.001
Basophils (%)	911	0.9 (1.5-0.5)	4168	0.7 (1.3-0.4)	888	0.4 (0.7-0.2)	1246	0.3 (0.6-0.2)	<0.001
ABC (10^3^/µL)	911	0.055 (0.1-0)	4168	0.038 (0.1-0)	888	0.028 (0-0)	1246	0.029 (0.1-0)	<0.001
NLR (Ratio)	911	1.67 (2.4-1.3)	4168	1.85 (2.8-1.3)	888	5.15 (9.7-2.8)	1246	9.28 (17.6-4.7)	<0.001
PLR (Ratio)	911	119.96 (156.5-87.2)	4168	124.92 (176.8-91.1)	888	226.2 (364.4-141.7)	1246	285.7 (473.8-169.4)	<0.001
LMR (Ratio)	911	4.8 (6.3-3.7)	4168	4.39 (5.8-3.2)	888	2.59 (3.9-1.6)	1246	2 (3.3-1.2)	<0.001
SII (Ratio)	911	368.55 (563.4-235.7)	4168	369.32 (601.8-231.6)	888	1142.62 (2659.3-532.3)	1246	2139.62 (4497.1-913.1)	0.01
NMR (Ratio)	911	8.28 (10.8-6.4)	4168	8.26 (10.9-6.1)	888	13.27 (19.3-9.2)	1246	17.89 (25.4-12)	<0.001
TBIL (mg/dL)	951	0.59 (0.8-0.5)	4273	0.55 (0.8-0.4)	902	0.5 (0.7-0.4)	1261	0.53 (0.8-0.4)	<0.001
DBIL (mg/dL)	951	0.2 (0.3-0.2)	4276	0.2 (0.3-0.1)	902	0.2 (0.3-0.2)	1262	0.23 (0.3-0.2)	<0.001
IBIL (mg/dL)	951	0.4 (0.6-0.3)	4273	0.35 (0.5-0.3)	902	0.3 (0.4-0.2)	1261	0.3 (0.4-0.2)	<0.001
SGPT/ALT (U/L)	951	34.3 (55-21)	4276	36.1 (60-23.4)	901	47 (83-29)	1262	48 (84-29)	<0.001
SGOT/AST (U/L)	951	31 (42-25)	4276	34 (47.2-26)	902	44 (73.2-31)	1262	51 (81-34.5)	<0.001
ALP (IU)	951	85 (105-71)	4276	82 (101-68)	901	80 (108-63)	1262	87 (119.8-68)	<0.001
TP (g/dL)	951	6.95 (7.3-6.6)	4276	6.82 (7.2-6.5)	902	6.5 (6.9-6.1)	1262	6.3 (6.7-5.9)	<0.001
Albumin (g/dL)	951	4.4 (4.6-4.2)	4276	4.3 (4.5-4)	902	3.9 (4.2-3.6)	1262	3.6 (4-3.3)	<0.001
Globulin (g/dL)	951	2.57 (2.8-2.3)	4276	2.55 (2.8-2.3)	902	2.55 (2.9-2.3)	1262	2.6 (2.9-2.3)	0.01
A/G Ratio	951	1.71 (1.9-1.5)	4276	1.67 (1.9-1.5)	902	1.53 (1.7-1.3)	1262	1.42 (1.6-1.2)	<0.001
Urea (mg/dL)	945	19.3 (25-13)	4264	21 (26-15)	902	32 (46-22.3)	1257	43 (64.2-28)	<0.001
Creatinine (mg/dL)	947	0.74 (0.9-0.6)	4267	0.75 (0.9-0.6)	902	0.76 (0.9-0.7)	1259	0.81 (1.1-0.7)	<0.001
Uric Acid (mg/dL)	947	5.0 (6-4.1)	4265	5.0 (5.9-4)	902	4.5 (5.6-3.6)	1259	5.1 (6.3-3.8)	<0.001
Calcium (mg/dL)	947	9.12 (9.5-8.8)	4267	8.93 (9.3-8.6)	902	8.6 (8.9-8.2)	1259	8.31 (8.7-8)	<0.001
Phosphorus (mg/dL)	946	3.12 (3.7-2.7)	4267	2.91 (3.4-2.5)	902	2.9 (3.4-2.4)	1258	3 (3.6-2.5)	<0.001
Sodium (mmol/L)	947	140 (141-139)	4267	140 (141-138)	902	138 (140-136)	1259	138 (141-135)	<0.001
Potassium (mmol/L)	946	4.3 (4.6-4)	4267	4.3 (4.6-3.9)	902	4.5 (4.9-4.1)	1259	4.6 (5-4.1)	<0.001
Chloride (mmol/L)	947	105 (106-103)	4267	104 (106-102)	902	102 (105-99)	1259	102 (105-99)	<0.001
Ferritin (ng/mL)	460	94.35 (186-44.1)	2592	138.65 (299.7-61.5)	630	388.7 (871.9-184)	891	723.1 (1344.1-309.7)	<0.001
LDH (U/L)	386	222 (253.8-199)	2454	244 (300-208)	819	359 (468-275)	1105	483 (666-341)	<0.001
CRP (mg/dL)	614	0.115 (0.4-0)	3348	0.3655 (1.4-0.1)	892	3.8085 (6.5-1.7)	1227	8.781 (14.8-3.5)	<0.001
Procalcitonin (ng/mL)	189	0.01 (0-0)	1383	0.02 (0.1-0)	408	0.04 (0.1-0)	609	0.09 (0.3-0)	<0.001
IL-6 (pg/mL)	226	1.3 (3.7-0)	1639	3.8 (12-1.1)	605	8.8 (25.8-2.7)	883	20.4 (66.9-6.4)	<0.001
CRP-ALB (Ratio)	613	0.027 (0.1-0)	3344	0.086 (0.3-0)	890	0.977 (1.7-0.5)	1226	2.396 (4.1-1)	<0.001
aPTT (Seconds)	507	32.6 (35.1-30.3)	3021	32.3 (34.8-30.2)	842	31.8 (34.6-29.4)	1168	32.5 (35.9-29.6)	0.02
D-Dimer (ng/mL)	522	77 (129-51)	3042	107 (184.8-64)	823	214 (371-136.5)	1143	380 (969-205)	<0.001
Fibrinogen (mg/dL)	521	295 (339-258)	2995	322 (385-276)	820	420 (490-350)	1163	446 (520-359)	<0.001
PT (Seconds)	517	12.1 (12.8-11.6)	3089	12 (12.8-11.4)	842	12.2 (13.1-11.3)	1172	12.3 (13.6-11.4)	<0.001
INR (Ratio)	515	1.04 (1.1-1)	3088	1.04 (1.1-1)	842	1.05 (1.1-1)	1168	1.06 (1.2-1)	<0.001
Glu-R (mg/dL)	837	84 (101-76)	3918	91 (116-79)	816	126 (189-96)	1071	145 (220-106)	<0.001
HbA1c (%)	284	5.5 (5.9-5.3)	2008	5.6 (6.1-5.3)	689	5.9 (6.9-5.5)	895	6.1 (7.4-5.6)	<0.001

When compared to other severity groups, LFT in the severe patients showed elevated TBIL, DBIL, and liver enzymes (SGPT/ALT, SGOT/AST, and ALP) but lower IBIL. Except for globulin, plasma proteins in the severe patients had lower levels of total protein, albumin, and A/G ratio. Patients with severe COVID-19 had the highest urea, creatinine, and uric acid values among all the severity groups. Electrolyte levels such as sodium, calcium, and chloride were much lower in the severe patients than in the other severity groups, with the exception of potassium and phosphorus, which were highest in the severe patients (Table 4).

All immunological biomarkers (ferritin, LDH, CRP, procalcitonin, IL-6, and CRP/ALB), coagulation biomarkers (aPTT, D-dimer, fibrinogen, PT, and INR), and hyperglycemia biomarkers (random glucose and HbA1c) were significantly higher in the severe patients compared to the other severity groups (Table 4).

All the laboratory biomarkers were categorized taking account of all the optimal diagnostic cutoff of the severity groups (Table 5).

**Table 5:** Area under curve (AUC) under the receiver operating characteristic (ROC) curve and optimal cutoff values for the laboratory biomarkers of various COVID-19 severity groups Hb, hemoglobin; HCT, hematocrit; RBC, red blood cell; TLC, total leucocyte count; PLT, platelet; MCV, mean corpuscular volume; MCH, mean corpuscular hemoglobin; MCHC, mean corpuscular hemoglobin concentration; RDW: red cell distribution width; ANC, absolute neutrophil counts; ALC, absolute lymphocyte counts; AMC, absolute monocyte counts; AEC, absolute eosinophil counts; ABC, absolute basophil counts; NLR, neutrophil-lymphocyte ratio; PLR, platelet-to-lymphocyte ratio; LMR, lymphocyte-to-monocyte ratio; NMR, neutrophil-to-monocyte ratio; SII, systemic immune-inflammation index; PT, prothrombin time; INR, international normalized ratio; aPTT, activated partial thromboplastin time; TBIL, total bilirubin; DBIL, direct bilirubin; IBIL, indirect bilirubin; ALT/SGPT, alanine aminotransferase/serum glutamic pyruvic transaminase; AST/SGOT, aspartate aminotransferase/serum glutamic oxaloacetic transaminase; ALP, alkaline phosphatase; TP, total protein; ALB, albumin; A/G, albumin/globulin; CRP/ALB, C-reactive protein/albumin; Glu-R, serum random glucose; HbA1c, glycated hemoglobin; COVID-19, coronavirus disease 2019; LDH, lactate dehydrogenase

	Asymptomatic-Mild		Asymptomatic-Moderate		Asymptomatic-Severe		Mild-Moderate		Mild-Severe		Moderate-Severe		Non-severe-Severe	
	AUC; P Value	Cutoff	AUC; P Value	Cutoff	AUC; P Value	Cutoff	AUC; P Value	Cutoff	AUC; P Value	Cutoff	AUC; P Value	Cutoff	AUC; P Value	Cutoff
Age (Years)	0.55; <0.001	>40	0.72; <0.001	>40	0.79; <0.001	>42	0.68; <0.001	>39	0.75; <0.001	>43	0.59; <0.001	>49	0.73; <0.001	>42
Duration (Days)	0.57; <0.001	>9	0.59; <0.001	≤8	0.51; 0.43	≤8	0.55; <0.001	≤8	0.53; <0.001	≤13	0.56; <0.001	>11	0.53; <0.001	>13
Hb (g/dL)	0.54; <0.001	≤13.6	0.63; <0.001	≤13.6	0.64; <0.001	≤13.1	0.59; <0.001	≤14.2	0.61; <0.001	≤13	0.52; 0.16	≤11.8	0.6; <0.001	≤13
HCT (%)	0.55; <0.001	≤43.2	0.65; <0.001	≤42.2	0.65; <0.001	≤41.6	0.60; <0.001	≤42.91	0.6; <0.001	≤40.12	0.51; 0.33	≤36.3	0.6; <0.001	≤40.12
RBC (10^6^/µL)	0.53; <0.001	≤4.09	0.62; <0.001	≤4.65	0.63; <0.001	≤4.59	0.59; <0.001	≤4.7	0.6; <0.001	≤4.33	0.52; 0.09	≤4.09	0.6; <0.001	≤4.33
TLC (10^3^/µL)	0.56; <0.001	>5.38	0.62; <0.001	>8.41	0.74; <0.001	>8.02	0.66; <0.001	>7.23	0.77; <0.001	>7.73	0.61; <0.001	>7.95	0.74; <0.001	>7.73
PLT (10^3^/µL)	0.55; <0.001	>215	0.55; <0.001	>309	0.53; <0.001	>303	0.59; <0.001	>271	0.58; <0.001	>293	0.51; 0.38	>136	0.56; <0.001	>303
MCV (fL)	0.52; 0.07	≤92.8	0.53; 0.05	≤94.6	0.5; 0.8	≤101	0.51; 0.46	≤96.9	0.52; 0.1	≤95.7	0.52; 0.06	≤96.9	0.51; 0.13	≤95.7
MCH (pg)	0.51; 0.34	≤30.8	0.53; 0.02	≤30.9	0.52; 0.1	≤28.4	0.52; 0.03	≤30.06	0.51; 0.25	≤27.97	0.51; 0.35	≤30.5	0.51; 0.32	≤27.96
MCHC (g/dL)	0.52; 0.14	>31.90	0.51; 0.32	≤30.98	0.54; <0.001	≤30.75	0.53; 0.01	>31.41	0.56; <0.001	>31.37	0.53; <0.001	≤32.08	0.55; <0.001	≤31.52
RDW (%)	0.51; 0.52	≤14.3	0.5; 0.85	≤15.7	0.56; <0.001	≤15	0.50; 0.73	≤16	0.56; <0.001	≤14.8	0.56; <0.001	≤14.5	0.56; <0.001	≤14.8
Neutrophils (%)	0.56; <0.001	>61	0.87; <0.001	>64.6	0.93; <0.001	>69.2	0.82; <0.001	>64.6	0.89; <0.001	>70.5	0.66; <0.001	>82	0.86; <0.001	>71.7
Lymphocytes (%)	0.55; <0.001	≤25.5	0.86; <0.001	≤22.7	0.92; <0.001	≤20.6	0.82; <0.001	≤21.2	0.89; <0.001	≤19	0.66; <0.001	≤11.9	0.86; <0.001	≤17.4
Eosinophils (%)	0.63; <0.001	≤1.5	0.86; <0.001	≤0.6	0.92; <0.001	≤0.5	0.77; <0.001	≤0.6	0.85; <0.001	≤0.3	0.61; <0.001	≤0.1	0.82; <0.001	≤0.3
Monocytes (%)	0.54; <0.001	>7.4	0.62; <0.001	≤4.7	0.73; <0.001	≤5	0.64; <0.001	≤5.2	0.75; <0.001	≤5	0.61; <0.001	≤5	0.73; <0.001	≤5
Basophils (%)	0.57; <0.001	≤0.8	0.76; <0.001	≤0.5	0.79; <0.001	≤0.4	0.70; <0.001	≤0.4	0.74; <0.001	≤0.4	0.55; <0.001	≤0.3	0.72; <0.001	≤0.4
ANC (10^3^/µL)	0.53; <0.001	≤2.94	0.74; <0.001	>4.98	0.85; <0.001	>5.17	0.75; <0.001	>4.56	0.84; <0.001	>4.82	0.63; <0.001	>6.03	0.81; <0.001	>4.86
ALC (10^3^/µL)	0.61; <0.001	≤1.4	0.84; <0.001	≤1.40	0.89; <0.001	≤1.27	0.76; <0.001	≤1.26	0.83; <0.001	≤1.0	0.62; <0.001	≤0.9	0.81; <0.001	≤1.0
AEC (10^3^/µL)	0.65; <0.001	≤0.07	0.85; <0.001	≤0.03	0.9; <0.001	≤0.03	0.74; <0.001	≤0.03	0.81; <0.001	≤0.02	0.58; <0.001	≤0.01	0.79; <0.001	≤0.01
AMC (10^3^/µL)	0.53; <0.001	>0.27	0.52; 0.14	>0.54	0.54; <0.001	>0.53	0.54; <0.001	>0.54	0.56; <0.001	>0.51	0.52; 0.12	>0.44	0.55; <0.001	>0.51
ABC (10^3^/µL)	0.59; <0.001	≤0.049	0.7; <0.001	≤0.049	0.68; <0.001	≤0.049	0.61; <0.001	≤0.06	0.59; <0.001	≤0.039	0.52; 0.19	>0.062	0.59; <0.001	≤0.039
NLR (Ratio)	0.5; 0.78	≤1.35	0.87; <0.001	>3.05	0.93; <0.001	>3.25	0.82; <0.001	>3.15	0.89; <0.001	>3.89	0.66; <0.001	>6.80	0.86; <0.001	>3.89
PLR (Ratio)	0.51; 0.6	>203.1	0.79; <0.001	>182.50	0.84; <0.001	>203.06	0.75; <0.001	>195.64	0.81; <0.001	>198.56	0.58; <0.001	>252.67	0.78; <0.001	>207.91
LMR (Ratio)	0.53; 0.03	≤3.36	0.79; <0.001	≤3.24	0.85; <0.001	≤3.1	0.74; <0.001	≤2.83	0.81; <0.001	≤3.01	0.61; <0.001	≤2.10	0.79; <0.001	≤3.125
SII (Ratio)	0.52; 0.17	>322.72	0.82; <0.001	>632.76	0.88; <0.001	>811.49	0.79; <0.001	>642.43	0.86; <0.001	>810.33	0.62; <0.001	>1172.07	0.83; <0.001	>806.67
NMR (Ratio)	0.52; 0.05	>7.24	0.76; <0.001	>12.24	0.86; <0.001	>12.86	0.75; <0.001	>11.43	0.84; <0.001	>12.10	0.63; <0.001	>16.20	0.81; <0.001	>12.88
TBIL (mg/dL)	0.55; <0.001	≤0.42	0.61; <0.001	≤0.5	0.57; <0.001	≤0.5	0.55; <0.001	≤0.5	0.52; <0.001	≤0.5	0.53; <0.001	≤0.53	0.52; 0.04	≤0.5
DBIL (mg/dL)	0.52; 0.09	>0.11	0.51; 0.63	>0.19	0.58; <0.001	>0.19	0.52; 0.03	>0.19	0.6; <0.001	>0.19	0.58; <0.001	>0.22	0.59; <0.001	>0.19
IBIL (mg/dL)	0.57; <0.001	≤0.34	0.67; <0.001	≤0.32	0.67; <0.001	≤0.3	0.60; <0.001	≤0.32	0.6; <0.001	≤0.3	0.51; 0.47	≤0.18	0.6; <0.001	≤0.3
SGPT/ALT (U/L)	0.54; <0.001	>25.1	0.63; <0.001	>73.6	0.63; <0.001	>61.7	0.59; <0.001	>55.3	0.6; <0.001	>46.9	0.5; 0.74	>47	0.59; <0.001	>46.9
SGOT/AST (U/L)	0.56; <0.001	>38.1	0.69; <0.001	>48.7	0.74; <0.001	>44.9	0.63; <0.001	>47.2	0.69; <0.001	>42.9	0.56; <0.001	>57.2	0.68; <0.001	>44.9
TP (g/dL)	0.57; <0.001	≤6.85	0.74; <0.001	≤6.61	0.8; <0.001	≤6.6	0.67; <0.001	≤6.61	0.74; <0.001	≤6.45	0.58; <0.001	≤6.46	0.73; <0.001	≤6.45
ALP (IU)	0.54; <0.001	>85	0.54; <0.001	>66	0.52; 0.15	>127	0.51; 0.33	>65	0.55; <0.001	>120	0.56; <0.001	>85	0.55; <0.001	>128
Globulin (g/dL)	0.51; 0.39	≤2.63	0.51; 0.49	≤2.1	0.52; 0.14	≤2.49	0.50; 0.94	≤2.2	0.53; <0.001	≤2.99	0.53; <0.001	≤2.57	0.53; <0.001	>2.59
A/G Ratio	0.54; <0.001	≤1.72	0.67; <0.001	≤1.60	0.77; <0.001	≤1.60	0.64; <0.001	≤1.56	0.74; <0.001	≤1.5	0.61; <0.001	≤1.5	0.73; <0.001	≤1.5
Albumin (g/dL)	0.59; <0.001	≤4.2	0.82; <0.001	≤4.1	0.89; <0.001	≤4	0.75; <0.001	≤>4	0.84; <0.001	≤4	0.65; <0.001	≤3.6	0.82; <0.001	≤4
Urea (mg/dL)	0.54; <0.001	>27.8	0.79; <0.001	>25.7	0.86; <0.001	>29	0.75; <0.001	>25.7	0.83; <0.001	>31	0.64; <0.001	>40.7	0.81; <0.001	>31
Creatinine (mg/dL)	0.52; 0.06	>0.78	0.56; <0.001	>0.93	0.61; <0.001	>0.99	0.54; <0.001	>0.96	0.59; <0.001	>0.96	0.56; <0.001	>0.97	0.59; <0.001	>0.97
Calcium (mg/dL)	0.6; <0.001	≤9	0.76; <0.001	≤8.7	0.84; <0.001	≤8.71	0.67; <0.001	≤8.7	0.77; <0.001	≤8.6	0.63; <0.001	≤8.36	0.76; <0.001	≤8.71
Phosphorus (mg/dL)	0.59; <0.001	≤3	0.59; <0.001	≤3.01	0.55; <0.001	≤2.8	0.51; 0.36	≤2.62	0.53; <0.001	≤3.45	0.54; <0.001	≤3.45	0.52; 0.04	≤3.45
Sodium (mmol/L)	0.55; <0.001	≤138	0.7; <0.001	≤138	0.67; <0.001	≤137	0.65; <0.001	≤138	0.63; <0.001	≤137	0.5; 0.72	≤142	0.62; <0.001	≤137
Potassium (mmol/L)	0.5; 0.95	>3.7	0.59; <0.001	>4.5	0.64; <0.001	>4.5	0.59; <0.001	>4.5	0.64; <0.001	>4.5	0.55; <0.001	>4.7	0.62; <0.001	>4.5
Chloride (mmol/L)	0.54; <0.001	≤102	0.71; <0.001	≤102	0.69; <0.001	≤102	0.67; <0.001	≤102	0.66; <0.001	≤102	0.51; 0.69	≤97	0.64; <0.001	≤102
Uric Acid (mg/dL)	0.53; 0.01	≤5.26	0.59; <0.001	≤4.67	0.52; 0.18	≤7.18	0.57; <0.001	≤4.61	0.5; 0.73	≤7.47	0.56; <0.001	≤5.75	0.51; 0.38	>6.91
Ferritin (ng/mL)	0.6; <0.001	>190.9	0.82; <0.001	>250.2	0.89; <0.001	>314	0.73; <0.001	>249.7	0.82; <0.001	>307.4	0.63; <0.001	>445.4	0.79; <0.001	>422.5
LDH (U/L)	0.62; <0.001	>239	0.84; <0.001	>264	0.92; <0.001	>314	0.75; <0.001	>310	0.86; <0.001	>24	0.68; <0.001	>479	0.83; <0.001	>324
CRP (U/L)	0.65; <0.001	>0.26	0.91; <0.001	>0.894	0.95; <0.001	>1.25	0.81; <0.001	>1.91	0.9; <0.001	>2.59	0.7; <0.001	>6.40	0.87; <0.001	>2.59
Procalcitonin (ng/mL)	0.6; <0.001	>0.02	0.73; <0.001	>0.02	0.82; <0.001	>0.03	0.64; <0.001	>0.03	0.75; <0.001	>0.04	0.63; <0.001	>0.1	0.73; <0.001	>0.04
IL-6 (pg/mL)	0.67; <0.001	>4	0.78; <0.001	>3.7	0.86; <0.001	>6.2	0.63; <0.001	>4.5	0.75; <0.001	>10.1	0.65; <0.001	>28.6	0.73; <0.001	>10.4
CRP/ALB (Ratio)	0.59; <0.001	>0.06	0.91; <0.001	>0.22	0.96; <0.001	>0.31	0.82; <0.001	>0.43	0.9; <0.001	>0.60	0.71; <0.001	>1.78	0.87; <0.001	>0.62
aPTT (Seconds)	0.51; 0.34	≤32.1	0.55; <0.001	≤31.2	0.51; 0.75	≤38.8	0.54; <0.001	≤30.8	0.51; 0.66	≤27.6	0.54; 0.002	≤35.2	0.51; 0.32	>35.5
D-Dimer (ng/mL)	0.61; <0.001	>86	0.83; <0.001	>113	0.9; <0.001	>176	0.74; <0.001	>148	0.84; <0.001	>185	0.67; <0.001	>349	0.82; <0.001	>199
Fibrinogen (mg/dL)	0.61; <0.001	>322	0.83; <0.001	>362	0.84; <0.001	>372	0.75; <0.001	>380	0.77; <0.001	>397	0.56; <0.001	>424	0.74; <0.001	>397
PT (Seconds)	0.52; 0.15	>11.9	0.51; 0.72	>13.2	0.55; <0.001	>13.2	0.52; 0.1	>13.2	0.57; <0.001	>12.9	0.55; <0.001	>13.9	0.56; <0.001	>12.9
INR (Ratio)	0.53; 0.05	≤1.02	0.51; 0.66	≤0.97	0.54; <0.001	≤1.15	0.51; 0.23	≤1.13	0.56; <0.001	≤1.12	0.54; <0.001	≤1.12	0.56; <0.001	≤1.12
Glu-R (mg/dL)	0.59; <0.001	>86	0.79; <0.001	>97	0.84; <0.001	>100	0.72; <0.001	>99	0.78; <0.001	>109	0.58; <0.001	>113	0.76; <0.001	>109
HbA1c (%)	0.55; <0.001	>5.87	0.67; <0.001	>5.78	0.71; <0.001	>5.96	0.61; <0.001	>5.57	0.66; <0.001	>5.99	0.56; <0.001	>6	0.64; <0.001	>5.99

Univariate analysis for crude odds ratio (OR) was conducted. Neutrophil percentage, lymphocyte (percentage and absolute), eosinophils (percentage and absolute), basophil percentage, TBIL, total protein, A/G ratio, albumin, calcium, phosphorus, sodium, ferritin, LDH, IL-6, CRP/ALB, D-dimer, and fibrinogen were significantly associated with increased severity in COVID-19 patients (Table 6). Therefore, these variables were included in a multinomial logistic regression model.

**Table 6:** Crude odd ratio (COR) of COVID-19 Severity. Reference: age, ≤39 years; duration, ≤8 days; Hb, ≥14.3 g/dL; HCT, ≥43.2%; RBC, ≥4.7×106/µL; TLC, ≤5.38×103/µL; PLT, ≥309×103/µL; MCV, ≤92.7 fL; MCH, ≥30.9 pg; MCHC, ≥31.9 g/dL; RDW, ≤14.2%; neutrophils, ≤61%; lymphocytes, ≥25.5%; eosinophils, ≥1.5%; monocytes, ≥7.4%; basophils, ≥0.8%; ANC, ≤2.94×103/µL; ALC, ≥1.39×103/µL; AEC, ≥0.07×103/µL; AMC, ≤0.27×103/µL; ABC, ≤0.048×103/µL; NLR, ≤1.34 ratio; PLR, ≤203.05 ratio; LMR, ≥3.37 ratio; SII, ≤322.71 ratio; NMR, ≤7.23 ratio; TBIL, ≤0.41 mg/dL; DBIL, ≤0.11 mg/dL; IBIL, ≥0.35 mg/dL; SGPT/ALT, ≤25.1 U/L; SGOT/AST, ≤38.1 U/L; TP, ≥6.85 g/dL; ALP, ≤85 IU; globulin, ≤2.1 g/dL; A/G ratio, ≥1.72 ratio; albumin, ≥4.3 g/dL; urea, ≤27.8 mg/dL; creatinine, ≤0.77 mg/dL; calcium, ≥9 mg/dL; phosphorus, ≤2.8 mg/dL; sodium, ≤135 mmol/L; potassium, ≤3.7 mmol/L; chloride, ≥102 mmol/L; uric acid, ≤5.25 mg/dL; ferritin, ≤190.8 ng/mL; LDH, ≤238 U/L; CRP, ≤0.26 mg/dL; procalcitonin, ≤0.019 ng/mL; IL-6, ≤4 pg/mL; CRP/ALB, ≤0.059 ratio; aPTT, ≤32.1 seconds; D-dimer, ≤86 ng/mL; fibrinogen, ≤322 mg/dL; PT, ≤11.8 seconds; INR, ≤1.01 ratio; Glu-R, ≤86 mg/dL; HbA1c, ≤5.86% N, number of patients; Hb, hemoglobin; HCT, hematocrit; RBC, red blood cell; TLC, total leucocyte count; PLT, platelet; MCV, mean corpuscular volume; MCH, mean corpuscular hemoglobin; MCHC, mean corpuscular hemoglobin concentration; RDW: red cell distribution width; ANC, absolute neutrophil counts; ALC, absolute lymphocyte counts; AMC, absolute monocyte counts; AEC, absolute eosinophil counts; ABC, absolute basophil counts; NLR, neutrophil-lymphocyte ratio; PLR, platelet-to-lymphocyte ratio; LMR, lymphocyte-to-monocyte ratio; NMR, neutrophil-to-monocyte ratio; SII, systemic immune-inflammation index; PT, prothrombin time; INR, international normalized ratio; aPTT, activated partial thromboplastin time; TBIL, total bilirubin; DBIL, direct bilirubin; IBIL, indirect bilirubin; ALT/SGPT, alanine aminotransferase/serum glutamic pyruvic transaminase; AST/SGOT, aspartate aminotransferase/serum glutamic oxaloacetic transaminase; ALP, alkaline phosphatase; TP, total protein; ALB, albumin; A/G, albumin/globulin; CRP/ALB, C-reactive protein/albumin; Glu-R, serum random glucose; HbA1c, glycated hemoglobin; COVID-19, coronavirus disease 2019; LDH, lactate dehydrogenase

	Asymptomatic (Number)	Mild (Number)		Moderate (Number)	Severe (Number)	P Value	COR (95% CI); P Value		
Age (Years)	≤39 (3526)	40-43 (606)		44-49 (802)	≥50 (2482)		40-43	44-49	≥50
Asymptomatic (n: 954)	600	77		77	200		Reference	Reference	Reference
Mild (n: 4295)	2426	357		449	1063	<0.001	1.1 (0.9-1.5); 0.31	1.4 (1.1-1.9); 0.01	1.3 (1.1-1.6); <0.001
Moderate (n: 905)	256	82		130	437		2.5 (1.8-3.5); <0.001	4 (2.9-5.4); <0.001	5.1 (4.1-6.4); <0.001
Severe (n: 1262)	244	90		146	782		2.9 (2-4); <0.001	4.7 (3.4-6.4); <0.001	9.6 (7.8-11.9); <0.001
Duration (Days)	≤8 (2522)	8.1-10 (2079)		11-12.9 (996)	≥13 (1819)		8.1-10	11-12.9	≥13
Asymptomatic (n: 954)	243	304		171	236		Reference	Reference	Reference
Mild (n: 4295)	1391	1378		591	935	<0.001	0.8 (0.7-1.0); 0.01	0.6 (0.5-0.8); <0.001	0.7 (0.6-0.8); <0.001
Moderate (n: 905)	420	175		96	214		0.3 (0.3-0.4); <0.001	0.3 (0.2-0.4); <0.001	0.5 (0.4-0.7); <0.001
Severe (n: 1262)	468	222		138	434		0.4 (0.3-0.5); <0.001	0.4 (0.3-0.6); <0.001	1.0 (0.8-1.2); 0.68
Hematological Biomarkers									
Hemoglobin (g/dL)	≥14.3 (2357)	14.2-13.7 (891)		13.6-11.9 (2368)	≤11.8 (1745)		14.2-13.7	13.6-11.9	≤11.8
Asymptomatic (n: 945)	377	134		276	158		Reference	Reference	Reference
Mild (n: 4265)	1506	516		1362	881	<0.001	1 (0.8-1.2); 0.75	1.2 (1-1.5); 0.02	1.4 (1.1-1.7); <0.001
Moderate (n: 895)	187	115		328	265		1.7 (1.3-2.3); <0.001	2.4 (1.9-3); <0.001	3.4 (2.6-4.4); <0.001
Severe (n: 1256)	287	126		402	441		1.2 (0.9-1.6); 0.15	1.9 (1.5-2.4); <0.001	3.7 (2.9-4.7); <0.001
Hematocrit (%)	≥43.2 (3115)	43.21-42.9 (163)		43.00-36.4 (2883)	≤36.3 (1172)		43.21-42.9	43.00-36.4	≤36.3
Asymptomatic (n: 940)	503	15		339	83		Reference	Reference	Reference
Mild (n: 4253)	1923	117		1636	577	<0.001	2 (1.2-3.5); 0.01	1.3 (1.1-1.5); <0.001	1.8 (1.4-2.3); <0.001
Moderate (n: 888)	285	11		409	183		1.3 (0.6-2.9); 0.52	2.1 (1.7-2.6); <0.001	3.9 (2.9-5.2); <0.001
Severe (n: 1252)	404	20		499	329		1.7 (0.8-3.3); 0.15	1.8 (1.5-2.2); <0.001	4.9 (3.8-6.5); <0.001
RBC Count (10^6^/µL)	≥4.7 (3538)	4.69-4.30 (1767)	4.29-4.10 (693)		≤4.09 (1363)		4.69-4.30	4.29-4.10	≤4.09
Asymptomatic (n: 945)	519	218		93	115		Reference	Reference	Reference
Mild (n: 4265)	2208	1015		348	694		1.1 (0.9-1.3); 0.31	0.9 (0.7-1.1); 0.31	1.4 (1.1-1.8); <0.001
Moderate (n: 895)	344	235		120	196	<0.001	1.6 (1.3-2); <0.001	1.9 (1.4-2.6); <0.001	2.6 (2-3.4); <0.001
Severe (n: 1256)	467	299		132	358		1.5 (1.2-1.9); <0.001	1.6 (1.2-2.1); <0.001	3.5 (2.7-4.4); <0.001
MCV (fL)	≤92.7 (4814)	92.8-94.5 (765)		94.6-100 (1193)	≥101 (589)		92.8-94.5	94.6-100	≥101
Asymptomatic (n: 945)	587	113		143	102		Reference	Reference	Reference
Mild (n: 4265)	2829	431		690	315	0.001	0.9 (0.7-1.1); 0.3	0.9 (0.8-1.1); 0.38	0.6 (0.5-0.8); <0.001
Moderate (n: 895)	602	102		130	61		0.9 (0.7-1.3); 0.65	0.9 (0.7-1.1); 0.25	0.6 (0.4-0.8); <0.001
Severe (n: 1256)	796	119		230	111		0.8 (0.6-1.1); 0.2	1.1 (0.8-1.3); 0.58	0.8 (0.6-1.1); 0.12
MCH (pg)	≥30.9 (1284)	30.8-30. 1 (769)		30.0-28.1 (2603)	≤28 (2705)		30.8-30.1	30.0-28.1	≤28
Asymptomatic (n: 945)	189	94		319	343		Reference	Reference	Reference
Mild (n: 4265)	748	461		1527	1529	0.031	1.2 (0.9-1.6); 0.12	1.2 (1-1.5); 0.06	1.1 (0.9-1.4); 0.24
Moderate (n: 895)	123	90		335	347		1.5 (1-2.1); 0.04	1.6 (1.2-2.1); <0.001	1.6 (1.2-2); <0.001
Severe (n: 1256)	224	124		422	486		1.1 (0.8-1.5); 0.53	1.1 (0.9-1.4); 0.37	1.2 (0.9-1.5); 0.14
MCHC (g/dL)	≥31.9 (2987)	31.8-30.98 (2149)		30.97-30.74 (384)	≤30.75 (1841)		31.8-30.98	30.97-30.74	≤30.75
Asymptomatic (n: 945)	372	302		47	224		Reference	Reference	Reference
Mild (n: 4265)	1815	1254		212	984	<0.001	0.9 (0.7-1); 0.06	0.9 (0.7-1.3); 0.65	0.9 (0.8-1.1); 0.26
Moderate (n: 895)	355	234		53	253		0.8 (0.6-1); 0.07	1.2 (0.8-1.8); 0.44	1.2 (0.9-1.5); 0.15
Severe (n: 1256)	445	359		72	380		1 (0.8-1.2); 0.95	1.3 (0.9-1.9); 0.22	1.4 (1.1-1.8); <0.001
RDW (%)	≤14.2 (3902)	14.3-14.5 (627)		14.6-14.7 (385)	≥14.8 (2447)		14.3-14.5	14.6-14.7	≥14.8
Asymptomatic (n: 945)	490	89		65	301		Reference	Reference	Reference
Mild (n: 4265)	2333	368		224	1340	<0.001	0.9 (0.7-1.1); 0.27	0.7 (0.5-1); 0.03	0.9 (0.8-1.1); 0.41
Moderate (n: 895)	484	82		35	294		0.9 (0.7-1.3); 0.68	0.5 (0.4-0.8); 0.01	1 (0.8-1.2); 0.91
Severe (n: 1256)	595	88		61	512		0.8 (0.6-1.1); 0.21	0.8 (0.5-1.1); 0.17	1.4 (1.2-1.7); <0.001
PLT (10^3^/µL)	≥309 (1302)	308-272 (636)		271-137 (4253)	≤136 (1170)		308-272	271-137	≤136
Asymptomatic (n: 945)	164	107		543	131		Reference	Reference	Reference
Mild (n: 4265)	572	332		2639	722	<0.001	0.9 (0.7-1.2); 0.41	1.4 (1.1-1.7); <0.001	1.6 (1.2-2); <0.001
Moderate (n: 895)	231	98		456	110		0.7 (0.5-0.9); 0.01	0.6 (0.5-0.8); <0.001	0.6 (0.4-0.8); <0.001
Severe (n: 1256)	335	99		615	207		0.8 (0.6-1); 0.08	0.6 (0.4-0.7); <0.001	0.5 (0.3-0.6); <0.001
TLC (10^3^/µL)	≤5.38 (2910)	5.39-7.23 (2061)		7.24-8.40 (758)	≥8.41 (1632)		5.39-7.23	7.24-8.40	≥8.41
Asymptomatic (n: 945)	336	364		124	121		Reference	Reference	Reference
Mild (n: 4265)	2086	1273		410	496	<0.001	0.6 (0.5-0.7); <0.001	0.5 (0.4-0.7); <0.001	0.7 (0.5-0.8); <0.001
Moderate (n: 895)	260	199		99	337		0.7 (0.6-0.9); <0.001	1 (0.8-1.4); 0.84	3.6 (2.8-4.7); <0.001
Severe (n: 1256)	228	225		125	678		0.9 (0.7-1.2); 0.44	1.5 (1.1-2); 0.01	8.3 (6.4-10.7); <0.001
Neutrophils (%)	≤61 (3660)	61.1-64.6 (615)		64.7-80 (1635)	≥81 (1238)		61.1-64.6	64.7-80	≥81
Asymptomatic (n: 910)	688	84		137	1		Reference	Reference	Reference
Mild (n: 4153)	2700	444		834	175	<0.001	1.3 (1.1-1.7); 0.02	1.6 (1.3-1.9); <0.001	44.6 (6.2-318.9); <0.001
Moderate (n: 862)	157	50		335	320		2.6 (1.8-3.9); <0.001	10.7 (8.2-13.9); <0.001	1402 (195.4-10062.1); <0.001
Severe (n: 1223)	115	37		329	742		2.6 (1.7-4.1); <0.001	14.4 (10.9-19); <0.001	4439 (618.3-31869.5); <0.001
Lymphocytes (%)	≥25.5 (3838)	25.4-20.6 (899)		20.5-17.5 (465)	≤17.4 (2011)		25.4-20.6	20.5-17.5	≤17.4
Asymptomatic (n: 911)	700	122		46	43		Reference	Reference	Reference
Mild (n: 4172)	2824	612		256	480	<0.001	2.8 (2-3.8); <0.001	1.4 (1-1.9); 0.05	1.2 (1-1.5); 0.04
Moderate (n: 886)	185	100		92	509		3.1 (2.3-4.2); <0.001	7.6 (5.1-11.2); <0.001	44.8 (31.5-63.6); <0.001
Severe (n: 1244)	129	65		71	979		2.9 (2-4.1); <0.001	8.4 (5.5-12.7); <0.001	123.5 (86.3-176.8); <0.001
Eosinophils (%)	≥1.5 (2875)	1.4-0.6 (1570)		0.6-0.4 (451)	≤0.3 (2317)		1.4-0.6	0.6-0.4	≤0.3
Asymptomatic (n: 911)	614	217		33	47		Reference	Reference	Reference
Mild (n: 4172)	2006	1104		285	777	<0.001	1.6 (1.3-1.8); <0.001	2.6 (1.8-3.8); <0.001	5.1 (3.7-6.9); <0.001
Moderate (n: 886)	146	126		70	544		2.4 (1.8-3.2); <0.001	8.9 (5.7-14); <0.001	48.7 (34.4-69); <0.001
Severe (n: 1244)	109	123		63	949		3.2 (2.4-4.3); <0.001	10.8 (6.7-17.2); <0.001	113.7 (79.6-162.5); <0.001
Monocytes (%)	≥7.4 (2267)	7.3-5.2 (2742)		5.1-4.9 (394)	≤5 (1810)		7.3-5.2	5.1-4.9	≤5
Asymptomatic (n: 911)	285	414		58	154		Reference	Reference	Reference
Mild (n: 4172)	1580	1734		214	644	<0.001	0.8 (0.6-0.9); <0.001	0.7 (0.5-0.9); 0.01	0.8 (0.6-0.9); 0.01
Moderate (n: 886)	215	293		46	332		0.9 (0.7-1.2); 0.59	1.1 (0.7-1.6); 0.82	2.9 (2.2-3.7); <0.001
Severe (n: 1244)	187	301		76	680		1.1 (0.9-1.4); 0.4	2 (1.4-2.9); <0.001	6.7 (5.2-8.7); <0.001
Basophils (%)	≥0.8 (2829)	0.7-0.45 (1757)		0.44-0.3 (1625)	≤0.29 (1002)		0.7-0.45	0.44-0.3	≤0.29
Asymptomatic (n: 911)	523	224		125	39		Reference	Reference	Reference
Mild (n: 4172)	1900	1110		865	297	<0.001	1.4 (1.1-1.6); <0.001	1.9 (1.5-2.4); <0.001	2.1 (1.5-3); <0.001
Moderate (n: 886)	188	194		275	229		2.4 (1.9-3.1); <0.001	6.1 (4.7-8); <0.001	16.3 (11.2-23.8); <0.001
Severe (n: 1244)	218	229		360	437		2.5 (1.9-3.1); <0.001	6.9 (5.3-8.9); <0.001	26.9 (18.7-38.7); <0.001
ANC (10^3^/µL)	≤2.94 (2719)	2.95-4.77 (2239)		4.78-6.05 (700)	≥6.06 (1528)		2.95-4.77	4.78-6.05	≥6.06
Asymptomatic (n: 905)	391	368		91	55		Reference	Reference	Reference
Mild (n: 4158)	2032	1439		344	343	<0.001	0.8 (0.6-0.9); <0.001	0.7 (0.6-0.9); 0.01	1.2 (0.9-1.6); 0.24
Moderate (n: 884)	170	223		124	367		1.4 (1.1-1.8); 0.01	3.1 (2.3-4.3); <0.001	15.3 (11-21.5); <0.001
Severe (n: 1239)	126	209		141	763		1.8 (1.4-2.3); <0.001	4.8 (3.5-6.7); <0.001	43 (30.7-60.4); <0.001
ALC (10^3^/µL)	≥1.39 (3741)	1.38-1.13 (1015)		1.12-0.91 (771)	≤0.9 (1568)		1.38-1.13	1.12-0.91	≤0.9
Asymptomatic (n: 904)	728	92		53	31		Reference	Reference	Reference
Mild (n: 4101)	2608	642		419	432	<0.001	1.9 (1.5-2.5); <0.001	2.2 (1.6-3); <0.001	3.9 (2.7-5.7); <0.001
Moderate (n: 866)	216	148		145	357		5.4 (4-7.3); <0.001	9.2 (6.5-13.1); <0.001	38.8 (26.1-57.7); <0.001
Severe (n: 1224)	189	133		154	748		5.6 (4.1-7.6); <0.001	11.2 (7.9-15.9); <0.001	92.9 (62.7-137.7); <0.001
AEC (10^3^/µL)	≥0.07 (3186)	0.06-0.04 (621)		0.03-0.02 (467)	≤0.01 (2939)		0.06-0.04	0.03-0.02	≤0.01
Asymptomatic (n: 911)	667	72		32	140		Reference	Reference	Reference
Mild (n: 4172)	2172	446		271	1283	<0.001	1.9 (1.5-2.5); <0.001	2.6 (1.8-3.8); <0.001	2.8 (2.3-3.4); <0.001
Moderate (n: 886)	181	47		75	583		2.4 (1.6-3.6); <0.001	8.6 (5.5-13.5); <0.001	15.3 (12-19.6); <0.001
Severe (n: 1244)	166	56		89	933		3.1 (2.1-4.6); <0.001	11.2 (7.2-17.3); <0.001	26.8 (20.9-34.2); <0.001
AMC (10^3^/µL)	≤0.27 (1678)	0.28-0.48 (3593)		0.48-0.50 (224)	≥0.51 (1718)		0.28-0.48	0.48-0.50	≥0.51
Asymptomatic (n: 911)	188	493		34	196		Reference	Reference	Reference
Mild (n: 4172)	966	2266		137	803	<0.001	0.9 (0.7-1.1); 0.24	0.8 (0.5-1.2); 0.24	0.8 (0.6-1); 0.04
Moderate (n: 886)	221	362		22	281		0.6 (0.5-0.8); <0.001	0.6 (0.3-1); 0.04	1.2 (0.9-1.6); 0.14
Severe (n: 1244)	303	472		31	438		0.6 (0.5-0.7); <0.001	0.6 (0.3-1); 0.03	1.4 (1.1-1.8); 0.01
ABC (10^3^/µL)	≤0.048 (4473)	0.049-0.050 (116)		0.051-0.061 (503)	≥0.062 (2121)		0.049-0.050	0.051-0.061	≥0.062
Asymptomatic (n: 911)	406	17		72	416		Reference	Reference	Reference
Mild (n: 4172)	2503	63		301	1305	<0.001	0.6 (0.3-1); 0.07	0.7 (0.5-0.9); 0.01	0.5 (0.4-0.6); <0.001
Moderate (n: 886)	680	12		62	132		0.4 (0.2-0.9); 0.02	0.5 (0.4-0.7); <0.001	0.2 (0.2-0.2); <0.001
Severe (n: 1244)	884	24		68	268		0.6 (0.3-1.2); 0.18	0.4 (0.3-0.6); <0.001	0.3 (0.2-0.4); <0.001
NLR (Ratio)	≤1.34 (49)	1.35-3.10 (136)		3.11-6.80 (276)	≥6.81 (783)		1.35-3.10	3.11-6.80	≥6.81
Asymptomatic (n: 911)	290	532		86	3		Reference	Reference	Reference
Mild (n: 4172)	1264	2150		558	200	<0.001	0.9 (0.8-1.1); 0.35	1.5 (1.1-1.9); <0.001	15.3 (4.9-48.2); <0.001
Moderate (n: 886)	45	224		271	346		2.7 (1.9-3.9); <0.001	20.3 (13.7-30.2); <0.001	743.3 (228.6-2416.6); <0.001
Severe (n: 1244)	49	136		276	783		1.5 (1.1-2.2); 0.02	19 (12.9-28); <0.001	1545 (477.8-4994.3); <0.001
PLR (Ratio)	≤203.05 (5178)	203.06-218.06 (215)		218.07-252.67 (353)	≥252.68 (1467)		203.06-218.06	218.07-252.67	≥252.68
Asymptomatic (n: 911)	828	21		26	36		Reference	Reference	Reference
Mild (n: 4172)	3468	126		164	414	<0.001	1.4 (0.9-2.3); 0.13	1.5 (1-2.3); 0.06	2.7 (1.9-3.9); <0.001
Moderate (n: 886)	413	29		73	371		2.8 (1.6-4.9); <0.001	5.6 (3.5-8.9); <0.001	20.7 (14.4-29.7); <0.001
Severe (n: 1244)	469	39		90	646		3.3 (1.9-5.6); <0.001	6.1 (3.9-9.6); <0.001	31.7 (22.2-45.1); <0.001
LMR (Ratio)	≥3.37 (4311)	3.36-3.03 (385)		3.02-2.11 (1028)	≤2.10 (1489)		3.36-3.03	3.02-2.11	≤2.10
Asymptomatic (n: 911)	748	47		72	44		Reference	Reference	Reference
Mild (n: 4172)	2953	240		591	388	<0.001	1.3 (0.9-1.8); 0.12	2.1 (1.6-2.7); <0.001	2.2 (1.6-3.1); <0.001
Moderate (n: 886)	312	52		170	352		2.7 (1.8-4); <0.001	5.7 (4.2-7.7); <0.001	19.2 (13.7-26.9); <0.001
Severe (n: 1244)	298	46		195	705		2.5 (1.6-3.8); <0.001	6.8 (5-9.2); <0.001	40.2 (28.8-56.1); <0.001
SII (Ratio)	≤322.71 (2359)	322.72-931.28 (2821)		931.29-1172.07 (321)	≥1172.08 (1712)		322.72-931.28	931.29-1172.07	≥1172.08
Asymptomatic (n: 911)	374	468		29	40		Reference	Reference	Reference
Mild (n: 4172)	1788	1840		153	391	<0.001	0.8 (0.7-1); 0.01	1.1 (0.7-1.7); 0.64	2 (1.5-2.9); <0.001
Moderate (n: 886)	98	292		64	432		2.4 (1.8-3.1); <0.001	8.4 (5.2-13.8); <0.001	41.2 (27.8-61.1); <0.001
Severe (n: 1244)	99	221		75	849		1.8 (1.4-2.3); <0.001	9.8 (6-15.8); <0.001	80.2 (54.5-118.1); <0.001
NMR (Ratio)	≤7.23 (2153)	7.24-11.83 (2678)		11.84-16.20 (1140)	≥16.21 (1242)		7.24-11.83	11.84-16.20	≥16.21
Asymptomatic (n: 911)	324	441		121	25		Reference	Reference	Reference
Mild (n: 4172)	1612	1768		526	266	<0.001	0.8 (0.7-0.9); 0.01	0.9 (0.7-1.1); 0.25	2.1 (1.4-3.3); <0.001
Moderate (n: 886)	127	248		226	285		1.4 (1.1-1.9); 0.01	4.8 (3.5-6.4); <0.001	29.1 (18.4-46); <0.001
Severe (n: 1244)	90	221		267	666		1.8 (1.4-2.4); <0.001	7.9 (5.8-10.9); <0.001	95.9 (60.4-152.3); <0.001
Biochemical Biomarkers									
TBIL (mg/dL)	≤0.41 (390)	0.42-0.50 (206)		0.51-0.53 (33)	≥0.54 (630)		0.42-0.50	0.51-0.53	≥0.54
Asymptomatic (n: 951)	191	148		52	560		Reference	Reference	Reference
Mild (n: 4277)	1168	656		216	2237	<0.001	0.65 (0.5-0.8); <0.001	0.68 (0.5-1.0); <0.001	0.73 (0.6-0.9); <0.001
Moderate (n:)	309	165		37	389		0.43 (0.3-0.5); <0.001	0.44 (0.3-0.7); <0.001	0.69 (0.5-0.9); <0.001
Severe (n: 1259)	390	206		33	630		0.55 (0.4-0.7); <0.001	0.31 (0.2-0.5); <0.001	0.68 (0.5-0.9); <0.001
DBIL (mg/dL)	≤0.11 (950)	0.12-0.1 9(2411)		0.20-0.22 (1081)	≥0.23 (2949)		0.12-0.19	0.20-0.22	≥0.23
Asymptomatic (n: 951)	91	376		109	375		Reference	Reference	Reference
Mild (n: 4280)	594	1502		593	1591	<0.001	0.65 (0.5-0.8); <0.001	0.83 (0.6-1.1); 0.24	0.61 (0.5-0.8); <0.001
Moderate (n: 900)	126	260		165	349		0.67 (0.5-0.9); <0.001	1.09 (0.8-1.6); 0.63	0.50 (0.4-0.7); <0.001
Severe (n: 1260)	139	273		214	634		1.11 (0.8-1.5); 0.50	1.29 (0.9-1.8); 0.16	0.48 (0.4-0.6); <0.001
IBIL (mg/dL)	≥0.35 (3637)	0.34-0.32 (484)		0.31-0.19 (2468)	≤0.18 (798)		0.34-0.32	0.31-0.19	≤0.18
Asymptomatic (n: 951)	587	63		258	43		Reference	Reference	Reference
Mild (n: 4277)	2227	307		1323	420	<0.001	2.58 (1.9-3.6); <0.001	1.35 (1.2-1.6); <0.001	1.28 (1.0-1.7); 0.09
Moderate (n: 900)	336	54		394	116		4.71 (3.2-6.9); <0.001	2.67 (2.2-3.3); <0.001	1.50 (1.0-2.2); <0.001
Severe (n: 1259)	487	60		493	219		6.14 (4.3-8.7); <0.001	2.30 (1.9-2.8); <0.001	1.15 (0.8-1.7); 0.47
SGPT/ALT (U/L)	≤25.1 (1995)	25.2-64.5 (3502)		64.6-73.5 (374)	≥73.6 (1519)		25.2-64.5	64.6-73.5	≥73.6
Asymptomatic (n: 951)	335	450		49	117		Reference	Reference	Reference
Mild (n: 4280)	1225	2090		204	761	<0.001	1.78(1.4-2.2);<0.001	1.14(0.8-1.6);0.45	1.27(1.1-1.5);<0.001
Moderate (n: 899)	182	403		45	269		4.23(3.2-5.6);<0.001	1.69(1.1-2.6);<0.001	1.65(1.3-2.1);<0.001
Severe (n: 1260)	253	559		76	372		4.21(3.2-5.5);<0.001	2.05(1.4-3.0);<0.001	1.65(1.3-2.0);<0.001
SGOT/AST (U/L)	≤38.1 (3998)	38.1-48.3 (1118)		48.4-57.1 (595)	≥57.2 (1680)		38.1-48.3	48.4-57.1	≥57.2
Asymptomatic (n: 951)	662	131		64	94		Reference	Reference	Reference
Mild (n: 4280)	2576	676		317	711	<0.001	1.94 (1.5-2.5); <0.001	1.27 (1.0-1.7); 0.09	1.33 (1.1-1.6); <0.001
Moderate (n: 900)	364	123		100	313		6.06 (4.7-7.9); <0.001	2.84 (2.0-4.0); <0.001	1.71 (1.3-2.3); <0.001
Severe (n: 1260)	396	188		114	562		10.00 (7.8-12.9); <0.001	2.98 (2.1-4.1); <0.001	2.40 (1.9-3.1); <0.001
TP (g/dL)	≥6.85 (3089)	6.84-6.61 (1233)		6.60-6.45 (778)	≤6.44 (2291)		6.84-6.61	6.60-6.45	≤6.44
Asymptomatic (n: 951)	564	170		77	140		Reference	Reference	Reference
Mild (n: 4280)	2046	809		465	960	<0.001	1.89 (1.5-2.3); <0.001	1.67 (1.3-2.2); <0.001	1.31 (1.1-1.6); <0.001
Moderate (n: 900)	240	130		104	426		7.15 (5.6-9.1); <0.001	3.17 (2.3-4.4); <0.001	1.80 (1.4-2.4); <0.001
Severe (n: 1260)	239	124		132	765		12.90 (10.2-16.3); <0.001	4.05 (2.9-5.6); <0.001	1.72 (1.3-2.3); <0.001
ALP (IU)	≤85 (4002)	86-110.7 (1813)		110.8-128 (640)	≥129 (935)		86-110.7	110.8-128	≥129
Asymptomatic (n: 951)	480	264		85	122		Reference	Reference	Reference
Mild (n: 4280)	2396	1100		386	398	<0.001	0.7 (0.5-0.8); <0.001	0.9 (0.7-1.2); 0.47	0.8 (0.7-1.0); <0.001
Moderate (n: 899)	518	179		65	137		1.0 (0.8-1.4); 0.78	0.7 (0.5-1.0); 0.05	0.6 (0.5-0.8); <0.001
Severe (n: 1260)	608	270		104	278		1.8 (1.4-2.3); <0.001	1.0 (0.7-1.3); 0.83	0.8 (0.7-1.0); <0.001
Globulin (g/dL)	≤2.1 (1044)	2.2-2.68 (3581)		2.69-2.99 (1674)	≥3 (1092)		2.2-2.68	2.69-2.99	≥3
Asymptomatic (n: 951)	136	457		215	143		Reference	Reference	Reference
Mild (n: 4280)	560	2178		997	545	<0.001	0.93 (0.7-1.2); 0.56	1.13 (0.9-1.4); 0.33	1.16 (0.9-1.4); 0.18
Moderate (n: 900)	151	408		182	159		1.00 (0.7-1.4); 0.99	0.76 (0.6-1.0); 0.08	0.80 (0.6-1.1); 0.11
Severe (n: 1260)	197	538		280	245		1.18 (0.9-1.6); 0.27	0.90 (0.7-1.2); 0.46	0.81 (0.6-1.0); 0.11
A/G Ratio	≥1.72 (2696)	1.71-1.60 (1114)		1.61-1.50 (1117)	≤1.49 (2464)		1.71-1.60	1.61-1.50	≤1.49
Asymptomatic (n: 951)	465	140		129	217		Reference	Reference	Reference
Mild (n: 4280)	1835	723		642	1080	<0.001	1.3 (1.1-1.5); <0.001	1.3 (1.0-1.6); <0.001	1.3 (1.1-1.6); <0.001
Moderate (n: 900)	227	117		157	399		3.8 (3.0-4.7); <0.001	2.5 (1.9-3.3); <0.001	1.7 (1.3-2.3); <0.001
Severe (n: 1260)	169	134		189	768		9.7 (7.7-12.3); <0.001	4.0 (3.0-5.4); <0.001	2.6 (2.0-3.5); <0.001
Albumin (g/dL)	≥4.3 (3211)	4.2-4 (1836)		3.9-3.6 (1346)	≤3.5 (998)		4.2-4	3.9-3.6	≤3.5
Asymptomatic (n: 951)	634	217		77	23		Reference	Reference	Reference
Mild (n: 4280)	2302	1169		555	254	<0.001	3.0 (2.0-4.7); <0.001	2.0 (1.5-2.6); <0.001	1.5 (1.3-1.8); <0.001
Moderate (n: 900)	159	237		317	187		32.4 (20.3-51.7); <0.001	16.4 (12.1-22.2); <0.001	4.4 (3.4-5.6); <0.001
Severe (n: 1260)	116	213		397	534		126.9 (80.0-201.4); <0.001	28.2 (20.6-38.6); <0.001	5.4 (4.1-7.1); <0.001
Urea (mg/dL)	≤27.8 (4887)	27.9-29 (143)		29.1-33.6 (531)	≥33.7 (1807)		27.9-29	29.1-33.6	≥33.7
Asymptomatic (n: 945)	806	19		59	61		Reference	Reference	Reference
Mild (n: 4268)	3401	91		280	496	<0.001	1.93 (1.5-2.5); <0.001	1.13 (0.8-1.5); 0.43	1.14 (0.7-1.9); 0.62
Moderate (n: 900)	368	16		93	423		15.19 (11.3-20.4); <0.001	3.45 (2.4-4.9); <0.001	1.84 (0.9-3.6); 0.08
Severe (n: 1255)	312	17		99	827		35.02 (26.2-46.9); <0.001	4.34 (3.1-6.1); <0.001	2.31 (1.2-4.5); <0.001
Creatinine (mg/dL)	≤0.77 (4013)	0.78-0.94 (2078)		0.95-0.97 (208)	≥0.98 (1076)		0.78-0.94	0.95-0.97	≥0.98
Asymptomatic (n: 947)	557	279		25	86		Reference	Reference	Reference
Mild (n: 4271)	2412	1294		124	441	<0.001	1.18 (0.9-1.5); 0.18	1.15 (0.7-1.8); 0.55	1.07 (0.9-1.3); 0.40
Moderate (n: 900)	478	231		28	163		2.21 (1.7-2.9); <0.001	1.31 (0.8-2.3); 0.35	0.97 (0.8-1.2); 0.74
Severe (n: 1257)	566	274		31	386		4.42 (3.4-5.7); <0.001	1.22 (0.7-2.1); 0.47	0.97 (0.8-1.2); 0.74
Calcium (mg/dL)	≥9 (3117)	8.9-8.7 (1425)		8.6-8.4 (1231)	≤8.3 (1598)		8.9-8.7	8.6-8.4	≤8.3
Asymptomatic (n: 947)	615	173		87	72		Reference	Reference	Reference
Mild (n: 4269)	2093	905		695	576	<0.001	2.4 (1.8-3.1); <0.001	2.4 (1.8-3.0); <0.001	1.5 (1.3-1.9); <0.001
Moderate (n: 900)	225	181		198	296		11.2 (8.3-15.2); <0.001	6.2 (4.6-8.4); <0.001	2.9 (2.2-3.7); <0.001
Severe (n: 1255)	184	166		251	654		30.4 (22.6-40.7); <0.001	9.6 (7.2-12.9); <0.001	3.2 (2.5-4.2); <0.001
Phosphorus (mg/dL)	≤2.8 (3100)	2.81-3.20 (1558)		3.21-3.60 (1229)	≥3.61 (1486)		2.81-3.20	3.21-3.60	≥3.61
Asymptomatic (n: 946)	298	211		173	264		Reference	Reference	Reference
Mild (n: 4271)	1881	920		713	757	<0.001	0.45 (0.4-0.5); <0.001	0.65 (0.5-0.8); <0.001	0.69 (0.6-0.8); <0.001
Moderate (n: 900)	403	189		149	159		0.45 (0.3-0.6); <0.001	0.64 (0.5-0.8); <0.001	0.66 (0.5-0.8); <0.001
Severe (n: 1256)	518	238		194	306		0.67 (0.5-0.8); <0.001	0.65 (0.5-0.8); <0.001	0.65 (0.5-0.8); <0.001
Sodium (mmol/L)	≤135 (974)	136-137 (932)		138-141 (3960)	≥142 (1509)		136-137	138-141	≥142
Asymptomatic (n: 947)	34	71		617	225		Reference	Reference	Reference
Mild (n: 4271)	354	472		2523	922	<0.001	0.39 (0.3-0.6); <0.001	0.39 (0.3-0.6); <0.001	0.64 (0.4-1.0); <0.001
Moderate (n: 900)	219	166		394	121		0.08 (0.1-0.1); <0.001	0.10 (0.1-0.1); <0.001	0.36 (0.2-0.6); <0.001
Severe (n: 1257)	367	223		426	241		0.10 (0.1-0.1); <0.001	0.06 (0.0-0.1); <0.001	0.29 (0.2-0.5); <0.001
Potassium (mmol/L)	≤3.7 (937)	3.8-4.4 (3363)		4.5-4.7 (1341)	>4.8 (1733)		3.8-4.4	4.5-4.7	>4.8
Asymptomatic (n: 946)	112	502		170	162		Reference	Reference	Reference
Mild (n: 4271)	602	2112		768	789	<0.001	0.91 (0.7-1.2); 0.46	0.84 (0.6-1.1); 0.19	0.78 (0.6-1.0); <0.001
Moderate (n: 900)	89	350		180	281		2.18 (1.6-3.1); <0.001	1.33 (0.9-1.9); 0.11	0.88 (0.6-1.2); 0.41
Severe (n: 1257)	134	399		223	501		2.59 (1.9-3.5); <0.001	1.10 (0.8-1.5); 0.57	0.66 (0.5-0.9); <0.001
Chloride (mmol/L)	≥102 (5508)	102.1-100.2 (457)		100.3-97.1 (844)	≤97 (566)		102.1-100.2	100.3-97.1	≤97
Asymptomatic (n: 947)	834	59		35	19		Reference	Reference	Reference
Mild (n: 4271)	3504	230		367	170	<0.001	2.1 (1.3-3.4); <0.001	2.5 (1.8-3.6); <0.001	0.9 (0.7-1.2); 0.62
Moderate (n: 900)	495	79		199	127		11.3 (6.9-18.5); <0.001	9.6 (6.6-14.0); <0.001	2.3 (1.6-3.2); <0.001
Severe (n: 1257)	675	89		243	250		16.3 (10.1-26.2); <0.001	8.6 (5.9-12.4); <0.001	1.9 (1.3-2.6); <0.001
Uric Acid (mg/dL)	≤5.25 (4331)	5.26-6.70 (2091)		6.71-7.80 (537)	≥7.81 (414)		5.26-6.70	6.71-7.80	≥7.81
Asymptomatic (n: 947)	510	324		82	31		Reference	Reference	Reference
Mild (n: 4269)	2510	1279		313	167	<0.001	1.10 (0.7-1.6); 0.65	0.78 (0.6-1.0); 0.06	0.80 (0.7-0.9); <0.001
Moderate (n: 900)	588	208		48	56		1.57 (1.0-2.5); 0.05	0.51 (0.3-0.7); <0.001	0.56 (0.5-0.7); <0.001
Severe (n: 1257)	723	280		94	160		3.64 (2.4-5.4); <0.001	0.81 (0.6-1.1); 0.19	0.61 (0.5-0.7); <0.001
Inflammatory Biomarkers									
Ferritin (ng/mL)	≤190.8 (2236)	190.9-355.5 (783)		355.6-445.3 (230)	≥445.4 (1323)		190.9-355.5	355.6-445.3	≥445.4
Asymptomatic (n: 460)	351	70		14	25		Reference	Reference	Reference
Mild (n: 2592)	1571	483		119	419	<0.001	3.75 (2.5-5.7); <0.001	1.90 (1.1-3.3); <0.001	1.54 (1.2-2.0); <0.001
Moderate (n: 629)	169	120		54	286		23.76 (15.2-37.2); <0.001	8.01 (4.3-14.8); <0.001	3.56 (2.5-5.0); <0.001
Severe (n: 891)	145	110		43	593		57.42 (36.8-89.6); <0.001	7.44 (3.9-14.0); <0.001	3.80 (2.7-5.4); <0.001
LDH (U/L)	≤238 (1671)	238.1-372.3 (1677)		372.4-478.9 (622)	≥479 (794)		238.1-372.3	372.4-478.9	≥479
Asymptomatic (n: 386)	257	117		10	2		Reference	Reference	Reference
Mild (n: 2458)	1143	1008		198	109	<0.001	12.25 (3.0-49.9); <0.001	4.45 (2.3-8.5); <0.001	1.94 (1.5-2.4); <0.001
Moderate (n: 817)	136	307		199	175		165.35 (40.4-676.7); <0.001	37.61 (19.3-73.4); <0.001	4.96 (3.7-6.7); <0.001
Severe (n: 1103)	135	245		215	508		483.54 (118.7-1969.0); <0.001	40.93 (21.0-79.8); <0.001	3.99 (2.9-5.4); <0.001
CRP (U/L)	≤0.26 (2043)	0.27-3.41 (2262)		3.42-6.40 (562)	≥6.41 (1209)		0.27-3.41	3.42-6.40	≥6.41
Asymptomatic (n: 614)	429	172		8	5		Reference	Reference	Reference
Mild (n: 3350)	1481	1517		151	201	<0.001	1.5 (0.9-2.5); 0.10	2.7 (0.8-9.6); 0.12	2.8 (1.2-6.9); <0.001
Moderate (n: 887)	81	329		249	228		1.6 (0.9-2.8); 0.15	2.4 (0.6-9.2); 0.21	3.5 (1.3-8.9); <0.001
Severe (n: 1225)	52	244		154	775		1.5 (0.8-2.9); 0.18	1.7 (0.4-7.1); 0.44	4.7 (1.8-12.3); <0.001
Procalcitonin (ng/mL)	≤0.019 (1043)	0.02-0.059 (685)		0.06-0.099 (282)	≥0.1 (579)		0.02-0.059	0.06-0.099	≥0.1
Asymptomatic (n: 189)	122	49		10	8		Reference	Reference	Reference
Mild (n: 1384)	691	390		141	162	<0.001	3.58 (1.7-7.5); <0.001	2.49 (1.3-4.9); <0.001	1.41 (1.0-2.0); 0.06
Moderate (n: 407)	121	118		56	112		14.12 (6.6-30.2); <0.001	5.65 (2.8-11.6); <0.001	2.43 (1.6-3.7); <0.001
Severe (n: 609)	109	128		75	297		41.55 (19.7-87.8); <0.001	8.39 (4.1-17.1); <0.001	2.92 (1.9-4.4); <0.001
IL-6 (pg/mL)	≤4 (1365)	4.1-15.2 (897)		15.3-28.5 (359)	≥28.6 (733)		4.1-15.2	15.3-28.5	≥28.6
Asymptomatic (n: 226)	175	33		8	10		Reference	Reference	Reference
Mild (n: 1641)	838	445		147	211	<0.001	4.41 (2.3-8.5); <0.001	3.84 (1.8-8.0); <0.001	2.82 (1.9-4.2); <0.001
Moderate (n: 604)	197	191		87	129		11.46 (5.8-22.5); <0.001	9.66 (4.6-20.5); <0.001	5.14 (3.4-7.8); <0.001
Severe (n: 883)	155	228		117	383		43.24 (22.3-84.0); <0.001	16.51 (7.8-34.9); <0.001	7.80 (5.1-11.9); <0.001
CRP/ALB (Ratio)	≤0.059 (1988)	0.06-0.88 (2294)		0.89-1.77 (617)	≥1.78 (1174)		0.06-0.88	0.89-1.77	≥1.78
Asymptomatic (n: 613)	425	176		7	5		Reference	Reference	Reference
Mild (n: 3348)	1440	1555		161	192	<0.001	11.33 (4.6-27.7); <0.001	6.79 (3.2-14.6); <0.001	2.61 (2.2-3.2); <0.001
Moderate (n: 888)	74	333		269	212		243.51 (97.0-611.4); <0.001	220.71 (100.2-486.3); <0.001	10.87 (8.0-14.8); <0.001
Severe (n: 888)	74	333		269	212		1327.00 (524.8-3355.9); <0.001	223.03 (99.1-501.8); <0.001	11.34 (7.9-16.2); <0.001
Coagulation Biomarkers									
aPTT (Seconds)	≤32.1 (2681)	32.2-36.5 (1966)		36.6-38.8 (417)	≥38.9 (474)		32.2-36.5	36.6-38.8	≥38.9
Asymptomatic (n: 507)	220	212		47	28		Reference	Reference	Reference
Mild (n: 3025)	1461	1118		224	222	<0.001	1.2 (0.79-1.81); 0.41	0.7 (0.51-1.01); 0.06	0.8 (0.65-0.97); 0.03
Moderate (n: 840)	449	270		56	65		1.1 (0.71-1.82); 0.59	0.6 (0.38-0.89); 0.01	0.6 (0.49-0.80); <0.001
Severe (n: 1166)	551	366		90	159		2.3 (1.47-3.49); <0.001	0.8 (0.52-1.13); 0.17	0.7 (0.55-0.87); <0.001
D-Dimer (ng/mL)	≤86 (1609)	87-236 (2192)		237-349 (583)	≥350 (1147)		87-236	237-349	≥350
Asymptomatic (n: 522)	298	174		26	24		Reference	Reference	Reference
Mild (n: 3046)	1186	1336		233	291	<0.001	3.05 (2.0-4.7); <0.001	2.25 (1.5-3.4); <0.001	1.93 (1.6-2.4); <0.001
Moderate (n: 822)	69	393		140	220		39.59 (24.1-65.0); <0.001	23.26 (14.2-38.1); <0.001	9.76 (7.1-13.4); <0.001
Severe (n: 1141)	56	289		184	612		135.70 (82.5-223.2); <0.001	37.66(22.8-62.1); <0.001	8.84(6.3-12.4); <0.001
Fibrinogen (mg/dL)	≤322 (2200)	323-391 (1280)		392-424 (478)	≥425 (1541)		323-391	392-424	≥425
Asymptomatic (n: 521)	358	111		21	31		Reference	Reference	Reference
Mild (n: 2999)	1511	802		238	448	<0.001	3.42 (2.3-5.0); <0.001	2.69 (1.7-4.3); <0.001	1.71 (1.4-2.2); <0.001
Moderate (n: 818)	143	172		116	387		31.25 (20.7-47.3); <0.001	13.83 (8.4-22.9); <0.001	3.88 (2.9-5.3); <0.001
Severe (n: 1161)	188	195		103	675		41.46 (27.8-61.9); <0.001	9.34 (5.7-15.4); <0.001	3.35 (2.5-4.5); <0.001
PT (Seconds)	≤11.8 (2295)	11.9-13.2 (2177)		13.2-13.9 (527)	≥14 (621)		11.9-13.2	13.2-13.9	≥14
Asymptomatic (n: 517)	200	234		45	38		Reference	Reference	Reference
Mild (n: 3093)	1317	1283		264	229	<0.001	0.92 (0.6-1.3); 0.64	0.89 (0.6-1.3); 0.52	0.83 (0.7-1.0); 0.08
Moderate (n: 840)	348	290		96	106		1.60 (1.1-2.4); <0.001	1.23 (0.8-1.8); 0.31	0.71 (0.6-0.9); <0.001
Severe (n: 1170)	430	370		122	248		3.04 (2.1-4.4); <0.001	1.26 (0.9-1.8); 0.23	0.74 (0.6-0.9); <0.001
INR (Ratio)	≤1.01(2077)	1.02-1.13 (2255)		1.13-1.15 (332)	≥1.16 (949)		1.02-1.13	1.13-1.15	≥1.16
Asymptomatic (n: 515)	167	252		38	58		Reference	Reference	Reference
Mild (n: 3092)	1176	1345		166	405	<0.001	0.99 (0.7-1.4); 0.96	0.62 (0.4-0.9); <0.001	0.76 (0.6-0.9); <0.001
Moderate (n: 840)	326	299		49	166		1.47 (1.0-2.1); <0.001	0.66 (0.4-1.0); 0.08	0.61 (0.5-0.8); <0.001
Severe (n: 1166)	408	359		79	320		2.26 (1.6-3.2); <0.001	0.85 (0.6-1.3); 0.46	0.58 (0.5-0.7); <0.001
Glycemic Biomarkers									
Glu-R (mg/dL)	≤86 (2336)	87-107.3 (1624)		107.4-113.9 (304)	≥114 (2378)		87-107.3	107.4-113.9	≥114
Asymptomatic (n: 837)	470	195		34	138		Reference	Reference	Reference
Mild (n: 3922)	1643	1071		163	1045	<0.001	2.17 (1.8-2.7); <0.001	1.37 (0.9-2.0); 0.11	1.57 (1.3-1.9); <0.001
Moderate (n: 814)	118	181		53	462		13.34 (10.1-17.6); <0.001	6.21 (3.9-10.0); <0.001	3.70 (2.8-4.9); <0.001
Severe (n: 1069)	105	177		54	733		23.78 (18.0-31.4); <0.001	7.11 (4.4-11.5); <0.001	4.06 (3.0-5.4); <0.001
HbA1c (%)	≤5.86 (2122)	5.87-5.97 (183)		5.98-6.00 (174)	≥6.01 (1397)		5.87-5.97	5.98-6.00	≥6.01
Asymptomatic (n: 284)	206	12		9	57		Reference	Reference	Reference
Mild (n: 2012)	1258	100		80	574	<0.001	1.65 (1.2-2.2); <0.001	1.46 (0.7-2.9); 0.30	1.37 (0.7-2.5); 0.32
Moderate (n: 687)	319	40		39	289		3.27 (2.3-4.6); <0.001	2.80 (1.3-5.9); <0.001	2.15 (1.1-4.2); <0.001
Severe (n: 893)	339	31		46	477		5.09 (3.7-7.0); <0.001	3.11 (1.5-6.5); <0.001	1.57 (0.8-3.1); 0.20

On multinomial logistic regression, after adjusting for other covariates, laboratory biomarkers that had at least one statistically significant category (p<0.05) across the comparison groups were found to be independent predictors of COVID-19 severity. These parameters were neutrophil percentage (>81%), lymphocyte percentage (25.4-20.6, 20.5-17.5, and ≤17.4), ALC (1.38-1.13, 1.12-0.91, and ≤0.90), AEC (0.03-0.02), TBIL (0.51-0.53 and ≥0.54), A/G ratio (≤1.49), albumin (4.2-4, 3.9-3.6, and ≤3.5), ferritin (≥445.4), LDH (238.1-372.3 and ≥479), IL-6 (4.1-15.2 and ≥28.6), CRP/ALB (0.89-1.77 and ≥1.78), D-dimer (87-236 and 237-349), and fibrinogen (≥425). Nagelkerke’s R-square test was 0.58, which can be inferred as a very good model (Table 7).

**Table 7:** Predictor of COVID-19 severity Reference: neutrophils, ≤61%; lymphocytes, ≥25.5%; eosinophils, ≥1.5%; basophils, ≥0.8%; ALC, ≥1.39×103/µL; AEC, ≥0.07×103/µL; TBIL, ≤0.41 mg/dL; A/G ratio, ≥1.72 ratio; albumin, ≥4.3 g/dL; calcium, ≥9 mg/dL; phosphorus, ≤2.8 mg/dL; sodium, ≤135 mmol/L; ferritin, ≤190.8 ng/mL; LDH, ≤238 U/L; IL-6, ≤4 pg/mL; CRP/ALB, ≤0.059 ratio; D-dimer, ≤86 ng/mL; fibrinogen, ≤322 mg/dL N, number of patients; ALC, absolute lymphocyte counts; AEC, absolute eosinophil counts; TBIL, total bilirubin; A/G, albumin/globulin; CRP/ALB, C-reactive protein/albumin; COVID-19, coronavirus disease 2019; LDH, lactate dehydrogenase; CI, confidence interval; AOR, adjusted odds ratio

Variables	AOR (95% CI); P Value		
Neutrophils (%)	61.1-64.6	64.7-80	≥81
Asymptomatic (n: 910)	Reference	Reference	Reference
Mild (n: 4153)	1.3 (0.7-2.4); 0.42	1.4 (0.7-3.0); 0.35	1.3×10^6^ (4.9×10^6^-34.3×10^6^); <0.001
Moderate (n: 862)	1.3 (0.6-2.9); 0.53	2.3 (0.9-5.7); 0.09	34.4×10^6^ (11.9×10^6^-99.4×10^6^); <0.001
Severe (n: 1223)	1.2 (0.5-3.2); 0.70	2.0 (0.7-6.0); 0.20	38.9×10^6^ (3.1×10^6^-90.1 ×10^6^);<0.001
Lymphocytes (%)	25.4-20.6	20.5-17.5	≤17.4
Asymptomatic (n: 911)	Reference	Reference	Reference
Mild (n: 4172)	0.5 (0.2-0.8); 0.01	0.4 (0.1-0.9); 0.02	0.2 (0.1-0.6); <0.001
Moderate (n: 886)	0.5 (0.2-1.2); 0.12	0.4 (0.2-1.2); 0.12	0.3 (0.1-0.9); 0.05
Severe (n: 1244)	0.5 (0.2-1.2); 0.12	0.6 (0.2-1.9); 0.36	0.7 (0.2-2.4); 0.53
Eosinophils (%)	1.4-0.6	0.6-0.4	≤0.3
Asymptomatic (n: 911)	Reference	Reference	Reference
Mild (n: 4172)	1.2 (0.6-2.3); 0.57	2.0 (0.6-6.1); 0.24	1.5 (0.6-3.8); 0.35
Moderate (n: 886)	0.9 (0.4-2.0); 0.81	1.3 (0.4-4.9); 0.66	1.4 (0.5-4.1); 0.58
Severe (n: 1244)	0.9 (0.4-2.2); 0.84	1.0 (0.3-3.9); 0.99	1.7 (0.5-5.3); 0.38
Basophils (%)	0.7-0.45	0.44-0.3	≤0.29
Asymptomatic (n: 911)	Reference	Reference	Reference
Mild (n: 4172)	1.1 (0.7-1.7); 0.60	1.4 (0.8-2.3); 0.22	1.4 (0.6-3.2); 0.41
Moderate (n: 886)	1.1 (0.6-1.8); 0.84	1.4 (0.8-2.5); 0.29	1.3 (0.5-3.1); 0.63
Severe (n: 1244)	1.2 (0.7-2.2); 0.47	1.2 (0.6-2.3); 0.56	1.4 (0.6-3.5); 0.49
ALC (10^3^/µL)	1.38-1.13	1.12-0.91	≤0.9
Asymptomatic (n: 904)	Reference	Reference	Reference
Mild (n: 4101)	2.2 (1.3-3.7); <0.001	2.2 (1.2-4.2); 0.01	2.5 (1.1-5.7); 0.03
Moderate (n: 866)	2.3 (1.2-4.4); 0.01	2.6 (1.3-5.5); 0.01	2.7 (1.1-6.6); 0.03
Severe (n: 1224)	1.8 (0.9-3.5); 0.11	2.2 (1.0-4.8); 0.05	3.4 (1.4-8.5); 0.01
AEC (10^3^/µL)	0.06-0.04	0.03-0.02	≤0.01
Asymptomatic (n: 911)	Reference	Reference	Reference
Mild (n: 4172)	1.3 (0.6-2.7); 0.54	2.1 (0.7-6.3); 0.19	0.9 (0.4-1.9); 0.77
Moderate (n: 886)	1.3 (0.5-3.3); 0.64	3.6 (1.0-13.1); 0.05	1.4 (0.6-3.6); 0.43
Severe (n: 1244)	0.9 (0.3-2.6); 0.86	3.5 (0.9-13.2); 0.07	1.4 (0.5-3.7); 0.47
TBIL (mg/dL)	0.42-0.50	0.51-0.53	≥0.54
Asymptomatic (n: 951)	Reference	Reference	Reference
Mild (n: 4277)	0.9 (0.5-1.6); 0.70	0.5 (0.3-1.2); 0.12	0.6 (0.4-0.9); 0.02
Moderate (n:)	1.0 (0.5-1.9); 0.95	0.4 (0.2-1.1); 0.08	0.6 (0.4-1.0); 0.08
Severe (n: 1259)	0.7 (0.3-1.3); 0.22	0.2 (0.1-0.5); <0.001	0.5 (0.3-0.8); 0.01
TP (g/dL)	6.84-6.61	6.60-6.45	≤6.44
Asymptomatic (n: 951)	Reference	Reference	Reference
Mild (n: 4280)	1.0 (0.6-1.6); 0.98	1.1 (0.6-2.1); 0.76	1.0 (0.5-1.8); 0.94
Moderate (n: 900)	0.8 (0.4-1.5); 0.47	0.5 (0.2-1.1); 0.08	0.7 (0.3-1.4); 0.3
Severe (n: 1260)	0.9 (0.4-1.7); 0.69	1.0 (0.4-2.3); 0.99	1.1 (0.5-2.4); 0.83
A/G Ratio	1.71-1.60	1.61-1.50	≤1.49
Asymptomatic (n: 951)	Reference	Reference	Reference
Mild (n: 4280)	1.5 (0.9-2.6); 0.14	0.9 (0.5-1.6); 0.64	0.6 (0.3-1.0); 0.07
Moderate (n: 900)	1.3 (0.7-2.6); 0.42	0.8 (0.4-1.5); 0.45	0.4 (0.2-0.8); 0.02
Severe (n: 1260)	1.7 (0.8-3.5); 0.14	1.2 (0.6-2.6); 0.58	0.7 (0.3-1.5); 0.32
Albumin (g/dL)	4.2-4	3.9-3.6	≤3.5
Asymptomatic (n: 951)	Reference	Reference	Reference
Mild (n: 4280)	1.0 (0.6-1.6); 0.93	1.2 (0.5-2.7); 0.69	1.4 (0.4-5.1); 0.57
Moderate (n: 900)	2.4 (1.3-4.5); 0.01	4.1 (1.5-10.8); 0.01	4.5 (1.1-18.4); 0.04
Severe (n: 1260)	1.1 (0.6-2.2); 0.74	1.5 (0.5-4.1); 0.47	1.8 (0.4-7.8); 0.41
Calcium (mg/dL)	8.9-8.7	8.6-8.4	≤8.3
Asymptomatic (n: 947)	Reference	Reference	Reference
Mild (n: 4269)	0.9 (0.6-1.4); 0.60	0.8 (0.5-1.4); 0.46	0.6 (0.3-1.2); 0.16
Moderate (n: 900)	0.9 (0.5-1.6); 0.67	0.8 (0.5-1.6); 0.57	0.8 (0.4-1.7); 0.51
Severe (n: 1255)	0.9 (0.5-1.7); 0.86	0.8 (0.4-1.5); 0.41	1.0 (0.4-2.2); 0.95
Phosphorus (mg/dL)	2.81-3.20	3.21-3.60	≥3.61
Asymptomatic (n: 946)	Reference	Reference	Reference
Mild (n: 4271)	0.9 (0.6-1.4); 0.62	0.9 (0.5-1.5); 0.64	1.0 (0.6-1.7); 0.92
Moderate (n: 900)	1.0 (0.6-1.7); 0.96	1.3 (0.7-2.4); 0.37	1.1 (0.6-2.0); 0.85
Severe (n: 1256)	1.3 (0.7-2.2); 0.40	1.5 (0.8-2.9); 0.17	1.4 (0.7-2.7); 0.3
Sodium (mmol/L)	136-137	138-141	≥142
Asymptomatic (n: 947)	Reference	Reference	Reference
Mild (n: 4271)	0.9 (0.4-2.3); 0.91	0.9 (0.4-2.0); 0.86	0.9 (0.4-2.1); 0.83
Moderate (n: 900)	1.0 (0.4-2.6); 0.99	0.9 (0.4-2.0); 0.73	1.0 (0.4-2.5); 0.98
Severe (n: 1257)	1.1 (0.4-2.9); 0.81	1.0 (0.4-2.2); 0.92	1.5 (0.6-3.9); 0.38
Ferritin (ng/mL)	190.9-355.5	355.6-445.3	≥445.4
Asymptomatic (n: 460)	Reference	Reference	Reference
Mild (n: 2592)	1.4 (0.9-2.3); 0.16	2.3 (0.7-8.1); 0.18	2.5 (1.0-6.1); 0.04
Moderate (n: 629)	1.5 (0.8-2.7); 0.19	2.0 (0.5-7.7); 0.29	3.1 (1.2-7.9); 0.02
Severe (n: 891)	1.5 (0.8-2.8); 0.24	1.5 (0.4-5.8); 0.6	4.2 (1.6-10.8); <0.001
LDH (U/L)	238.1-372.3	372.4-478.9	≥479
Asymptomatic (n: 386)	Reference	Reference	Reference
Mild (n: 2458)	1.6 (1.1-2.3); 0.02	1.4 (0.5-3.9); 0.46	3.5 (0.4-28.5); 0.23
Moderate (n: 817)	1.7 (1.0-2.7); 0.04	2.1 (0.7-5.9); 0.17	5.8 (0.7-48.1); 0.1
Severe (n: 1103)	1.4 (0.9-2.3); 0.16	1.6 (0.5-4.6); 0.4	8.0 (1.0-66.6); 0.05
IL-6 (pg/mL)	4.1-15.2	15.3-28.5	≥28.6
Asymptomatic (n: 226)	Reference	Reference	Reference
Mild (n: 1641)	1.7 (1.0-2.9); 0.04	1.7 (0.8-3.8); 0.18	2.4 (1.1-5.4); 0.03
Moderate (n: 604)	1.2 (0.7-2.2); 0.47	1.3 (0.5-3.2); 0.54	1.6 (0.7-3.8); 0.29
Severe (n: 883)	1.2 (0.6-2.2); 0.55	1.4 (0.6-3.4); 0.49	2.5 (1.0-6.0); 0.04
CRP/ALB (Ratio)	0.06-0.88	0.89-1.77	≥1.78
Asymptomatic (n: 613)	Reference	Reference	Reference
Mild (n: 3348)	1.1 (0.7-1.7); 0.69	1.8 (0.5-6.8); 0.39	2.2 (0.4-11.4); 0.36
Moderate (n: 888)	1.4 (0.8-2.5); 0.30	4.3 (1.1-17.8); 0.04	3.4 (0.6-19.2); 0.17
Severe (n: 888)	1.1 (0.6-2.2); 0.74	3.5 (0.8-15.1); 0.09	7.0 (1.2-40.8); 0.03
D-Dimer (ng/mL)	87-236	237-349	≥350
Asymptomatic (n: 522)	Reference	Reference	Reference
Mild (n: 3046)	1.5 (1.0-2.2); 0.06	1.8 (0.7-5.0); 0.25	0.9 (0.4-2.1); 0.88
Moderate (n: 822)	2.9 (1.6-5.0); <0.001	3.2 (1.1-10.0); 0.04	2.1 (0.9-5.4); 0.1
Severe (n: 1141)	1.4 (0.7-2.5); 0.33	1.7 (0.5-5.4); 0.37	2.0 (0.8-5.1); 0.15
Fibrinogen (mg/dL)	323-391	392-424	≥425
Asymptomatic (n: 521)	Reference	Reference	Reference
Mild (n: 2999)	1.1 (0.7-1.7); 0.70	1.1 (0.5-2.4); 0.81	2.1 (0.8-5.4); 0.11
Moderate (n: 818)	1.3 (0.7-2.2); 0.40	1.3 (0.5-3.2); 0.54	3.8 (1.4-10.1); 0.01
Severe (n: 1161)	0.9 (0.5-1.7); 0.85	1.0 (0.4-2.4); 0.93	2.0 (0.7-5.5); 0.17

Comparison between both waves (inter- and intra-wave comparison)

The number of patients admitted in the first wave were 5628 and in the second wave were 1788. Patients admitted in the first wave were older and had a shorter hospital stay than patients admitted in the first wave. In the first wave, the majority of patients were suffering from mild disease (66.4%), but in the second wave, most of the patients were having severe disease (31.2%) with a corresponding higher mortality in the second wave (2.1% versus 19.1%). The TLC, neutrophil count, NLR, PLR, and NMR were all significantly higher than the RBC parameters (Hb, HCT, RBC, MCH, MCHC, and RDW), lymphopenia, eosinopenia, basopenia, monocytopenia, and LMR in the second-wave patients.

The second-wave patients had much lower bilirubin levels, total protein, albumin, and A/G ratio with the exception of globulin. Also, it was observed that the second-wave patients had significantly higher levels of urea and creatinine but lower levels of uric acid, as well as higher levels of phosphorus and potassium but lower levels of calcium, sodium, and chloride (Table 8).

**Table 8:** Comparison of laboratory biomarkers in COVID-19 waves (first wave versus second wave) N, number of patients; IQR, interquartile range; Hb, hemoglobin; HCT, hematocrit; RBC, red blood cell; TLC, total leucocyte count; PLT, platelet; MCV, mean corpuscular volume; MCH, mean corpuscular hemoglobin; MCHC, mean corpuscular hemoglobin concentration; RDW: red cell distribution width; ANC, absolute neutrophil counts; ALC, absolute lymphocyte counts; AMC, absolute monocyte counts; AEC, absolute eosinophil counts; ABC, absolute basophil counts; NLR, neutrophil-lymphocyte ratio; PLR, platelet-to-lymphocyte ratio; LMR, lymphocyte-to-monocyte ratio; NMR, neutrophil-to-monocyte ratio; SII, systemic immune-inflammation index; PT, prothrombin time; INR, international normalized ratio; aPTT, activated partial thromboplastin time; TBIL, total bilirubin; DBIL, direct bilirubin; IBIL, indirect bilirubin; ALT/SGPT, alanine aminotransferase/serum glutamic pyruvic transaminase; AST/SGOT, aspartate aminotransferase/serum glutamic oxaloacetic transaminase; ALP, alkaline phosphatase; TP, total protein; ALB, albumin; A/G, albumin/globulin; CRP/ALB, C-reactive protein/albumin; Glu-R, serum random glucose; HbA1c, glycated hemoglobin; COVID-19, coronavirus disease 2019; LDH, lactate dehydrogenase

	First Wave (N: 5628)		Second Wave (N: 1788)		P Value
	N (%)	Median (IQR)	N (%)	Median (IQR)	
Gender					
Male	4306 (76.5)	-	1179 (65.9)	-	<0.001
Female	1318 (23.4)	-	609 (34.1)	-	
Others	4 (0.1)	-	0 (0.0)	-	
Severity					
Asymptomatic	924 (16.4)	-	30 (1.7)	-	<0.001
Mild	3738 (66.4)	-	557 (31.2)	-	
Moderate	475 (8.4)	-	430 (24.0)	-	
Severe	491 (8.7)	-	771 (43.1)	-	
Outcome					
Recovered	5509 (97.9)	-	1446 (80.9)		<0.001
Expired	119 (2.1)	-	342 (19.1)	-	
Age (Years)	5628 (75.9)	38 (29.0-51.0)	1788 (24.1)	49 (35.8-60)	<0.001
Duration (Days)	5628 (75.9)	10 (8-12)	1788 (24.1)	8 (5-12)	<0.001
Hb (g/dL)	5590 (75.9)	13.40 (12.0-14.6)	1771 (24.1)	13.0 (11.7-14.4)	<0.001
HCT (%)	5590 (75.9)	42.39 (38.5-45.8)	1771 (24.1)	41.5 (37.8-45.2)	<0.001
RBC (10^6^/µL)	5590 (75.9)	4.69 (4.3-5.1)	1771 (24.1)	4.6 (4.2-5)	<0.001
MCV (fL)	5590 (75.9)	90.50 (86.1-94.8)	1771 (24.1)	90.5 (86.4-94.9)	0.59
MCH (pg)	5590 (75.9)	28.82 (27.2-30.3)	1771 (24.1)	28.74 (27-30.2)	0.12
MCHC (g/dL)	5590 (75.9)	31.68 (30.9-32.4)	1771 (24.1)	31.5 (30.6-32.3)	<0.001
RDW (%)	5590 (75.9)	14.20 (13.4-15.2)	1771 (24.1)	14.0 (13.3-15.1)	0.01
PLT (10^3^/µL)	5590 (75.9)	206.00 (155.0-268.0)	1771 (24.1)	225.0 (165-312)	<0.001
TLC (10^3^/µL)	5590 (75.9)	5.70 (4.5-7.3)	1771 (24.1)	7.7 (5.2-11.3)	<0.001
Neutrophils (%)	5449 (75.5)	57.10 (48.8-66.5)	1764 (24.5)	78.8 (65-86.6)	<0.001
ANC (10^3^/µL)	5449 (75.5)	3.18 (2.3-4.5)	1764 (24.5)	6.0 (3.4-9.6)	<0.001
Lymphocytes (%)	5449 (75.5)	29.60 (21.4-36.6)	1764 (24.5)	12.1 (6.6-23.8)	<0.001
ALC (10^3^/µL)	5449 (75.5)	1.58 (1.1-2.1)	1764 (24.5)	0.9 (0.6-1.3)	<0.001
Eosinophils (%)	5449 (75.5)	1.40 (0.5-2.8)	1764 (24.5)	0.2 (0-0.7)	<0.001
AEC (10^3^/µL)	5449 (75.5)	0.08 (0.0-0.2)	1764 (24.5)	0.0 (0-0)	<0.001
Monocytes (%)	5449 (75.5)	6.50 (5.2-7.9)	1764 (24.5)	5.3 (3.9-7.1)	<0.001
AMC (10^3^/µL)	5449 (75.5)	0.4 (0.3-0.6)	1764 (24.5)	0.37 (0.3-0.5)	<0.001
Basophils (%)	5449 (75.5)	0.70 (0.4-1.4)	1764 (24.5)	0.4 (0.2-0.6)	<0.001
ABC (10^3^/µL)	5449 (75.5)	0.04 (0.0-0.1)	1764 (24.5)	0.0 (0-0)	<0.001
NLR (Ratio)	5449 (75.5)	1.93 (1.3-3.1)	1764 (24.5)	6.5 (2.7-13.3)	<0.001
PLR (Ratio)	5449 (75.5)	129.26 (93.3-186.0)	1764 (24.5)	242.0 (143.1-408.5)	<0.001
LMR (Ratio)	5449 (75.5)	4.30 (3.0-5.8)	1764 (24.5)	2.4 (1.3-3.8)	<0.001
SII (Ratio)	5449 (75.5)	403.21 (244.6-703.9)	1764 (24.5)	1433.8 (506.5-3589.5)	<0.001
NMR (Ratio)	5449 (75.5)	8.62 (6.4-11.7)	1764 (24.5)	14.5 (9.4-21.2)	<0.001
TBIL (mg/dL)	5610 (75.9)	0.57 (0.4-0.8)	1777 (24.1)	0.5 (0.4-0.7)	<0.001
DBIL (mg/dL)	5614 (76.0)	0.21 (0.2-0.3)	1777 (24.0)	0.2 (0.2-0.3)	<0.001
IBIL (mg/dL)	5610 (75.9)	0.36 (0.3-0.5)	1777 (24.1)	0.3 (0.2-0.4)	<0.001
SGPT/ALT (U/L)	5613 (76.0)	37.00 (23.6-60)	1777 (24.0)	48.0 (28-84)	<0.001
SGOT/AST (U/L)	5614 (76.0)	34.20 (26.6-49)	1777 (24.0)	45.0 (31-74)	<0.001
ALP (IU)	5613 (76.0)	81.0 (64-106)	1777 (24.0)	83.00 (69-105)	<0.001
TP (g/dL)	5614 (76.0)	6.81 (6.5-7.2)	1777 (24.0)	6.4 (6-6.8)	<0.001
Globulin (g/dL)	5614 (76.0)	2.56 (2.3-2.8)	1777 (24.0)	2.6 (2.3-2.8)	0.54
A/G Ratio	5614 (76.0)	1.65 (1.5-1.8)	1777 (24.0)	1.5 (1.3-1.7)	<0.001
Albumin (g/dL)	5614 (76.0)	4.30 (4-4.5)	1777 (24.0)	3.8 (3.5-4.1)	<0.001
Urea (mg/dL)	5594 (75.9)	21.40 (15-27.8)	1774 (24.1)	34.2 (23.5-53.5)	<0.001
Creatinine (mg/dL)	5600 (75.9)	0.76 (0.7-0.9)	1775 (24.1)	0.8 (0.6-0.9)	<0.001
Uric Acid (mg/dL)	5598 (75.9)	5.00 (4-6)	1775 (24.1)	4.6 (3.6-5.8)	<0.001
Calcium (mg/dL)	5600 (75.9)	8.95 (8.6-9.3)	1775 (24.1)	8.4 (8.1-8.8)	<0.001
Phosphorus (mg/dL)	5599 (75.9)	2.94 (2.5-3.5)	1774 (24.1)	3.0 (2.5-3.5)	<0.001
Sodium (mmol/L)	5600 (75.9)	140.00 (138-141)	1775 (24.1)	138.0 (136-140)	<0.001
Potassium (mmol/L)	5599 (75.9)	4.30 (4-4.6)	1775 (24.1)	4.5 (4.1-5)	<0.001
Chloride (mmol/L)	5600 (75.9)	104.00 (102-106)	1775 (24.1)	102.0 (99-105)	<0.001
Ferritin (ng/mL)	3669 (80.2)	165.40 (69.5-374.8)	903 (19.8)	600.8 (211.2-1158)	<0.001
LDH (U/L)	3069 (64.4)	253.00 (213-337)	1695 (35.6)	383.0 (265-549.5)	<0.001
CRP (U/L)	4328 (71.2)	0.44 (0.1-2.4)	1753 (28.8)	3.7 (1.2-9.2)	<0.001
Procalcitonin (ng/mL)	1968 (76.0)	0.02 (0-0.1)	621 (24.0)	0.1 (0-0.2)	<0.001
IL-6 (pg/mL)	2244 (66.9)	4.00 (1-15.9)	1110 (33.1)	13.1 (4.8-40.4)	<0.001
CRP-ALB (Ratio)	4326 (71.2)	0.10 (0-0.6)	1747 (28.8)	1.0 (0.3-2.6)	<0.001
APTT (Seconds)	3864 (69.8)	32.20 (30-34.7)	1674 (30.2)	32.7 (29.8-35.9)	<0.001
D-Dimer (ng/mL)	3916 (70.8)	114.00 (65-217)	1615 (29.2)	250.0 (147-550.5)	<0.001
Fibrinogen (mg/dL)	3874 (70.4)	326.00 (275-403)	1625 (29.6)	415.0 (341-485)	<0.001
PT (Seconds)	3954 (70.4)	11.7 (11-12.5)	1666 (29.6)	12.30 (11.6-13)	<0.001
INR (Ratio)	3947 (70.3)	1.06 (1-1.1)	1666 (29.7)	1.0 (0.9-1.1)	<0.001
Glu-R (mg/dL)	5118 (77.1)	92.00 (79-122)	1524 (22.9)	125.0 (95-188.3)	<0.001
HbA1c (%)	2470 (63.7)	5.79 (5.4-6.4)	1406 (36.3)	5.8 (5.3-6.8)	0.56

Inter (between)-COVID-19 and intra (within)-COVID-19 wave comparison as per COVID-19 mortality

Most of the patients admitted recovered from the hospital in both the COVID-19 waves, but the mortality among hospitalized patients in our study increased by 17% in the second wave.

In both the COVID-19 waves, intra-wave comparisons revealed that non-survivors had older age; low RBC parameters (Hb, HCT, and RBC); high MCV, MCHC, and RDW; leucocytosis; neutrophilia; lymphopenia; eosinopenia; monocytopenia; basopenia; low LMR; raised NLR, PLR, and NMR; deranged LFT (raised bilirubin levels and raised liver enzymes); raised serum plasma proteins (total protein, albumin, and A/G ratio); deranged renal function test (RFT) (high urea, creatinine, and uric acid); and low calcium and sodium but high phosphorus and potassium levels; all inflammatory biomarkers (ferritin, LDH, CRP, procalcitonin, IL-6, and CRP/ALB ratio) were raised, and even coagulation parameters (PT, INR, fibrinogen, and D-dimer) along with random glucose and HbA1c were significantly raised (Table 9).

**Table 9:** Inter- and intra-COVID-19 wave comparison as per the outcome of COVID-19 N, number of patients; IQR, interquartile range; Hb, hemoglobin; HCT, hematocrit; RBC, red blood cell; TLC, total leucocyte count; PLT, platelet; MCV, mean corpuscular volume; MCH, mean corpuscular hemoglobin; MCHC, mean corpuscular hemoglobin concentration; RDW: red cell distribution width; ANC, absolute neutrophil counts; ALC, absolute lymphocyte counts; AMC, absolute monocyte counts; AEC, absolute eosinophil counts; ABC, absolute basophil counts; NLR, neutrophil-lymphocyte ratio; PLR, platelet-to-lymphocyte ratio; LMR, lymphocyte-to-monocyte ratio; NMR, neutrophil-to-monocyte ratio; SII, systemic immune-inflammation index; PT, prothrombin time; INR, international normalized ratio; aPTT, activated partial thromboplastin time; TBIL, total bilirubin; DBIL, direct bilirubin; IBIL, indirect bilirubin; ALT/SGPT, alanine aminotransferase/serum glutamic pyruvic transaminase; AST/SGOT, aspartate aminotransferase/serum glutamic oxaloacetic transaminase; ALP, alkaline phosphatase; TP, total protein; ALB, albumin; A/G, albumin/globulin; CRP/ALB, C-reactive protein/albumin; Glu-R, serum random glucose; HbA1c, glycated hemoglobin; COVID-19, coronavirus disease 2019; LDH, lactate dehydrogenase

	First Wave (N: 5628)				First Wave	First Wave (N: 1788)				First Wave	Recovered	Expired
	Recovered (N: 5509)		Expired (N: 119)		Recovered Versus Expired	Recovered (N: 1446)		Expired (N: 342)		Recovered Versus Expired	First Wave Versus Second Wave	First Wave Versus Second Wave
	N	Median (IQR)	N	Median (IQR)	Total; P Value	N	Median (IQR)	N	Median (IQR)	Total; P Value	Total; P Value	Total; P Value
Age (Tears)	5509	38 (29-50)	119	62 (16-52)	5628; <0.001	1446	46 (33-57)	342	59 (50-69)	1788; <0.001	6955; <0.001	6955; 0.13
Duration (Days)	5509	10 (8-12)	119	9 (1-4)	5628; 0.03	1446	8 (5-12)	342	9 (5-14)	1788; 0.21	6955; <0.001	6955; 0.82
Hb (g/dL)	5472	13.5 (12.1-14.6)	118	11.6 (5-9.5)	5590; <0.001	1432	13.1 (11.8-14.4)	339	12.9 (11.4-14.2)	1771; 0.09	6904; <0.001	6904; <0.001
HCT (%)	5472	42.4 (38.6-45.8)	118	38.1 (17-31.5)	5590; <0.001	1432	41.6 (37.9-45.2)	339	41.3 (37.4-45)	1771; 0.52	6904; <0.001	6904; <0.001
RBC (10^6^/µL)	5472	4.7 (4.3-5.1)	118	4.2 (2-3.5)	5590; <0.001	1432	4.6 (4.2-5)	339	4.57 (4.1-5)	1771; 0.07	6904; <0.001	6904; <0.001
MCV (fL)	5472	90.5 (86.2-94.7)	118	91.6 (67-85.6)	5590; 0.14	1432	90.4 (86.4-94.5)	339	91.4 (86.6-96.5)	1771; 0.01	6904; 0.67	6904; 0.99
MCH (pg)	5472	28.8 (27.2-30.3)	118	28.3 (18-26.5)	5590; 0.12	1432	28.7 (27-30.1)	339	28.7 (27-30.4)	1771; 0.72	6904; 0.1	6904; 0.27
MCHC (g/dL)	5472	31.7 (30.9-32.4)	118	30.9 (23-29.9)	5590; <0.001	1432	31.5 (30.6-32.3)	339	31.2 (30.3-32)	1771; <0.001	6904; <0.001	6904; 0.13
RDW (%)	5472	14.2 (13.4-15.1)	118	15.1 (12-14)	5590; <0.001	1432	13.9 (13.3-14.9)	339	14.6 (13.8-15.7)	1771; <0.001	6904; <0.001	6904; 0.01
PLT (10^3^/µL)	5472	206 (155-267)	118	197.5 (12-135.8)	5590; 0.23	1432	225 (164-312)	339	234 (170.5-319)	1771; 0.59	6904; <0.001	6904; <0.001
TLC (10^3^/µL)	5472	5.7 (4.5-7.2)	118	11.4 (2-7)	5590; <0.001	1432	7.1 (4.9-10.2)	339	11.3 (8.2-15.4)	1771; <0.001	6904; <0.001	6904; 0.84
Neutrophils (%)	5333	56.8 (48.6-65.8)	116	88.2 (54-84.2)	5449; <0.001	1430	75.1 (62.3-84.5)	334	88 (82.8-90.7)	1764; <0.001	6763; <0.001	6763; 0.48
ANC (10^3^/µL)	5333	3.1 (2.3-4.4)	116	9.3 (1-6.2)	5449; <0.001	1430	5.1 (3-8.3)	334	9.96 (6.8-13.9)	1764; <0.001	6763; <0.001	6763; 0.87
Lymphocytes (%)	5333	29.8 (22-36.7)	116	5.6 (1-3.2)	5449; <0.001	1430	15.2 (8.1-26.1)	334	5.7 (3.5-9.2)	1764; <0.001	6763; <0.001	6763; 0.89
ALC (10^3^/µL)	5333	1.6 (1.2-2.1)	116	0.6 (0-0.5)	5449; <0.001	1430	1 (0.7-1.4)	334	0.6 (0.4-0.9)	1764; <0.001	6763; <0.001	6763; 0.99
Eosinophils (%)	5333	1.4 (0.5-2.8)	116	0.1 (0-0)	5449; <0.001	1430	0.2 (0.1-1)	334	0.1 (0-0.1)	1764; <0.001	6763; <0.001	6763; 0.31
AEC (10^3^/µL)	5333	0.1 (0-0.2)	116	0 (0-0)	5449; <0.001	1430	0 (0-0.1)	334	0.006 (0-0)	1764; <0.001	6763; <0.001	6763; 0.32
Monocytes (%)	5333	6.5 (5.2-8)	116	3.6 (1-2.8)	5449; <0.001	1430	5.7 (4.2-7.5)	334	4.1 (3.2-5.1)	1764; <0.001	6763; <0.001	6763; 0.05
AMC 10^3^/µL	5333	0.4 (0.3-0.5)	116	0.4 (0-0.2)	5449; 0.17	1430	0.4 (0.3-0.6)	334	0.5 (0.3-0.7)	1764; <0.001	6763; <0.001	6763; 0.04
Basophils (%)	5333	0.7 (0.4-1.4)	116	0.3 (0-0.2)	5449; <0.001	1430	0.4 (0.2-0.6)	334	0.3 (0.2-0.5)	1764; <0.001	6763; <0.001	6763; 0.85
ABC (10^3^/µL)	5333	0 (0-0.1)	116	0 (0-0)	5449; <0.001	1430	0 (0-0)	334	0.03 (0-0.1)	1764; <0.001	6763; <0.001	6763; 0.91
NLR (Ratio)	5333	1.9 (1.3-3)	116	15.8 (2-9.2)	5449; <0.001	1430	5 (2.4-10.3)	334	15.3 (9.1-25.8)	1764; <0.001	6763; <0.001	6763; 0.83
PLR (Ratio)	5333	127.8 (92.7-182.5)	116	300.8 (28-186.3)	5449; <0.001	1430	215.7 (132.4-373.6)	334	357.5 (222.9-579.5)	1764; <0.001	6763; <0.001	6763; 0.12
LMR (Ratio)	5333	4.3 (3.1-5.8)	116	1.6 (0-0.9)	5449; <0.001	1430	2.7 (1.6-4.1)	334	1.4 (0.8-2.2)	1764; <0.001	6763; <0.001	6763; 0.23
SII (Ratio)	5333	396.7 (242.1-671)	116	2550.7 (164-1307.2)	5449; 0.34	1430	1084 (436.7-2897.3)	334	3415.3 (1731.2-7032.7)	1764; 0.76	6763; 0.28	6763; 0.39
NMR (Ratio)	5333	8.5 (6.4-11.5)	116	24.4 (6-16.3)	5449; <0.001	1430	12.9 (8.6-19)	334	21.13 (16.3-27.6)	1764; <0.001	6763; <0.001	6763; 0.05
TBIL (mg/dL)	5491	0.6 (0.4-0.8)	119	0.6 (0-0.3)	5610; 0.07	1436	0.5 (0.4-0.7)	341	0.55 (0.4-0.8)	1777; <0.001	6927; <0.001	6927; 0.19
DBIL (mg/dL)	5495	0.2 (0.2-0.3)	119	0.2 (0-0.2)	5614; <0.001	1436	0.2 (0.2-0.3)	341	0.26 (0.2-0.4)	1777; <0.001	6931; 0.47	6931; 0.2
IBIL (mg/dL)	5491	0.4 (0.3-0.5)	119	0.3 (0-0.2)	5610; <0.001	1436	0.3 (0.2-0.4)	341	0.3 (0.2-0.4)	1777; 0.1	6927; <0.001	6927; 0.15
SGPT/ALT (U/L)	5494	37 (23.5-60)	119	36 (7-25)	5613; 0.71	1436	47 (27-83)	341	51 (30-89)	1777; 0.03	6930; <0.001	6930; <0.001
SGOT/AST (U/L)	5495	34 (26.4-48.2)	119	53 (11-35)	5614; <0.001	1436	43 (30-67)	341	62 (39-101)	1777; <0.001	6931; <0.001	6931; 0.22
ALP (IU)	5494	83 (68-104)	119	101 (32-75)	5613; <0.001	1436	79 (63-101)	341	93 (70-130)	1777; <0.001	6930; <0.001	6930; 0.05
TP (g/dL)	5495	6.8 (6.5-7.2)	119	5.7 (3-5.1)	5614; <0.001	1436	6.5 (6.1-6.9)	341	6.2 (5.8-6.6)	1777; <0.001	6931; <0.001	6931; <0.001
Albumin (g/dL)	5495	4.3 (4-4.5)	119	3.2 (2-2.7)	5614; <0.001	1436	3.9 (3.6-4.2)	341	3.5 (3.3-3.8)	1777; <0.001	6931; <0.001	6931; <0.001
Globulin (g/dL)	5495	2.6 (2.3-2.8)	119	2.5 (1-2.1)	5614; 0.28	1436	2.56 (2.3-2.8)	341	2.64 (2.4-3)	1777; <0.001	6931; 0.39	6931; 0.03
A/G Ratio	5495	1.7 (1.5-1.8)	119	1.3 (0-1)	5614; <0.001	1436	1.53 (1.3-1.7)	341	1.34 (1.2-1.5)	1777; <0.001	6931; <0.001	6931; 0.16
Urea (mg/dL)	5475	21.4 (15-27.8)	119	68.5 (7-42.8)	5594; <0.001	1435	30 (21.4-44.9)	339	60 (44.9-87.9)	1774; <0.001	6910; <0.001	6910; 0.43
Creatinine (mg/dL)	5481	0.8 (0.7-0.9)	119	1 (0-0.7)	5600; <0.001	1435	0.73 (0.6-0.9)	340	0.9 (0.7-1.4)	1775; <0.001	6916; <0.001	6916; 0.04
Uric Acid (mg/dL)	5479	5 (4-6)	119	5.2 (1-3.9)	5598; 0.02	1435	4.5 (3.6-5.5)	340	5.4 (4.2-7.5)	1775; <0.001	6914; <0.001	6914; 0.85
Calcium (mg/dL)	5481	9 (8.6-9.3)	119	8.2 (6-7.6)	5600; <0.001	1435	8.5 (8.1-8.8)	340	8.2 (7.9-8.6)	1775; <0.001	6916; <0.001	6916; 0.87
Phosphorus (mg/dL)	5480	2.9 (2.5-3.5)	119	3.2 (0-2.7)	5599; <0.001	1434	3 (2.5-3.5)	340	3.045 (2.5-3.8)	1774; 0.02	6914; 0.2	6914; 0.1
Sodium (mmol/L)	5481	140 (138-141)	119	138 (109-134.5)	5600; 0.04	1435	138 (136-140)	340	139 (135-143)	1775; <0.001	6916; <0.001	6916; 0.95
Potassium (mmol/L)	5480	4.3 (4-4.6)	119	4.6 (3-4.1)	5599; <0.001	1435	4.5 (4.1-4.9)	340	4.8 (4.3-5.3)	1775; <0.001	6915; <0.001	6915; 0.18
Chloride (mmol/L)	5481	104 (102-106)	119	103 (78-99)	5600; 0.29	1435	102 (99-104)	340	103 (99-107)	1775; 0.01	6916; <0.001	6916; 0.27
Ferritin (ng/mL)	3555	158.1 (67.8-354.5)	114	842.5 (46-364.2)	3669; <0.001	699	454.9 (163.6-1004.1)	206	1049.5 (591.9-1650)	905; <0.001	4254; <0.001	4254; 0.09
LDH (U/L)	2967	251 (212-327)	102	537 (141-356.3)	3069; <0.001	1375	333 (248.5-466)	321	656.5 (503.3-874.3)	1696; <0.001	4342; <0.001	4342; 0.04
CRP (U/L)	4215	0.4 (0.1-2.2)	113	12.2 (0-4.1)	4328; <0.001	1420	2.752 (0.8-7.3)	335	9.371 (5.1-15.7)	1755; <0.001	5635; <0.001	5635; 0.35
Procalcitonin (ng/mL)	1888	0 (0-0.1)	80	0.3 (0-0.1)	1968; <0.001	444	0.04 (0-0.1)	180	0.15 (0.1-0.6)	624; <0.001	2332; <0.001	2332; 0.02
IL-6 (pg/mL)	2151	3.7 (0.9-13.7)	93	57.8 (0-22.1)	2244; <0.001	830	9.4 (3.5-25.2)	281	40.3 (14.8-97.2)	1111; <0.001	2981; <0.001	2981; 0.05
CRP/ALB (Ratio)	4213	0.1 (0-0.5)	113	3.9 (0-1.5)	4326; <0.001	1414	0.75 (0.2-2)	334	2.7 (1.4-4.7)	1748; <0.001	5627; <0.001	5627; 0.04
APTT (Seconds)	3757	32.2 (30-34.6)	107	33.5 (17-28.6)	3864; 0.15	1355	32.7 (29.9-35.7)	319	32.6 (29.5-36.5)	1674; 0.59	5112; 0.005	5112; 0.83
D-Dimer (ng/mL)	3812	111 (64-201)	104	758.5 (141-385)	3916; <0.001	1307	214 (131-376)	313	756 (366-2862.3)	1620; <0.001	5119; <0.001	5119; 0.69
Fibrinogen (mg/dL)	3766	324 (274-401)	108	409.5 (110-323.3)	3874; <0.001	1308	405 (337-479)	323	439 (364-512)	1631; <0.001	5074; <0.001	5074; 0.03
PT (Seconds)	3838	12.2 (11.6-13)	117	13.7 (10-12.5)	3955; <0.001	1348	11.6 (11-12.4)	324	12.1 (11.2-13.4)	1672; <0.001	5186; <0.001	5186; <0.001
INR (Ratio)	3831	1.1 (1-1.1)	117	1.2 (1-1.1)	3948; <0.001	1348	1 (0.9-1.1)	324	1.05 (1-1.2)	1672; <0.001	5179; <0.001	5179; <0.001
Glu-R (mg/dL)	5020	91 (79-121)	98	154.5 (31-107.3)	5118; <0.001	1255	116 (93-171)	269	170 (122-272)	1524; <0.001	6275; <0.001	6275; 0.03
HbA1c (%)	2393	5.7 (5.4-6.3)	77	6.7 (4-6)	2470; <0.001	1151	5.7 (5.2-6.5)	255	6.3 (5.7-7.6)	1406; <0.001	3544; <0.001	3544; 0.05

A similar pattern of laboratory biomarkers was detected in the inter-wave comparison among recovered patients as in the intra-wave comparison. However, when comparing non-survivor groups between the waves, only a few biomarkers such as RBC parameters (Hb, HCT, RBC, and RDW), platelet count, monocyte percentage, NMR, SGPT, ALP, serum plasma protein levels, creatinine levels, LDH, procalcitonin, IL-6, CRP/ALB ratio, PT, and INR showed significant differences (Table 9).

Inter- and intra-COVID-19 wave comparison as per severity of the disease

In both waves, significant differences in most of the laboratory biomarkers at hospital admission were associated with disease severity (intra-wave). Patients with severe COVID-19 were older and had low RBC parameters (Hb, HCT, and RBC) and high MCV, MCHC, and RDW along with leucocytosis and neutrophilia, lymphopenia, eosinopenia, basopenia, and monocytopenia. They had raised NLR, PLR, SII, and NMR except LMR. Patients with severe COVID-19 also displayed higher bilirubin levels, elevated liver enzymes, deranged RFT, deranged serum plasma protein, and deranged serum electrolyte levels. Inflammatory biomarkers such as ferritin, LDH, CRP, procalcitonin, IL-6, and CRP/ALB were also raised in these patients. COVID-19 coagulopathy was also reported in severe COVID-19 patients. Severe COVID-19 was also associated with poor glycemic control (Table 10).

**Table 10:** Intra-COVID-19 wave comparison as per severity of the disease (asymptomatic versus mild versus moderate versus severe) N, number of patients; IQR, interquartile range; Hb, hemoglobin; HCT, hematocrit; RBC, red blood cell; TLC, total leucocyte count; PLT, platelet; MCV, mean corpuscular volume; MCH, mean corpuscular hemoglobin; MCHC, mean corpuscular hemoglobin concentration; RDW: red cell distribution width; ANC, absolute neutrophil counts; ALC, absolute lymphocyte counts; AMC, absolute monocyte counts; AEC, absolute eosinophil counts; ABC, absolute basophil counts; NLR, neutrophil-lymphocyte ratio; PLR, platelet-to-lymphocyte ratio; LMR, lymphocyte-to-monocyte ratio; NMR, neutrophil-to-monocyte ratio; SII, systemic immune-inflammation index; PT, prothrombin time; INR, international normalized ratio; aPTT, activated partial thromboplastin time; TBIL, total bilirubin; DBIL, direct bilirubin; IBIL, indirect bilirubin; ALT/SGPT, alanine aminotransferase/serum glutamic pyruvic transaminase; AST/SGOT, aspartate aminotransferase/serum glutamic oxaloacetic transaminase; ALP, alkaline phosphatase; TP, total protein; ALB, albumin; A/G, albumin/globulin; CRP/ALB, C-reactive protein/albumin; Glu-R, serum random glucose; HbA1c, glycated hemoglobin; COVID-19, coronavirus disease 2019; LDH, lactate dehydrogenase

	First Wave										Second Wave									
	Asymptomatic		Mild		Moderate		Severe				Asymptomatic		Mild		Moderate		Severe		Total	
	N	Median (IQR)	N	Median (IQR)	N	Median (IQR)	N	Median (IQR)	Total	P Value	N	Median (IQR)	N	Median (IQR)	N	Median (IQR)	N	Median (IQR)	Total	P Value
Age (Years)	924	34.0 (27-47)	3738	36.0 (29-49)	475	48.0 (37-58)	491	54.0 (40-65)	5628	<0.001	30	34.5 (24.3-40.8)	557	37.0 (28-52)	430	49.5 (38-60)	771	55.0 (44-64)	1788	<0.001
Duration (Days)	924	10.0 (9-13)	3738	10.0 (8-12)	475	10.0 (7-12)	491	10.0 (8-14)	5628	<0.001	30	6.0 (3.3-7)	557	7.0 (5-10)	430	8.0 (5-12)	771	9.0 (6-15)	1788	<0.001
Mortality	924	0 (0%)	3738	1 (0.02%)	475	1 (0.2%)	491	117 (23.8%)	5628	<0.001	30	0 (0%)	557	0 (0%)	430	8 (1.9%)	771	334 (43.3%)	1788	<0.001
Hb (g/dL)	916	13.8 (12.5-14.8)	3715	13.6 (12.2-14.7)	470	12.6 (11.4-14)	489	12.2 (10.9-13.7)	5590	<0.001	29	13.7 (12.1-14.9)	550	13.1 (11.8-14.7)	425	13.1 (11.8-14.2)	767	13.0 (11.6-14.3)	1771	0.53
HCT (%)	916	43.6 (39.9-46.3)	3715	42.8 (39-46)	470	39.9 (36.5-43.6)	489	38.9 (34.5-43)	5590	<0.001	29	43.0 (39.1-46.7)	550	41.7 (38.2-45.8)	425	41.5 (38-44.6)	767	41.2 (37.4-44.9)	1771	0.28
RBC (10^6^/µL)	916	4.8 (4.4-5.2)	3715	4.7 (4.3-5.1)	470	4.5 (4.1-4.9)	489	4.3 (3.8-4.8)	5590	<0.001	29	4.8 (4.4-5.4)	550	4.7 (4.2-5.1)	425	4.6 (4.2-5)	767	4.6 (4.1-5)	1771	0.01
MCV (fL)	916	91.0 (86.3-95.2)	3715	90.5 (86.2-94.7)	470	90.2 (86-93.8)	489	90.1 (85.5-95.4)	5590	0.17	29	89.6 (84.6-92.2)	550	90.1 (86.7-94.1)	425	90.3 (86.5-94.6)	767	91.1 (86.1-95.6)	1771	0.10
MCH (pg)	916	28.9 (27.3-30.4)	3715	28.8 (27.2-30.3)	470	28.7 (26.9-30)	489	28.5 (26.8-30.1)	5590	0.30	29	28.2 (26.5-30.4)	550	28.7 (27.2-30.1)	425	28.7 (27-30)	767	28.8 (27-30.3)	1771	0.98
MCHC (g/dL)	916	31.6 (30.9-32.3)	3715	31.7 (30.9-32.4)	470	31.7 (30.8-32.4)	489	31.6 (30.6-32.3)	5590	0.25	29	31.4 (30.6-32.8)	550	31.6 (30.7-32.5)	425	31.4 (30.6-32.3)	767	31.4 (30.6-32.1)	1771	0.15
RDW (%)	916	14.2 (13.4-15)	3715	14.1 (13.4-15.1)	470	14.3 (13.5-15.4)	489	14.5 (13.7-15.7)	5590	<0.001	29	13.8 (13-14.7)	550	13.8 (13.1-14.8)	425	14.0 (13.3-14.9)	767	14.2 (13.5-15.4)	1771	<0.001
PLT (10^3^/µL)	916	218.0 (163-284)	3715	200.0 (153-258)	470	217.0 (163-288.5)	489	213.0 (160-289)	5590	<0.001	29	183.0 (122-246)	550	202.0 (155.3-265.8)	425	251.0 (177-338)	767	236.0 (170-325.5)	1771	<0.001
TLC (10^3^/µL)	916	5.9 (4.8-7.3)	3715	5.4 (4.4-6.9)	470	6.5 (4.8-8.7)	489	7.5 (5.3-11.3)	5590	<0.001	29	5.4 (4-7.1)	550	5.4 (4.2-7.5)	425	8.2 (5.5-11.4)	767	9.7 (6.8-13.2)	1771	<0.001
Neutrophils (%)	882	54.1 (47-60.8)	3623	55.5 (47.5-63.6)	462	70.1 (60.5-81)	482	78.6 (66.3-87.4)	5449	<0.001	29	57.8 (49.4-64.6)	549	62.3 (53-71.1)	424	80.6 (71.5-86.1)	762	85.3 (78.6-89.6)	1764	<0.001
ANC (10^3^/µL)	882	3.2 (2.4-4.2)	3623	3.0 (2.2-4)	462	4.4 (3-6.5)	482	5.7 (3.4-9.2)	5449	<0.001	29	3.0 (2.2-4.5)	549	3.3 (2.3-5.2)	424	6.6 (3.8-9.7)	762	8.3 (5.5-11.5)	1764	<0.001
Lymphocytes (%)	882	31.8 (25.9-37.3)	3623	31.0 (24-37.7)	462	18.8 (11.1-26.5)	482	12.5 (6.4-22.4)	5449	<0.001	29	28.8 (26.4-35.9)	549	25.6 (17.8-33.7)	424	11.0 (7.2-18.3)	762	7.5 (4.6-12.5)	1764	<0.001
ALC (10^3^/µL)	882	1.8 (1.5-2.3)	3623	1.6 (1.2-2.1)	462	1.2 (0.8-1.6)	482	0.9 (0.6-1.4)	5449	<0.001	29	1.8 (1.2-2.4)	549	1.3 (1-1.7)	424	0.9 (0.6-1.2)	762	0.7 (0.5-1)	1764	<0.001
Eosinophils (%)	882	2.2 (1.2-4)	3623	1.5 (0.6-2.8)	462	0.4 (0.1-1.5)	482	0.1 (0-0.7)	5449	<0.001	29	1.7 (1-3.5)	549	0.8 (0.2-2)	424	0.1 (0-0.4)	762	0.1 (0-0.2)	1764	<0.001
AEC (10^3^/µL)	882	0.1 (0.1-0.2)	3623	0.1 (0-0.2)	462	0.0 (0-0.1)	482	0.01 (0-0.1)	5449	<0.001	29	0.1 (0.1-0.2)	549	0.0 (0-0.1)	424	0.01 (0-0)	762	0.01 (0-0)	1764	<0.001
Monocytes (%)	882	6.4 (5.2-7.8)	3623	6.7 (5.4-8.1)	462	6.0 (4.4-7.6)	482	5.1 (3.6-7)	5449	<0.001	29	6.5 (5.2-7.5)	549	6.7 (5.3-8.5)	424	5.2 (3.9-6.8)	762	4.4 (3.3-5.8)	1764	<0.001
AMC (10^3^/µL)	882	0.4 (0.3-0.5)	3623	0.4 (0.3-0.5)	462	0.4 (0.3-0.5)	482	0.38 (0.3-0.5)	5449	0.11	29	0.3 (0.2-0.5)	549	0.4 (0.3-0.5)	424	0.4 (0.3-0.6)	762	0.4 (0.3-0.6)	1764	0.01
Basophils (%)	882	0.9 (0.5-1.5)	3623	0.7 (0.4-1.5)	462	0.5 (0.3-0.9)	482	0.4 (0.2-1)	5449	<0.001	29	0.5 (0.3-0.7)	549	0.5 (0.3-0.7)	424	0.3 (0.2-0.5)	762	0.3 (0.2-0.5)	1764	<0.001
ABC (10^3^/µL)	882	0.1 (0-0.1)	3623	0.0 (0-0.1)	462	0.0 (0-0.1)	482	0.03 (0-0.1)	5449	<0.001	29	0.03 (0-0)	549	0.03 (0-0)	424	0.03 (0-0)	762	0.03 (0-0)	1764	0.34
NLR (Ratio)	882	1.7 (1.3-2.4)	3623	1.8 (1.3-2.6)	462	3.8 (2.3-7.2)	482	6.3 (3-13.4)	5449	<0.001	29	2.0 (1.3-2.4)	549	2.5 (1.6-4)	424	7.3 (3.9-11.9)	762	11.2 (6.3-19.6)	1764	<0.001
PLR (Ratio)	882	120.2 (87-157)	3623	121.9 (89.3-171.1)	462	187.5 (124.8-304.1)	482	223.3 (138.5-382)	5449	<0.001	29	107.5 (95-143.9)	549	152.4 (102.4-217.2)	424	287.3 (173.3-435.5)	762	325.6 (202.6-513.7)	1764	<0.001
LMR (Ratio)	882	4.8 (3.7-6.3)	3623	4.5 (3.3-5.9)	462	3.1 (2-4.3)	482	2.6 (1.5-3.9)	5449	<0.001	29	5.0 (3.9-6.2)	549	3.6 (2.5-5)	424	2.2 (1.3-3.5)	762	1.7 (1-2.8)	1764	<0.001
SII (Ratio)	882	369.7 (236.1-563.9)	3623	358.0 (225.3-573.8)	462	793.8 (446.7-1797)	482	1315.0 (532.4-3083.8)	5449	<0.001	29	328.4 (211.2-498.2)	549	477.9 (282.1-950.9)	424	1810.0 (764.8-3626.9)	762	2664.8 (1259.8-5368.7)	1764	0.05
NMR (Ratio)	882	8.3 (6.4-10.7)	3623	8.2 (6.1-10.8)	462	11.6 (8.1-17.5)	482	15.0 (9.5-24.4)	5449	<0.001	29	9.6 (5.8-12.2)	549	9.2 (6.4-12.8)	424	14.9 (10.9-20.5)	762	18.9 (13.9-26.1)	1764	<0.001
TBIL (mg/dL)	921	0.6 (0.5-0.8)	3725	0.6 (0.4-0.8)	474	0.5 (0.4-0.7)	490	0.5 (0.4-0.7)	5610	<0.001	30	0.5 (0.3-0.7)	552	0.4 (0.3-0.6)	426	0.5 (0.4-0.6)	769	0.5 (0.4-0.8)	1777	<0.001
DBIL (mg/dL)	921	0.2 (0.2-0.3)	3728	0.2 (0.2-0.3)	474	0.2 (0.1-0.3)	491	0.2 (0.2-0.3)	5614	0.09	30	0.2 (0.1-0.3)	552	0.2 (0.1-0.2)	426	0.2 (0.2-0.3)	769	0.2 (0.2-0.4)	1777	<0.001
IBIL (mg/dL)	921	0.4 (0.3-0.6)	3725	0.4 (0.3-0.5)	474	0.3 (0.2-0.5)	490	0.3 (0.2-0.4)	5610	<0.001	30	0.3 (0.2-0.4)	552	0.3 (0.2-0.4)	426	0.3 (0.2-0.4)	769	0.3 (0.2-0.4)	1777	0.01
SGPT/ALT (U/L)	921	34.3 (21-55)	3728	36.5 (23.6-59)	473	40.0 (27-75)	491	40.0 (25.7-67.5)	5613	<0.001	30	32.5 (20.3-50.8)	552	35.0 (23-66)	426	52.0 (31-96)	769	53.0 (31-93)	1777	<0.001
SGOT/AST (U/L)	921	31.0 (25-42)	3728	33.8 (26-47)	474	43.0 (29.6-67)	491	45.0 (31-71)	5614	<0.001	30	34.5 (24-50.5)	552	35.0 (26.8-51)	426	49.0 (32-78)	769	56.0 (37-89)	1777	<0.001
ALP (IU)	921	85.0 (71-106)	3728	82.0 (68-102)	473	83.0 (66-110)	491	87.0 (68-121)	5613	0.01	30	81.0 (63.3-95)	552	77.0 (63.8-95)	426	77.5 (60.3-102)	769	87.0 (67-119)	1777	<0.001
TP (g/dL)	921	7.0 (6.6-7.3)	3728	6.8 (6.5-7.2)	474	6.6 (6.2-6.9)	491	6.4 (5.9-6.8)	5614	<0.001	30	6.8 (6.5-7.3)	552	6.7 (6.3-7)	426	6.4 (6-6.8)	769	6.2 (5.8-6.6)	1777	<0.001
Albumin (g/dL)	921	4.4 (4.2-4.6)	3728	4.3 (4.1-4.5)	474	4.0 (3.7-4.2)	491	3.7 (3.3-4.1)	5614	<0.001	30	4.3 (4.1-4.6)	552	4.1 (3.8-4.4)	426	3.8 (3.6-4.1)	769	3.6 (3.4-3.9)	1777	<0.001
Globulin (g/dL)	921	2.6 (2.3-2.8)	3728	2.6 (2.3-2.8)	474	2.6 (2.3-2.8)	491	2.6 (2.3-2.9)	5614	0.47	30	2.5 (2.3-2.8)	552	2.5 (2.3-2.8)	426	2.5 (2.3-2.9)	769	2.6 (2.3-2.9)	1777	0.03
A/G Ratio	921	1.7 (1.5-1.9)	3728	1.7 (1.5-1.9)	474	1.5 (1.3-1.8)	491	1.5 (1.2-1.7)	5614	<0.001	30	1.7 (1.5-1.9)	552	1.6 (1.4-1.8)	426	1.5 (1.3-1.7)	769	1.4 (1.2-1.6)	1777	<0.001
Urea (mg/dL)	915	19.3 (13-25)	3717	20.0 (15-25.7)	474	30.0 (21.4-40.7)	488	34.2 (21.4-57.8)	5594	<0.001	30	21.4 (19-28)	551	24.0 (19.3-32.1)	426	36.0 (25.7-49.2)	767	47.1 (34-66.3)	1774	<0.001
Creatinine (mg/dL)	917	0.7 (0.6-0.9)	3720	0.8 (0.7-0.9)	474	0.8 (0.7-0.9)	489	0.8 (0.7-1.1)	5600	<0.001	30	0.7 (0.6-0.8)	551	0.7 (0.6-0.8)	426	0.7 (0.6-0.9)	768	0.8 (0.7-1)	1775	<0.001
Uric Acid (mg/dL)	917	5.1 (4.1-6.1)	3718	5.0 (4.1-5.9)	474	4.6 (3.7-5.7)	489	5.0 (3.9-6.4)	5598	<0.001	30	4.7 (3.8-5.3)	551	4.6 (3.6-5.5)	426	4.4 (3.5-5.5)	768	4.8 (3.7-6.2)	1775	0.03
Calcium (mg/dL)	917	9.1 (8.8-9.5)	3720	9.0 (8.6-9.3)	474	8.8 (8.4-9.1)	489	8.5 (8.1-8.9)	5600	<0.001	30	8.8 (8.4-9.2)	551	8.6 (8.4-9)	426	8.5 (8.1-8.7)	768	8.3 (7.9-8.6)	1775	<0.001
Phosphorus (mg/dL)	916	3.1 (2.7-3.7)	3720	2.9 (2.5-3.4)	474	2.9 (2.4-3.4)	489	3.0 (2.5-3.6)	5599	<0.001	30	3.1 (2.7-4)	551	3.1 (2.6-3.5)	426	2.9 (2.4-3.5)	767	3.0 (2.5-3.6)	1774	0.02
Sodium (mmol/L)	917	140.0 (139-141)	3720	140.0 (138-141)	474	138.0 (136-140)	489	138.0 (135-141)	5600	<0.001	30	138.0 (138-139.8)	551	139.0 (137-140)	426	138.0 (135-140)	768	138.0 (135-141)	1775	<0.001
Potassium (mmol/L)	916	4.3 (4-4.6)	3720	4.3 (3.9-4.6)	474	4.4 (4-4.8)	489	4.5 (4.1-4.9)	5599	<0.001	30	4.4 (4.2-4.8)	551	4.3 (4-4.7)	426	4.6 (4.1-5)	768	4.7 (4.2-5.1)	1775	<0.001
Chloride (mmol/L)	917	105.0 (103-106)	3720	105.0 (103-106)	474	103.0 (100-105)	489	103.0 (99-106)	5600	<0.001	30	104.0 (103-105.8)	551	103.0 (101-105)	426	101.0 (99-104)	768	101.0 (98-104)	1775	<0.001
Ferritin (ng/mL)	449	94.8 (44.6-185.8)	2350	136.4 (61-283.8)	429	307.9 (154.3-686.4)	441	555.3 (222-1123.1)	3669	<0.001	11	84.9 (30.4-194.8)	242	163.6 (68.5-430.8)	200	682.4 (329.2-1108.4)	450	856.4 (455.9-1547.9)	903	<0.001
LDH (U/L)	358	223.0 (201-255)	1929	243.0 (207-297)	409	326.0 (252-438)	373	381.0 (282-525)	3069	<0.001	28	212.0 (189.8-245.5)	529	252.0 (210-310)	408	390.5 (303-487.5)	730	528.0 (399.3-717.3)	1695	<0.001
CRP (U/L)	584	0.1 (0-0.4)	2809	0.3 (0.1-1.3)	468	3.6 (1.2-7.9)	467	7.7 (2.1-14.7)	4328	<0.001	30	0.2 (0-0.6)	543	0.8 (0.2-1.7)	422	4.1 (2.3-6)	758	9.2 (4.9-14.8)	1753	<0.001
Procalcitonin (ng/mL)	188	0.0 (0-0)	1242	0.0 (0-0)	280	0.0 (0-0.1)	258	0.1 (0-0.3)	1968	<0.001	1	0.01 (0-0)	142	0.03 (0-0.1)	127	0.04 (0-0.1)	351	0.1 (0-0.2)	621	<0.001
IL-6 (pg/mL)	221	1.3 (0-3.6)	1377	3.4 (0.9-12)	342	6.7 (1.9-24.7)	304	13.8 (3.2-47.2)	2244	<0.001	5	2.2 (0.7-5.4)	264	5.1 (2.4-12.1)	262	10.6 (4.3-27)	579	24.2 (8.9-77)	1110	<0.001
CRP/ALB (Ratio)	583	0.0 (0-0.1)	2808	0.1 (0-0.3)	468	0.9 (0.3-2)	467	2.2 (0.6-4.2)	4326	<0.001	30	0.04 (0-0.2)	540	0.2 (0.1-0.4)	420	1.1 (0.6-1.6)	757	2.6 (1.3-4.1)	1747	<0.001
APTT (Seconds)	479	32.6 (30.3-35.0)	2506	32.2 (30.2-34.6)	437	31.4 (29.4-34)	442	32.3 (29.4-35.4)	3864	<0.001	28	32.7 (29.5-35.6)	519	32.9 (30.1-36)	403	32.4 (29.6-34.9)	724	32.5 (29.6-36.1)	1674	0.42
D-Dimer (ng/mL)	496	76.0 (50.0-128.0)	2547	101.0 (60-175)	436	191.5 (117.8-357)	437	282.0 (152-668)	3916	<0.001	26	115.5 (73.3-155.8)	499	142.0 (92.5-222.5)	386	234.0 (167.3-386.5)	704	459.0 (246.5-1448.5)	1615	<0.001
Fibrinogen (mg/dL)	493	294.0 (258.0-339.0)	2498	316.0 (270-378)	435	406.0 (328-485)	448	419.5 (331-509)	3874	<0.001	28	310.0 (284.8-354)	501	351.0 (307-401)	383	431.0 (377-494.5)	713	457.0 (390-528)	1625	<0.001
PT (Seconds)	489	12.1 (11.6-12.8)	2571	12.1 (11.6-12.8)	441	12.6 (11.8-13.6)	453	13.0 (12.1-14.2)	3954	<0.001	28	11.6 (11-12)	522	11.4 (10.9-12)	399	11.6 (10.9-12.4)	717	11.9 (11.2-13)	1666	<0.001
INR (ratio)	487	1.1 (1.0-1.1)	2570	1.1 (1-1.1)	441	1.1 (1-1.2)	449	1.1 (1-1.2)	3947	<0.001	28	1.03 (1-1.1)	522	1.0 (0.9-1.1)	399	1.0 (0.9-1.1)	717	1.0 (1-1.1)	1666	<0.001
Glu-R (mg/dL)	811	84.0 (76.0-101.0)	3435	90.0 (79-114)	441	116.0 (91-168)	431	133.0 (97-200)	5118	<0.001	26	84.5 (80-94.3)	487	98.0 (83-126)	373	134.0 (101-205)	638	152.0 (112-230)	1524	<0.001
HbA1c (%)	262	5.6 (5.3-6.0)	1596	5.7 (5.4-6.2)	336	6.0 (5.6-6.9)	276	6.3 (5.7-7.6)	2470	0.01	22	5.1 (4.9-5.3)	416	5.3 (5-6)	351	5.8 (5.4-6.9)	617	6.1 (5.5-7.3)	1406	<0.001

On comparing laboratory biomarkers among severity groups in the first wave with the second wave (inter-wave), most of the parameters behaved similarly as observed in intra-wave comparison. Only duration, RDW, basopenia, urea, calcium, D-dimer, PT, INR, and HbA1c had significant differences among all the severity groups. The rest of the laboratory biomarkers showed no difference in either severity groups (Table 11 and Table 12).

**Table 11:** Inter-COVID-19 wave (first wave versus second wave) comparison among disease severity groups N, number of patients; IQR, interquartile range; Hb, hemoglobin; HCT, hematocrit; RBC, red blood cell; TLC, total leucocyte count; PLT, platelet; MCV, mean corpuscular volume; MCH, mean corpuscular hemoglobin; MCHC, mean corpuscular hemoglobin concentration; RDW: red cell distribution width; ANC, absolute neutrophil counts; ALC, absolute lymphocyte counts; AMC, absolute monocyte counts; AEC, absolute eosinophil counts; ABC, absolute basophil counts; NLR, neutrophil-lymphocyte ratio; PLR, platelet-to-lymphocyte ratio; LMR, lymphocyte-to-monocyte ratio; NMR, neutrophil-to-monocyte ratio; SII, systemic immune-inflammation index; PT, prothrombin time; INR, international normalized ratio; aPTT, activated partial thromboplastin time; TBIL, total bilirubin; DBIL, direct bilirubin; IBIL, indirect bilirubin; ALT/SGPT, alanine aminotransferase/serum glutamic pyruvic transaminase; AST/SGOT, aspartate aminotransferase/serum glutamic oxaloacetic transaminase; ALP, alkaline phosphatase; TP, total protein; ALB, albumin; A/G, albumin/globulin; CRP/ALB, C-reactive protein/albumin; Glu-R, serum random glucose; HbA1c, glycated hemoglobin; COVID-19, coronavirus disease 2019; LDH, lactate dehydrogenase

	First Wave					Second Wave				
	Asymptomatic	Mild	Moderate	Severe		Asymptomatic	Mild	Moderate	Severe	
	Median (IQR); N	Median (IQR); N	Median (IQR); N	Median (IQR); N	Total; P Value	Median (IQR); N	Median (IQR); N	Median (IQR); N	Median (IQR); N	Total; P Value
Age (Years)	34.0 (27-47); 924	36.0 (29-49); 3738	48.0 (37-58); 475	54.0 (40-65); 491	5628; <0.001	34.5 (24.3-40.8); 30	37.0 (28-52); 557	49.5 (38-60); 430	55.0 (44-64); 771	1788; <0.001
Duration (Days)	10.0 (9-13); 924	10.0 (8-12); 3738	10.0 (7-12); 475	10.0 (8-14); 491	5628; <0.001	6.0 (3.3-7); 30	7.0 (5-10); 557	8.0 (5-12); 430	9.0 (6-15); 771	1788; <0.001
Mortality	0 (0%); 924	1 (0.02%); 3738	1 (0.2%); 475	117 (23.8%); 491	5628; <0.001	0 (0%); 30	0 (0%); 557	8 (1.9%); 430	334 (43.3%); 771	1788; <0.001
Hb (g/dL)	13.8 (12.5-14.8); 916	13.6 (12.2-14.7); 3715	12.6 (11.4-14); 470	12.2 (10.9-13.7); 489	5590; <0.001	13.7 (12.1-14.9); 29	13.1 (11.8-14.7); 550	13.1 (11.8-14.2); 425	13.0 (11.6-14.3); 767	1771; 0.53
HCT (%)	43.6 (39.9-46.3); 916	42.8 (39-46); 3715	39.9 (36.5-43.6); 470	38.9 (34.5-43); 489	5590; <0.001	43.0 (39.1-46.7); 29	41.7 (38.2-45.8); 550	41.5 (38-44.6); 425	41.2 (37.4-44.9); 767	1771; 0.28
RBC (10^6^/µL)	4.8 (4.4-5.2); 916	4.7 (4.3-5.1); 3715	4.5 (4.1-4.9); 470	4.3 (3.8-4.8); 489	5590; <0.001	4.8 (4.4-5.4); 29	4.7 (4.2-5.1); 550	4.6 (4.2-5); 425	4.6 (4.1-5); 767	1771; 0.01
MCV (fL)	91.0 (86.3-95.2); 916	90.5 (86.2-94.7); 3715	90.2 (86-93.8); 470	90.1 (85.5-95.4); 489	5590; 0.17	89.6 (84.6-92.2); 29	90.1 (86.7-94.1); 550	90.3 (86.5-94.6); 425	91.1 (86.1-95.6); 767	1771; 0.1
MCH (pg)	28.9 (27.3-30.4); 916	28.8 (27.2-30.3); 3715	28.7 (26.9-30); 470	28.5 (26.8-30.1); 489	5590; 0.3	28.2 (26.5-30.4); 29	28.7 (27.2-30.1); 550	28.7 (27-30); 425	28.8 (27-30.3); 767	1771; 0.98
MCHC (g/dL)	31.6 (30.9-32.3); 916	31.7 (30.9-32.4); 3715	31.7 (30.8-32.4); 470	31.6 (30.6-32.3); 489	5590; 0.25	31.4 (30.6-32.8); 29	31.6 (30.7-32.5); 550	31.4 (30.6-32.3); 425	31.4 (30.6-32.1); 767	1771; 0.15
RDW (%)	14.2 (13.4-15); 916	14.1 (13.4-15.1); 3715	14.3 (13.5-15.4); 470	14.5 (13.7-15.7); 489	5590; <0.001	13.8 (13-14.7); 29	13.8 (13.1-14.8); 550	14.0 (13.3-14.9); 425	14.2 (13.5-15.4); 767	1771; <0.001
PLT (10^3^/µL)	218.0 (163-284); 916	200.0 (153-258); 3715	217.0 (163-288.5); 470	213.0 (160-289); 489	5590; <0.001	183.0 (122-246); 29	202.0 (155.3-265.8); 550	251.0 (177-338); 425	236.0 (170-325.5); 767	1771; <0.001
TLC (10^3^/µL)	5.9 (4.8-7.3); 916	5.4 (4.4-6.9); 3715	6.5 (4.8-8.7); 470	7.5 (5.3-11.3); 489	5590; <0.001	5.4(4-7.1);29	5.4 (4.2-7.5); 550	8.2 (5.5-11.4); 425	9.7 (6.8-13.2); 767	1771; <0.001
Neutrophils (%)	54.1 (47-60.8); 882	55.5 (47.5-63.6); 3623	70.1 (60.5-81); 462	78.6 (66.3-87.4); 482	5449; <0.001	57.8 (49.4-64.6); 29	62.3 (53-71.1); 549	80.6 (71.5-86.1); 424	85.3 (78.6-89.6); 762	1764; <0.001
ANC (10^3^/µL)	3.2 (2.4-4.2); 882	3.0 (2.2-4); 3623	4.4 (3-6.5); 462	5.7 (3.4-9.2); 482	5449; <0.001	3.0 (2.2-4.5); 29	3.3 (2.3-5.2); 549	6.6 (3.8-9.7); 424	8.3 (5.5-11.5); 762	1764; <0.001
Lymphocytes (%)	31.8 (25.9-37.3); 882	31.0 (24-37.7); 3623	18.8 (11.1-26.5); 462	12.5 (6.4-22.4); 482	5449; <0.001	28.8 (26.4-35.9); 29	25.6 (17.8-33.7); 549	11.0 (7.2-18.3); 424	7.5 (4.6-12.5); 762	1764; <0.001
ALC (10^3^/µL)	1.8 (1.5-2.3); 882	1.6 (1.2-2.1); 3623	1.2 (0.8-1.6); 462	0.9 (0.6-1.4); 482	5449; <0.001	1.8 (1.2-2.4); 29	1.3 (1-1.7); 549	0.9 (0.6-1.2); 424	0.7 (0.5-1); 762	1764; <0.001
Eosinophils (%)	2.2 (1.2-4); 882	1.5 (0.6-2.8); 3623	0.4 (0.1-1.5); 462	0.1 (0-0.7); 482	5449; <0.001	1.7 (1-3.5); 29	0.8 (0.2-2); 549	0.1 (0-0.4); 424	0.1 (0-0.2); 762	1764; <0.001
AEC (10^3^/µL)	0.1 (0.1-0.2); 882	0.1 (0-0.2); 3623	0.0 (0-0.1); 462	0.01 (0-0.1); 482	5449; <0.001	0.1 (0.1-0.2); 29	0.0 (0-0.1); 549	0.01 (0-0); 424	0.01 (0-0); 762	1764; <0.001
Monocytes (%)	6.4 (5.2-7.8); 882	6.7 (5.4-8.1); 3623	6.0 (4.4-7.6); 462	5.1 (3.6-7); 482	5449; <0.001	6.5 (5.2-7.5); 29	6.7 (5.3-8.5); 549	5.2 (3.9-6.8); 424	4.4 (3.3-5.8); 762	1764; <0.001
AMC 10^3^/µL	0.4 (0.3-0.5); 882	0.4 (0.3-0.5); 3623	0.4 (0.3-0.5); 462	0.38 (0.3-0.5); 482	5449; 0.11	0.3 (0.2-0.5); 29	0.4 (0.3-0.5); 549	0.4 (0.3-0.6); 424	0.4 (0.3-0.6); 762	1764; 0.01
Basophils (%)	0.9 (0.5-1.5); 882	0.7 (0.4-1.5); 3623	0.5 (0.3-0.9); 462	0.4 (0.2-1); 482	5449; <0.001	0.5 (0.3-0.7); 29	0.5 (0.3-0.7); 549	0.3 (0.2-0.5); 424	0.3 (0.2-0.5); 762	1764; <0.001
ABC (10^3^/µL)	0.1 (0-0.1); 882	0.0 (0-0.1); 3623	0.0 (0-0.1); 462	0.03 (0-0.1); 482	5449; <0.001	0.03 (0-0); 29	0.03 (0-0); 549	0.03 (0-0); 424	0.03 (0-0); 762	1764; 0.34
NLR (ratio)	1.7 (1.3-2.4); 882	1.8 (1.3-2.6); 3623	3.8 (2.3-7.2); 462	6.3 (3-13.4); 482	5449; <0.001	2.0 (1.3-2.4); 29	2.5 (1.6-4); 549	7.3 (3.9-11.9); 424	11.2 (6.3-19.6); 762	1764; <0.001
PLR (Ratio)	120.2 (87-157); 882	121.9 (89.3-171.1); 3623	187.5 (124.8-304.1); 462	223.3 (138.5-382); 482	5449; <0.001	107.5 (95-143.9); 29	152.4 (102.4-217.2); 549	287.3 (173.3-435.5); 424	325.6 (202.6-513.7); 762	1764; <0.001
LMR (ratio)	4.8 (3.7-6.3); 882	4.5 (3.3-5.9); 3623	3.1 (2-4.3); 462	2.6 (1.5-3.9); 482	5449; <0.001	5.0 (3.9-6.2); 29	3.6 (2.5-5); 549	2.2 (1.3-3.5); 424	1.7 (1-2.8); 762	1764; <0.001
SII (Ratio)	369.7 (236.1-563.9); 882	358.0 (225.3-573.8); 3623	793.8 (446.7-1797); 462	1315.0 (532.4-3083.8); 482	5449; <0.001	328.4 (211.2-498.2); 29	477.9 (282.1-950.9); 549	1810.0 (764.8-3626.9); 424	2664.8 (1259.8-5368.7); 762	1764; 0.05
NMR (Ratio)	8.3 (6.4-10.7); 882	8.2 (6.1-10.8); 3623	11.6 (8.1-17.5); 462	15.0 (9.5-24.4); 482	5449; <0.001	9.6 (5.8-12.2); 29	9.2 (6.4-12.8); 549	14.9 (10.9-20.5); 424	18.9 (13.9-26.1); 762	1764; <0.001
TBIL (mg/dL)	0.6 (0.5-0.8); 921	0.6 (0.4-0.8); 3725	0.5 (0.4-0.7); 474	0.5 (0.4-0.7); 490	5610; <0.001	0.5 (0.3-0.7); 30	0.4 (0.3-0.6); 552	0.5 (0.4-0.6); 426	0.5 (0.4-0.8); 769	1777; <0.001
DBIL (mg/dL)	0.2 (0.2-0.3); 921	0.2 (0.2-0.3); 3728	0.2 (0.1-0.3); 474	0.2 (0.2-0.3); 491	5614; 0.09	0.2 (0.1-0.3); 30	0.2 (0.1-0.2); 552	0.2 (0.2-0.3); 426	0.2 (0.2-0.4); 769	1777; <0.001
IBIL (mg/dL)	0.4 (0.3-0.6); 921	0.4 (0.3-0.5); 3725	0.3 (0.2-0.5); 474	0.3 (0.2-0.4); 490	5610; <0.001	0.3 (0.2-0.4); 30	0.3 (0.2-0.4); 552	0.3 (0.2-0.4); 426	0.3 (0.2-0.4); 769	1777; 0.01
SGPT/ALT (U/L)	34.3 (21-55); 921	36.5 (23.6-59); 3728	40.0 (27-75); 473	40.0 (25.7-67.5); 491	5613; <0.001	32.5 (20.3-50.8); 30	35.0 (23-66); 552	52.0 (31-96); 426	53.0 (31-93); 769	1777; <0.001
SGOT/AST (U/L)	31.0 (25-42); 921	33.8 (26-47); 3728	43.0 (29.6-67); 474	45.0 (31-71); 491	5614; <0.001	34.5 (24-50.5); 30	35.0 (26.8-51); 552	49.0 (32-78); 426	56.0 (37-89); 769	1777; <0.001
ALP (IU)	85.0 (71-106); 921	82.0 (68-102); 3728	83.0 (66-110); 473	87.0 (68-121); 491	5613; 0.01	81.0 (63.3-95); 30	77.0 (63.8-95); 552	77.5 (60.3-102); 426	87.0 (67-119); 769	1777; <0.001
TP (g/dL)	7.0 (6.6-7.3); 921	6.8 (6.5-7.2); 3728	6.6 (6.2-6.9); 474	6.4 (5.9-6.8); 491	5614; <0.001	6.8 (6.5-7.3); 30	6.7 (6.3-7); 552	6.4 (6-6.8); 426	6.2 (5.8-6.6); 769	1777; <0.001
Albumin (g/dL)	4.4 (4.2-4.6); 921	4.3 (4.1-4.5); 3728	4.0 (3.7-4.2); 474	3.7 (3.3-4.1); 491	5614; <0.001	4.3 (4.1-4.6); 30	4.1 (3.8-4.4); 552	3.8 (3.6-4.1); 426	3.6 (3.4-3.9); 769	1777; <0.001
Globulin (g/dL)	2.6 (2.3-2.8); 921	2.6 (2.3-2.8); 3728	2.6 (2.3-2.8); 474	2.6 (2.3-2.9); 491	5614; 0.47	2.5 (2.3-2.8); 30	2.5 (2.3-2.8); 552	2.5 (2.3-2.9); 426	2.6 (2.3-2.9); 769	1777; 0.03
A/G Ratio	1.7 (1.5-1.9); 921	1.7 (1.5-1.9); 3728	1.5 (1.3-1.8); 474	1.5 (1.2-1.7); 491	5614; <0.001	1.7 (1.5-1.9); 30	1.6 (1.4-1.8); 552	1.5 (1.3-1.7); 426	1.4 (1.2-1.6); 769	1777; <0.001
Urea (mg/dL)	19.3 (13-25); 915	20.0 (15-25.7); 3717	30.0 (21.4-40.7); 474	34.2 (21.4-57.8); 488	5594; <0.001	21.4 (19-28); 30	24.0 (19.3-32.1); 551	36.0 (25.7-49.2); 426	47.1 (34-66.3); 767	1774; <0.001
Creatinine (mg/dL)	0.7 (0.6-0.9); 917	0.8 (0.7-0.9); 3720	0.8 (0.7-0.9); 474	0.8 (0.7-1.1); 489	5600; <0.001	0.7 (0.6-0.8); 30	0.7 (0.6-0.8); 551	0.7 (0.6-0.9); 426	0.8 (0.7-1); 768	1775; <0.001
Uric Acid (mg/dL)	5.1 (4.1-6.1); 917	5.0 (4.1-5.9); 3718	4.6 (3.7-5.7); 474	5.0 (3.9-6.4); 489	5598; <0.001	4.7 (3.8-5.3); 30	4.6 (3.6-5.5); 551	4.4 (3.5-5.5); 426	4.8 (3.7-6.2); 768	1775; 0.03
Calcium (mg/dL)	9.1 (8.8-9.5); 917	9.0 (8.6-9.3); 3720	8.8 (8.4-9.1); 474	8.5 (8.1-8.9); 489	5600; <0.001	8.8 (8.4-9.2); 30	8.6 (8.4-9); 551	8.5 (8.1-8.7);426	8.3 (7.9-8.6); 768	1775; <0.001
Phosphorus (mg/dL)	3.1 (2.7-3.7); 916	2.9 (2.5-3.4); 3720	2.9 (2.4-3.4); 474	3.0 (2.5-3.6); 489	5599; <0.001	3.1 (2.7-4); 30	3.1 (2.6-3.5); 551	2.9 (2.4-3.5); 426	3.0 (2.5-3.6); 767	1774; 0.02
Sodium (mmol/L)	140.0 (139-141); 917	140.0 (138-141); 3720	138.0 (136-140); 474	138.0 (135-141); 489	5600; <0.001	138.0 (138-139.8); 30	139.0 (137-140); 551	138.0 (135-140); 426	138.0 (135-141); 768	1775; <0.001
Potassium (mmol/L)	4.3 (4-4.6); 916	4.3 (3.9-4.6); 3720	4.4 (4-4.8); 474	4.5 (4.1-4.9); 489	5599; <0.001	4.4 (4.2-4.8); 30	4.3 (4-4.7); 551	4.6 (4.1-5); 426	4.7 (4.2-5.1); 768	1775; <0.001
Chloride (mmol/L)	105.0 (103-106); 917	105.0 (103-106); 3720	103.0 (100-105); 474	103.0 (99-106); 489	5600; <0.001	104.0 (103-105.8); 30	103.0 (101-105); 551	101.0 (99-104); 426	101.0 (98-104); 768	1775; <0.001
Ferritin (ng/mL)	94.8 (44.6-185.8); 449	136.4 (61-283.8); 2350	307.9 (154.3-686.4); 429	555.3 (222-1123.1); 441	3669; <0.001	84.9 (30.4-194.8); 11	163.6 (68.5-430.8); 242	682.4 (329.2-1108.4); 200	856.4 (455.9-1547.9); 450	903; <0.001
LDH (U/L)	223.0 (201-255); 358	243.0 (207-297); 1929	326.0 (252-438); 409	381.0 (282-525); 373	3069; <0.001	212.0 (189.8-245.5); 28	252.0 (210-310); 529	390.5 (303-487.5); 408	528.0 (399.3-717.3); 730	1695; <0.001
CRP (U/L)	0.1 (0-0.4); 584	0.3 (0.1-1.3); 2809	3.6 (1.2-7.9); 468	7.7 (2.1-14.7); 467	4328; <0.001	0.2 (0-0.6); 30	0.8 (0.2-1.7); 543	4.1 (2.3-6); 422	9.2 (4.9-14.8); 758	1753; <0.001
Procalcitonin (ng/mL)	0.0 (0-0); 188	0.0 (0-0); 1242	0.0 (0-0.1); 280	0.1 (0-0.3); 258	1968; <0.001	0.01 (0-0); 1	0.03 (0-0.1); 142	0.04 (0-0.1); 127	0.1 (0-0.2); 351	621; <0.001
IL-6 (pg/mL)	1.3 (0-3.6); 221	3.4 (0.9-12); 1377	6.7 (1.9-24.7); 342	13.8 (3.2-47.2); 304	2244; <0.001	2.2 (0.7-5.4); 5	5.1 (2.4-12.1); 264	10.6 (4.3-27); 262	24.2 (8.9-77); 579	1110; <0.001
CRP-ALB (Ratio)	0.0 (0-0.1); 583	0.1 (0-0.3); 2808	0.9 (0.3-2); 468	2.2 (0.6-4.2); 467	4326; <0.001	0.04 (0-0.2); 30	0.2 (0.1-0.4); 540	1.1 (0.6-1.6); 420	2.6 (1.3-4.1); 757	1747; <0.001
APTT (Seconds)	32.6 (30.3-35.0); 479	32.2 (30.2-34.6); 2506	31.4 (29.4-34); 437	32.3 (29.4-35.4); 442	3864; <0.001	32.7 (29.5-35.6); 28	32.9 (30.1-36); 519	32.4 (29.6-34.9); 403	32.5 (29.6-36.1); 724	1674; 0.42
D-Dimer (ng/mL)	76.0 (50.0-128.0); 496	101.0 (60-175); 2547	191.5 (117.8-357); 436	282.0 (152-668); 437	3916; <0.001	115.5 (73.3-155.8); 26	142.0 (92.5-222.5); 499	234.0 (167.3-386.5); 386	459.0 (246.5-1448.5); 704	1615; <0.001
Fibrinogen (mg/dL)	294.0 (258.0-339.0); 493	316.0 (270-378); 2498	406.0 (328-485); 435	419.5 (331-509); 448	3874; <0.001	310.0 (284.8-354); 28	351.0 (307-401); 501	431.0 (377-494.5); 383	457.0 (390-528); 713	1625; <0.001
PT (Seconds)	12.1 (11.6-12.8); 489	12.1 (11.6-12.8); 2571	12.6 (11.8-13.6); 441	13.0 (12.1-14.2); 453	3954; <0.001	11.6 (11-12); 28	11.4 (10.9-12); 522	11.6 (10.9-12.4); 399	11.9 (11.2-13); 717	1666; <0.001
INR (Ratio)	1.1 (1.0-1.1); 487	1.1 (1-1.1); 2570	1.1 (1-1.2); 441	1.1 (1-1.2); 449	3947; <0.001	1.03 (1-1.1); 28	1.0 (0.9-1.1); 522	1.0 (0.9-1.1); 399	1.0 (1-1.1); 717	1666; <0.001
Glu-R (mg/dL)	84.0 (76.0-101.0); 811	90.0 (79-114); 3435	116.0 (91-168); 441	133.0 (97-200); 431	5118; <0.001	84.5 (80-94.3); 26	98.0 (83-126); 487	134.0 (101-205); 373	152.0 (112-230); 638	1524; <0.001
HbA1c (%)	5.6 (5.3-6.0); 262	5.7 (5.4-6.2); 1596	6.0 (5.6-6.9); 336	6.3 (5.7-7.6); 276	2470; 0.01	5.1 (4.9-5.3); 22	5.3 (5-6); 416	5.8 (5.4-6.9); 351	6.1 (5.5-7.3); 617	1406; <0.001

**Table 12:** Inter-COVID-19 wave (first wave versus second wave) comparison among disease severity groups The median value of severity groups in both waves is given in Table 9 Hb, Hemoglobin; HCT, hematocrit; RBC, red blood cell; TLC, total leucocyte count; PLT, platelet; MCV, mean corpuscular volume; MCH, mean corpuscular hemoglobin; MCHC, mean corpuscular hemoglobin concentration; RDW: red cell distribution width; ANC, absolute neutrophil counts; ALC, absolute lymphocyte counts; AMC, absolute monocyte counts; AEC, absolute eosinophil counts; ABC, absolute basophil counts; NLR, neutrophil-lymphocyte ratio; PLR, platelet-to-lymphocyte ratio; LMR, lymphocyte-to-monocyte ratio; NMR, neutrophil-to-monocyte ratio; SII, systemic immune-inflammation index; PT, prothrombin time; INR, international normalized ratio; aPTT, activated partial thromboplastin time; TBIL, total bilirubin; DBIL, direct bilirubin; IBIL, indirect bilirubin; ALT/SGPT, alanine aminotransferase/serum glutamic pyruvic transaminase; AST/SGOT, aspartate aminotransferase/serum glutamic oxaloacetic transaminase; ALP, alkaline phosphatase; TP, total protein; ALB, albumin; A/G, albumin/globulin; CRP/ALB, C-reactive protein/albumin; Glu-R, serum random glucose; HbA1c, glycated hemoglobin; COVID-19, coronavirus disease 2019; LDH, lactate dehydrogenase

	First Wave Versus Second Wave								
	Asymptomatic		Mild		Moderate		Severe		Total
	Total	P Value	Total	P Value	Total	P Value	Total	P Value	Total
Age (Years)	954	0.37	4295	0.23	905	0.12	1262	0.08	7417
Duration (Days)	954	<0.001	4295	<0.001	905	<0.001	1262	0.01	7416
Hb (g/dL)	945	0.82	4265	<0.001	895	0.01	1256	<0.001	7362
HCT (%)	945	0.77	4265	<0.001	895	<0.001	1256	<0.001	7362
RBC (10^6^/µL)	945	0.48	4265	0.3	895	0.01	1256	<0.001	7362
TLC (10^3^/µL)	945	0.27	4265	0.1	895	<0.001	1256	<0.001	7361
PLT (10^3^/µL)	945	0.06	4265	<0.001	895	<0.001	1256	<0.001	7361
MCV (fL)	945	0.17	4265	<0.001	895	0.35	1256	0.16	7362
MCH (pg)	945	0.41	4265	0.15	895	0.70	1256	0.40	7362
MCHC (g/dL)	945	0.85	4265	0.15	895	0.09	1256	0.13	7362
RDW (%)	945	0.03	4265	<0.001	895	0.01	1256	0.01	7361
Neutrophils (%)	911	0.14	4172	<0.001	886	<0.001	1244	<0.001	7213
Lymphocytes (%)	911	0.51	4172	<0.001	886	<0.001	1244	<0.001	7214
Eosinophils (%)	911	0.33	4172	<0.001	886	<0.001	1244	<0.001	7213
Monocytes (%)	911	0.59	4172	0.97	886	<0.001	1244	<0.001	7215
Basophils (%)	911	<0.001	4172	<0.001	886	<0.001	1244	<0.001	7213
ANC (10^3^/µL)	911	0.75	4172	<0.001	886	<0.001	1244	<0.001	7214
ALC (10^3^/µL)	911	0.34	4172	<0.001	886	<0.001	1244	<0.001	7213
AEC (10^3^/µL)	911	0.24	4172	<0.001	886	<0.001	1244	<0.001	7213
AMC (10^3^/µL)	911	0.13	4172	0.01	886	0.01	1244	<0.001	7213
ABC (10^3^/µL)	911	<0.001	4172	<0.001	886	<0.001	1244	<0.001	7213
NLR (Ratio)	911	0.30	4172	<0.001	886	<0.001	1244	<0.001	7213
PLR (Ratio)	911	0.63	4172	<0.001	886	<0.001	1244	<0.001	7214
LMR (Ratio)	911	0.74	4172	<0.001	886	<0.001	1244	<0.001	7214
SII (Ratio)	911	0.21	4172	0.14	886	0.04	1244	0.45	7213
NMR (Ratio)	911	0.17	4172	<0.001	886	<0.001	1244	<0.001	7213
TBIL (mg/dL)	951	0.04	4277	<0.001	900	<0.001	1259	0.29	7387
DBIL (mg/dL)	951	0.44	4280	<0.001	900	0.28	1260	<0.001	7392
IBIL (mg/dL)	951	0.01	4277	<0.001	900	<0.001	1259	0.13	7387
SGPT/ALT (U/L)	951	0.70	4280	0.37	899	<0.001	1260	<0.001	7391
SGOT/AST (U/L)	951	0.64	4280	0.10	900	0.01	1260	<0.001	7392
TP (g/dL)	951	0.25	4280	<0.001	900	<0.001	1260	0.03	7391
ALP (IU)	951	0.19	4280	<0.001	899	0.01	1260	0.62	7390
Globulin (g/dL)	951	0.62	4280	0.33	900	0.50	1260	0.31	7392
A/G Ratio	951	0.60	4280	<0.001	900	0.05	1260	0.01	7392
Albumin (g/dL)	951	0.24	4280	<0.001	900	<0.001	1260	<0.001	7391
Urea (mg/dL)	945	0.04	4268	<0.001	900	<0.001	1255	<0.001	7368
Creatinine (mg/dL)	947	0.11	4271	<0.001	900	<0.001	1257	0.29	7375
Calcium (mg/dL)	947	<0.001	4271	<0.001	900	<0.001	1257	<0.001	7375
Phosphorus (mg/dL)	946	0.34	4271	<0.001	900	0.37	1256	0.72	7374
Sodium (mmol/L)	947	<0.001	4271	<0.001	900	0.02	1257	0.23	7375
Potassium (mmol/L)	946	0.21	4271	<0.001	900	0.01	1257	<0.001	7374
Chloride (mmol/L)	947	0.21	4271	<0.001	900	<0.001	1257	<0.001	7375
Uric Acid (mg/dL)	947	0.06	4269	<0.001	900	0.03	1257	0.20	7373
Ferritin (ng/mL)	460	0.65	2592	<0.001	629	<0.001	891	<0.001	4573
LDH (U/L)	386	0.20	2458	0.05	817	<0.001	1103	<0.001	4764
CRP (U/L)	614	0.45	3352	<0.001	890	0.25	1225	<0.001	6082
Procalcitonin (ng/mL)	189	0.48	1384	<0.001	407	0.85	609	0.65	2590
IL-6 (pg/mL)	226	0.33	1641	<0.001	604	<0.001	883	<0.001	3354
CRP/ALB (Ratio)	613	0.44	3348	<0.001	888	0.10	1224	<0.001	6074
APTT (Seconds)	507	0.95	3025	<0.001	840	0.03	1166	0.15	5539
D-Dimer (ng/mL)	522	0.02	3046	<0.001	822	<0.001	1141	<0.001	5531
Fibrinogen (mg/dL)	521	0.07	2999	<0.001	818	<0.001	1161	<0.001	5499
PT (Seconds)	517	<0.001	3093	<0.001	840	<0.001	1170	<0.001	5620
INR (Ratio)	515	0.04	3092	<0.001	840	<0.001	1166	<0.001	5613
Glu-R (mg/dL)	837	0.62	3922	<0.001	814	<0.001	1069	<0.001	6643
HbA1c (%)	284	<0.001	2012	<0.001	687	0.01	893	0.01	3876

## Discussion

SARS-CoV-2 is a multisystem disease involving a complex interplay of immunological, inflammatory, and coagulation cascades [4]. The SARS-CoV-2 virus entry into the target cells is facilitated by the interaction between the angiotensin-converting enzyme 2 (ACE2) and transmembrane serine protease 2 (TMPRSS2) of host cells with virus spike protein [4,9]. This can lead to a multiorgan injury affecting various tissues including the lungs (specifically alveolar epithelial type II cells), the pharynx, bone marrow cells, the kidneys, the myocardium, the neurological system, and the gastrointestinal tract, as reported in the literature [9]. The possible mechanisms of the multisystem involvement reported are ACE2-mediated cell damage, dysregulation of the immune response by cytokine storm, endothelial cell damage due to sepsis, or dysregulation of RAAS [4].

The overall COVID-19 mortality in India was reported to be 1.38% until August 2021 [2]. However, in the present study, the hospital mortality reported was 6.1% (461/7416) and severe disease in 17% (1262/7416). Males were more prone to severe disease and mortality probably because males have abundant ACE2 receptors than females as concluded in our study also [5,6].

In the present study, it was reported that the unadjusted relative risk for older age (>52 years) was significantly associated with increased disease severity and an independent risk factor for mortality. This could be due to an age-dependent decline in immunity due to a decrease in T-cells and its subsets, as well as a predisposition to hypercoagulable and hyperactivated immune systems. Another theory is that with increasing age, there is a decline in sirtuin-2 levels, exacerbating SARS-CoV-2 infection and possibly leading to the hyperactivation of nucleotide-binding oligomerization domain, leucine-rich repeat and pyrin domain-containing protein 3 (NLRP3), which in turn trigger cytokine storm in elderly patients [10]. Leulseged et al. [5] and Leulseged et al. [6] also concluded that age (40-59 years and ≥60 years) is associated with severe disease, and ≥50 years of age is associated with unfavorable outcomes.

In the current study, hematological parameters that were COVID-19 severity predictors were neutrophils (>81%), lymphocytes (25.4-20.6, 20.5-17.5, and 17.4), ALCs (1.38-1.13 and 1.12-0.91), and AECs (0.90 and 0.03-0.02). According to a study by Monárrez-Espino et al., neutrophilia (>7.5×103/L) was an independent predictor of the severity of COVID-19 [11]. Bellan et al. [12] and Yang et al. [13] also identified NLR as a predictor of severity and mortality (NLR of >4.6 and NLR of >3.3, respectively); however, in the present study, NLR (mortality cutoff, >4.87; severity cutoff, >6.81) was not an independent risk factor for both COVID-19 mortality and severity. Lymphopenia, eosinopenia, basopenia, and monocytopenia could be due to hampered cellular immunity as a result of direct viral toxicity or cytokine-induced apoptosis or atrophy of the lymphoid organs [14]. Leucocytosis and neutrophilia may potentially result from a cytokine storm or subsequent bacterial infection. These patients had a higher propensity to have a serious illness and unfavorable outcome [6,13-18].

Coagulation disorders such as pulmonary microthrombosis and disseminated intravascular coagulation (DIC) are quite frequent among COVID-19 patients with severe diseases and are associated with adverse outcomes [14]. The potential causes for these coagulation disorders include endothelial damage and dysfunction, thrombo-inflammation, platelet activation, and increased blood flow stasis and viscosity, which contribute to thrombosis. Furthermore, there may be the inhibition of fibrinolysis, impairment of coagulation factor production due to liver damage, formation of neutrophil extracellular traps (NETs) through the interaction of activated platelets and neutrophils, stimulation of the complement system, and enhancement of phagocytosis [14,19]. COVID-19 coagulopathy, unlike regular DIC, has higher fibrinogen levels, mildly raised PT and aPTT, and thrombocytosis but without any significant bleeding [7,20]. In our study, we also found that all coagulation parameters (PT, INR, aPTT, D-dimer, and fibrinogen), along with platelet count, were raised among those with severe disease and had higher mortality. D-dimer (mortality, >268 ng/mL; severity, >350 ng/mL) and fibrinogen (mortality, >403 mg/dL; severity, >425 mg/dL) were independent predictors for severity of the disease, as well as for mortality, whereas INR of >1.18 was and independent predictor for only mortality, even through it was significantly associated with disease severity (>1.16) but insignificant in multivariate regression analysis [20]. COVID-19 patients with INR of >1.2 and D-dimer levels of >280 ng/mL are associated with a worse prognosis [17,21].

Viral replication and tissue damage cause the release of inflammatory mediators such as IL-6, IL-4, and IL-10, which lead to an activation of antigen-presenting cells or hyperactivity of T lymphocyte, which in turn activates the immune system and exaggerated cytokine production, resulting in cytokine storm [7,18]. In cytokine storms, various acute phase reactants such as C-reactive protein (CRP), ferritin, and LDH are released resulting in multiorgan dysfunction, secondary infection, sepsis, and death in COVID-19 patients [22]. Procalcitonin is one of the indicators of sepsis. In our study, procalcitonin of >0.1 ng/mL was an independent mortality predictor. Ferritin (mortality, >483.89 ng/mL; severity, >445.4 ng/mL), IL-6 (mortality, >8.8 pg/mL; severity, >28.6 pg/mL), and LDH (mortality, >393 U/L; severity, >479 U/L) were independent predictors for both mortality and severity. Few articles published concluded that COVID-19 patients with procalcitonin levels of >0.5 ng/mL have five times higher risk of developing severe disease and mortality, whereas Mahendra et al. [10] reported that serum ferritin of >450 μg/L was independently associated with mortality [23].

SARS-CoV-2 may directly damage hepatocytes, cholangiocytes, and renal tubular cells by ACE-induced direct cytotoxicity resulting in microemboli in hepatic vessels [4,19]. Few studies, as well as our study, reported that parameters indicating liver damage were hypoalbuminemia, bilirubinemia, elevated liver enzymes, and raised LDH levels in patients with both non-survivors and severe COVID-19 [4,7,14,19-23]. In our study, we reported that TBIL of >0.54 mg/dL and albumin of ≤3.5 g/dL are independent severity predictors. We also concluded that A/G ratio of ≤1.47 and CRP/ALB ratio of >1.78 were independent predictors of mortality and severity, respectively. Published studies also concluded that albumin/globulin (A/G) ratio and CRP/albumin (CRP/ALB) ratio were more accurate than each parameter separately and the independent risk factor for mortality at 90 days in septic patients of CRP/ALB ratio is >5.09 [24,25].

Abnormal kidney function such as raised creatinine, urea, and uric acid levels in fatal and severe cases is an indicator of renal damage reported by published studies and in our study [4,11,14,18]. However, in our study, the cutoff of blood urea nitrogen (BUN) was >36 mg/dL for mortality and >33.7 mg/dL for severity, which were associated with increased odds, although they were not independent predictors.

Electrolyte abnormalities such as hypocalcemia, hyperphosphatemia, hyponatremia/hypernatremia, hyperkalemia, and hypochloremia are usual manifestation in non-survivors and severe COVID-19 patients as documented in published studies, and these findings are consistent with our findings [6,19,22]. Chloride of ≤101 mmol/L was found to be an independent risk factor for mortality in the present study. These electrolyte imbalances can be caused by gastrointestinal manifestation such as nausea or diarrhea, which leads to dehydration, or by increased angiotensin levels, which promote potassium excretion and causes hypokalemia, whereas hyperkalemia can result from increased cell turnover or by impairing epithelial sodium channels causing potassium retention [4,19].

COVID-19 patients with diabetes mellitus are at higher risk of developing severe disease and mortality [4]. This could be due to damage of pancreatic B cells leading to reduced insulin production contributing to hyperglycemia and acidosis [4]. In our study, we also concluded that random glucose and HbA1c were unadjusted risk factors for mortality and disease severity.

The overall disease severity and mortality were higher in the second wave than in the first wave, but laboratory biomarkers showed a similar trend across both waves in our study. This could be due to the COVID-19 virus strain B.1.617.2, also known as the delta variant, being more transmissible and potentially more fatal [26,27]. Few authors had reported similar finding as in our study that lymphopenia, raised NLR, and moderately elevated inflammatory markers (CRP, ferritin, IL-6, and LDH), along with raised D-Dimer, were significantly higher in the second wave than in the first wave and can be utilized as prognostic and disease acuity indicators on admission in the second wave [26]. Vijay et al. [28] and Budhiraja et al. [26] reported that more patients in the second wave had secondary bacterial and fungal infection, resulting in an elevated procalcitonin. The majority of the patients in the first wave were admitted to hospitals owing to government policies of institutional isolation for COVID-19-infected patients, while the majority of the patients in the second wave were sicker and required oxygen therapy. This could lead to differences in some laboratory parameters at hospital admission with regard to disease severity, as well as the majority of laboratory biomarkers on comparisons among the both waves showing significant differences among recovered patients [27].

Even though our study was a comprehensive observation study including the largest cohort of patients and laboratory parameters, it had few limitations such as it was a single center and retrospective study based on hospital and laboratory records.

## Conclusions

In the present study, we analyzed laboratory parameters that could be COVID-19 severity and mortality predictors along with their optimal cutoff. We concluded that only 14 laboratory biomarkers out of 55 were associated with either a poor outcome, a severe disease, or both. The significant risk factors for mortality include advanced age (>52 years), ferritin (>483.89 ng/mL), A/G ratio (≤1.47), chloride (≤101 mmol/L), LDH (>393 U/L), procalcitonin (>0.10 ng/mL), interleukin-6 (>8.8 pg/mL), fibrinogen (>403 mg/dL), international normalized ratio (INR) (>1.18), and D-dimer (>268 ng/mL). For severe cases of COVID-19, neutrophils (>81%), lymphocyte (≤25.4%), absolute lymphocyte count (≤1.38×10^3^/μL), absolute eosinophil count (≤0.03×10^3^/μL), total bilirubin (≥0.51 mg/dL), A/G ratio (≤1.49), albumin (≤4.2 g/dL), ferritin (≥445.4 mg/dL), LDH (≥479 U/L), IL-6 (≥28.6 pg/mL), CRP/ALB ratio (≥1.78), D-dimer (≥237 ng/mL), and fibrinogen (≥425 mg/dL) are also crucial biomarkers.

Our study also found that during the second wave of COVID-19, many of these baseline laboratory parameters were significantly higher, which could be one reason for the increased mortality rate in India during that time. These laboratory parameters can provide critical clinical information about COVID-19 patients at admission and are readily available, simple, and inexpensive. By utilizing all these parameters in emergency settings, healthcare providers can improve the effectiveness of treatment and reduce mortality. These findings can also help develop risk scores to help clinicians identify patients at high risk of mortality.
